# Detection of *Mycobacterium ulcerans* by real-time PCR with improved primers

**DOI:** 10.1186/s41182-016-0028-3

**Published:** 2016-08-19

**Authors:** Noriko Shinoda, Hajime Nakamura, Mineo Watanabe

**Affiliations:** 1 Graduate School of Infection Control Sciences, Kitasato University, 5-9-1 Shirokane, Minato-ku, Tokyo 108-8641 Japan; 2 Research Institute of Tuberculosis, Japan Anti-Tuberculosis Association, 3-1-24 Matsuyama, Kiyose, Tokyo 204-8533 Japan; 3 Kitasato Institute for Life Sciences, Kitasato University, 5-9-1 Shirokane, Minato-ku, Tokyo 108-8641 Japan

**Keywords:** Buruli ulcer, *Mycobacterium ulcerans*, Diagnosis, Real-time PCR

## Abstract

**Background:**

Buruli ulcer is a severe skin disease caused by *Mycobacterium ulcerans*. Real-time PCR targeting the IS*2404* sequence has been used as a reliable and rapid method for the diagnosis of Buruli ulcer and detection of *M. ulcerans* in the environment. The genome of *M. ulcerans* contains hundreds of IS*2404* copies, which have variability in certain sequences. Therefore, the design of new primers specific to conserved IS*2404* regions may potentially improve the sensitivity of *M. ulcerans* detection and, consequently, the diagnosis of Buruli ulcer, thus ensuring timely treatment of the disease.

**Results:**

In silico analysis indicates that DNA sequences of the IS*2404* elements are highly variable within a single strain. As the binding sites of conventional IS*2404*-specific primers used for *M. ulcerans* detection contain polymorphic sequences, we designed new primers, which enabled the detection of *M. ulcerans* by real-time PCR with higher sensitivity and similar specificity with respect to that of conventional primers. However, the increase in sensitivity with the new primers depended on the *M. ulcerans* strain.

**Conclusions:**

The results suggest that real-time PCR based on the new primers could improve Buruli ulcer diagnosis and *M. ulcerans* detection in environmental samples.

**Electronic supplementary material:**

The online version of this article (doi:10.1186/s41182-016-0028-3) contains supplementary material, which is available to authorized users.

## Background

Buruli ulcer, a progressive skin disease caused by *Mycobacterium ulcerans*, is prevalent in more than 30 countries, with especially high incidence in West Africa [1–4]. The source of *M. ulcerans* infection is still unknown; however, the bacteria has been detected in aquatic insects [5–8] and the disease mostly occurs in people who live near still water areas, suggesting that contaminated waters may act as a reservoir of *M. ulcerans* [5, 9].

In the early stages of the disease, papules, nodules, plaques, and edema are observed in the skin, followed by progressive ulceration eroding to subcutaneous layers and even bones [10, 11]. In extreme cases, patients may suffer contracture deformity and even amputation [2, 4]; therefore, early diagnosis and treatment is important for Buruli ulcer control. Common diagnostic methods include smear microscopy, histopathology, and culture; however, they have limited sensitivity or are time-consuming [12]. Thus, it is necessary to develop simple and rapid tools that provide sufficient detection sensitivity to confirm the diagnosis of Buruli ulcer.

Several PCR methods for the detection of *M. ulcerans* have been reported; among them, the most widely used is based on targeting the IS*2404* repeat sequence, which encodes a transposase and which is unique to *M. ulcerans* genome, where it occurs over 200 times [13–15]. Since other targets such as genes encoding 16S rRNA [16], hsp65 [17] and the ketoreductase domain of mycolactone polyketide synthase [14, 18], or IS*2606* [14, 18] have much lower copy numbers than IS*2404*, the sensitivity of their detection by PCR is lower than that of IS*2404* [14, 15, 18]. Moreover, of these sequences, only IS*2404* is specific for *M. ulcerans* [14, 15, 18]. IS*2404* is also targeted by real-time PCR and loop-mediated isothermal amplification, which are more rapid and sensitive methods than gel-based conventional PCR and which have been recently applied for the detection of *M. ulcerans* [18–20]. Currently, real-time PCR is the gold standard method to confirm the presence of *M. ulcerans* [14, 15, 21].

Previous studies have used several primer sets for the amplification of IS*2404* [14, 15, 22]; however, most of them were designed for gel-based conventional PCR [18] and may not be suitable for a more sensitive real-time PCR. Our in silico analysis indicates that the sequence of the IS*2404* elements is highly variable and that the primers commonly used for IS*2404* amplification are based on the polymorphic regions. Therefore, in this study, we designed and validated a new set of primers highly specific for stable sequences conserved among IS*2404* copies as well as *M. ulcerans* strains with the aim to improve the sensitivity of *M. ulcerans* detection by real-time PCR.

## Methods

### Software for genetic analysis

Genetyx version 13 (Genetyx, Shibuya, Tokyo, Japan) was used for homology analysis and primer design. The alignment of IS*2404* elements was performed by Genetyx version 13 using the algorithm of the MUSCLE program [23].

### Bacterial strains and culture conditions

*M. ulcerans* strains used in this study are listed in Table 1. Strains Agy99 and TMC1615 were provided by Dr. Small (University of Tennessee, USA), and GTC16404, GTC16405, and GTC16406 were obtained from the GTC collection of Gifu University (Japan). ATCC strains 19423 and 33728 were purchased from the American Type Culture Collection (Manassas, VA, USA). Other *Mycobacterium* species were provided by Dr. Saito (Shimane University, Japan).

**Table 1:** *M. ulcerans* strains and *Mycobacterium* species used in this study

Species	Strain	Country of isolation	Year
*M. ulcerans*	Agy99	Ghana	1999
	TMC1615	Malaysia	1960s
	ATCC19423	Australia	1981
	ATCC33728	Japan	1980
	GTC16404	Japan	2010
	GTC16405	Japan	2007
	GTC16406	Japan	2011
*M. tuberculosis*	H37Rv	US	1934
*M. kansasii*	KHS-001	Japan	2000s
*M. avium*	AVHS-001	Japan	2000s
*M. intracellulare*	4-1974	Japan	2000s
*M. abscessus*	ABHS-001	Japan	2000s
*M. scrofulaceum*	CTM35840	–	–
*M. smegmatis*	ATCC700084	–	1990

*En dash*: information not available

Mycobacteria were grown in Middlebrook 7H9 broth (BD Biosciences, Sparks, MD, USA) supplemented with 0.05 % (*w*/*v*) Tween 80 and 10 % (*v*/*v*) OADC Enrichment (BD Biosciences).

### DNA extraction and purification

Bacteria were collected by centrifugation at 16,200×*g* for 2 min and resuspended in a solution containing 20 μl of 0.5 M NaOH, 4 μl of 10 % sodium dodecyl sulfate, and 180 μl of distilled water. The cell suspension was heated at 95 °C for 15 min, cooled to room temperature, and thoroughly mixed with 200 μl of phenol/chloroform (1:1). After centrifugation at 16,200×*g* for 5 min, the aqueous phase was transferred to a new tube and the extraction was repeated. Then, 20 μg of glycogen, 16 μl of 5 M NaCl, and 800 μl of 100 % ethanol were added to the pooled aqueous phases, and the mixture was centrifuged at 16,200×*g* for 15 min. The pellet was collected, mixed with 500 μl of 70 % ethanol, and the sample was centrifuged for 1 min. The final pellet was resuspended in 50 μl of distilled water.

### Real-time PCR

Real-time PCR was performed as described by Fyfe et al. [18]. The method is recommended by the World Health Organization (WHO) for *M. ulcerans* detection [14, 21] and is based on primers IS2404TF and IS2404TR and probe IS2404TP [18]. Alternatively, we used our newly designed primers IS2404KF and IS2404KR and probe IS2404KP. Primer and probe sequences are listed in Table 2. The reactions were performed in a total volume of 10 μl containing 5 μl of THUNDERBIRD Probe qPCR Mix (TOYOBO, Osaka, Japan), 0.5 μM of each primer, 0.2 μM of the probe, and 10 ng of purified *M. ulcerans* genomic DNA. The cycling conditions were as follows: 1 cycle of 95 °C for 60 s, and 35 cycles of 95 °C for 15 s and 60 °C for 60 s. The threshold cycle (Ct) for each sample was automatically calculated by the C1000 manager software version 1.0 (Bio-Rad Laboratories, Hercules, CA, USA).

**Table 2:** Primers and probes for real-time PCR

Primer or probe	Sequence (5′–3′)
IS2404TF	AAAGCACCACGCAGCATCT
IS2404TR	AGCGACCCCAGTGGATTG
IS2404TP	FAM-CGTCCAACGCGATC-BHQ1
IS2404KF	TCTCGTGTCGGTGTTC
IS2404KR	TGACGACCTGGGTATG
IS2404KP	FAM-AATGAAATTCCCTGCGT-MGB

IS2404TF, IS2404TR, and IS2404TP were described by Fyfe et al. [18]

*FAM* fluorescein amidite, *BHQ1* black hole quencher 1, *MGB* minor groove binder

### Statistical analysis

Statistical analysis was performed using the GraphPad Prism software version 6 (GraphPad Software, LA Jolla, CA, USA). The differences between samples were analyzed by the Student’s *t* test, and differences were considered statistically significant at a *p* value of 0.05.

## Results

### IS*2404* sequence variations

The genome of *M. ulcerans* strain Agy99 (gene accession number: CP000325) contains 249 IS*2404*-like elements [13]. For the purpose of this study, 212 sequences with high homology to the transposase gene of IS*2404* (MUL_0099) determined by BLASTN search were retrieved from the Agy99 genome and aligned with the transposase sequence used as a reference.

**Fig. 1:**
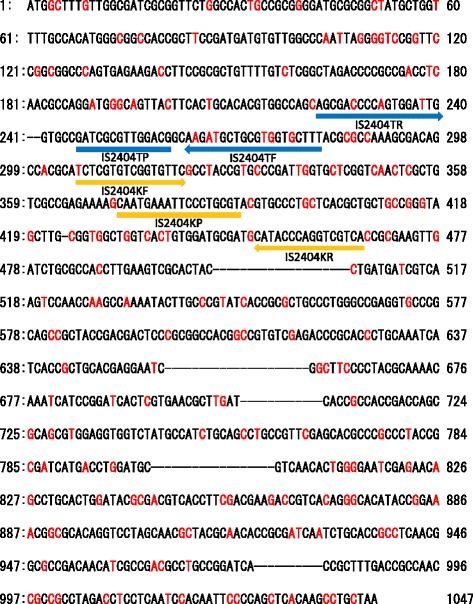
Polymorphism of the IS*2404* elements in the genome of *M. ulcerans* strain Agy99. Two hundred and twelve IS*2404*-like sequences were compared with the transposase-encoding gene (MUL_0099 gene) used as an IS*2404* reference sequence. *Red characters* indicate polymorphic sites; *hyphens/dashes* indicate gaps. *Arrows* and *lines* indicate positions of primers and probes, respectively; *blue* and *yellow colors* mark conventional and new, respectively, primers and probes

The results revealed numerous gaps and sequence dissimilarities at the nucleotide level among the compared IS*2404* elements (Fig. 1 and Additional file 1: Figure S1); in particular, sequence polymorphism was detected in the regions targeted by the IS2404TF and IS2404TR primers (Fig. 1 and Additional file 1: Figure S1) commonly used for real-time PCR-based detection of *M. ulcerans* [18]. The high level of sequence variability in the IS*2404* elements may affect the accuracy of PCR-based *M. ulcerans* detection. Thus, our observations suggest that the sensitivity of *M. ulcerans* detection by real-time PCR may be improved by using a new primer/probe set targeting regions highly conserved among multiple IS*2404*-like elements.

### Sensitivity of the new primer/probe set for *M. ulcerans* detection

**Fig. 2:**
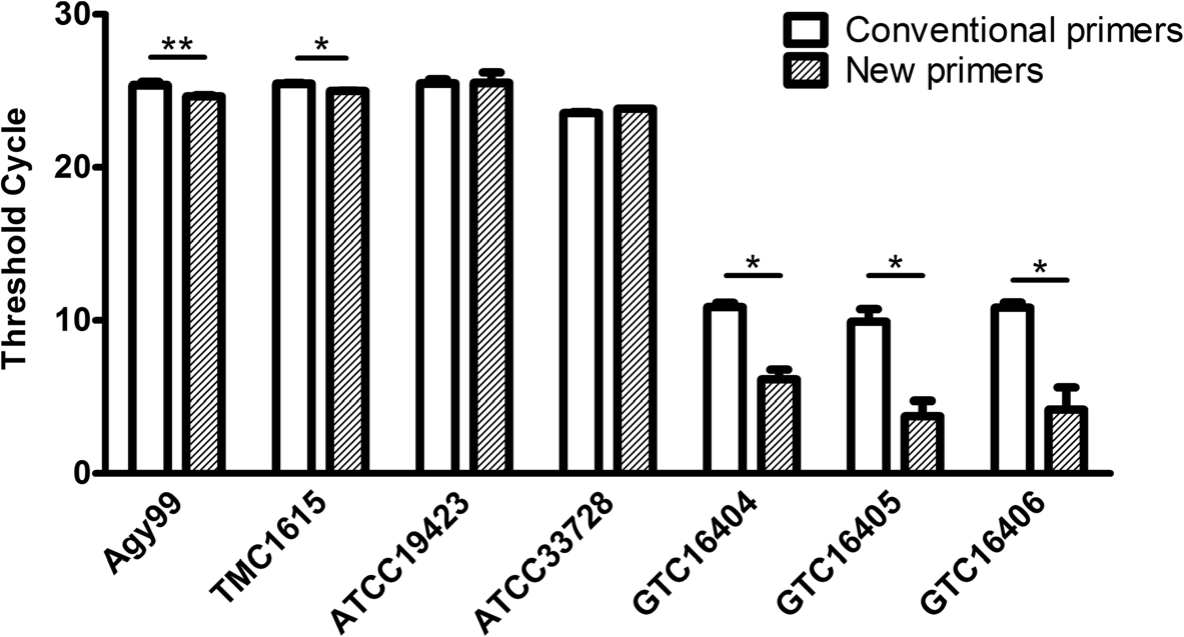
Sensitivity of the new IS*2404* primer/probe set for *M. ulcerans* detection. Real-time PCR was performed with the conventional primers and probe (IS2404TF, IS2404TR, and IS2404TP) or the new primers and probe (IS2404KF, IS2404KR, and IS2404KP) using 10 ng of genomic DNA of each *M. ulcerans* strain as a template. The results are expressed as the mean ± standard deviation of the threshold cycle based on three independent experiments; *p < 0.05, **p < 0.01 by Student’s *t* test

Based on the sequence alignment data, we designed a new set of PCR primers (IS2404KF and IS2404KR) and probe (IS2404KP) (Table 2) specific to stable IS*2404* regions (Fig. 1). The IS2404KF primer contained only one variable nucleotide at the 5′-terminus, while IS2404KR and IS2404KP did not have any sequence variability among different IS*2404* elements. We performed real-time PCR using the new primers and probe and compared their sensitivity and specificity with those of conventional primers (IS2404TF and IS2404TR) and probe (IS2404TP) (Fig. 2).

The use of the new primer/probe set enabled the detection of genomic DNA from most *M. ulcerans* strains using a smaller number of cycles than that required with the conventional set. PCR sensitivity was more significantly improved for three Japanese isolates (GTC16404, GTC16405, and GTC16406) than for Agy99 and TMC1615, while no changes were observed for ATCC19423 and ATCC33728. The results suggest that the new primer/probe set could improve the sensitivity of *M. ulcerans* PCR-based detection; however, the increase in sensitivity varied depending on the strain.

### Specificity of the new primer/probe set

**Fig. 3:**
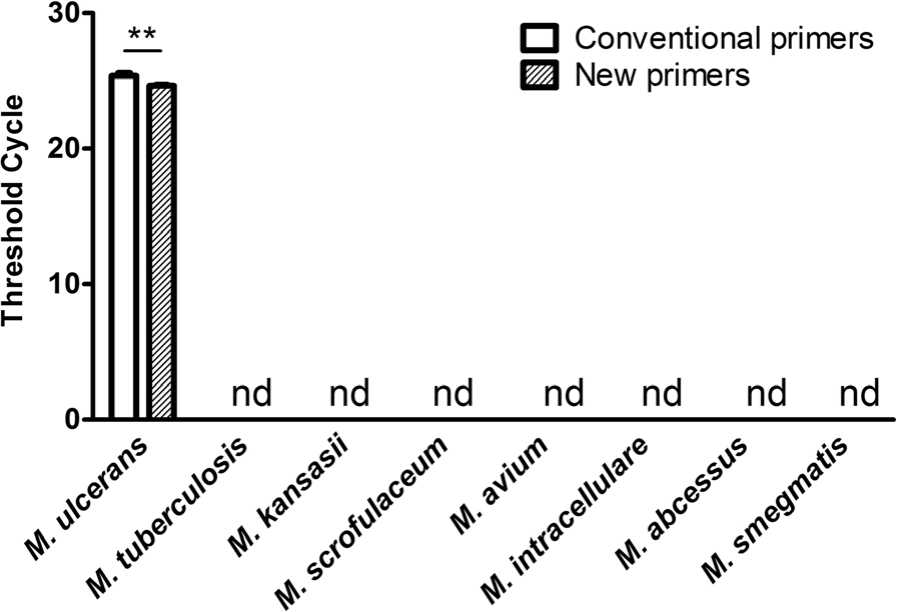
Specificity of the new IS*2404* primer/probe set for *M. ulcerans* detection. Real-time PCR was performed with the conventional primers and probe (IS2404TF, IS2404TR, and IS2404TP) or the new primers and probe (IS2404KF, IS2404KR, and IS2404KP) using 10 ng of genomic DNA of each *M. ulcerans* strain as a template. The results are expressed as the mean ± standard deviation of the threshold cycle based on three independent experiments; **p < 0.01 by Student’s *t* test, nd: not detected (threshold cycle over 33)

The detection specificity of real-time PCR based on the new set was evaluated using purified genomic DNA of *M. ulcerans* and seven other mycobacterial species (Table 1). As shown in Fig. 3, PCR with both conventional and new primer/probe sets detected only *M. ulcerans* DNA, indicating that the specificity of the new set was similar to that of the conventional set.

## Discussion

The IS*2404*-targeting PCR method, which enables rapid and sensitive detection of *M. ulcerans*, has been established as the gold standard for the diagnosis of Buruli ulcer [15, 18, 21] and is currently recommended by the WHO [21]. IS*2404* is a multi-copy insertion sequence encoding a 328-amino acid transposase [14], which is unique to *M. ulcerans* and is represented by 249 copies in its genome [13]. Because of the high frequency of occurrence of IS*2404* in the *M. ulcerans* genome, this element has been used as a target sequence in PCR-based detection of *M. ulcerans* infection. However, in this study, we revealed considerable sequence variability among the IS*2404* elements of a single *M. ulcerans* strain (Agy99) (Fig. 1 and Additional file 1: Figure S1); nucleotide polymorphism was also observed in the regions targeted by commonly used primers, which could affect the accuracy of Buruli ulcer diagnosis. Therefore, to increase the detection sensitivity of *M. ulcerans*, we designed a new primer/probe set specific for the regions highly conserved among IS*2404* copies. Compared with the conventionally used set, the new set provided an increased sensitivity and similar specificity of real-time PCR detection for most tested *M. ulcerans* strains (Figs. 2 and 3). Detection sensitivity with the new primer/probe set was particularly high for three Japanese isolates (GTC16404, GTC16405, and GTC16406); on the other hand, no changes were observed for the ATCC19423 and ATCC3372 strains. The results suggest that the new set could lead to better PCR-based detection of *M. ulcerans* than that with conventional primers, although the advantage may be strain dependent. In this study, we used only seven *M. ulcerans* isolates; more of them should be tested to comprehensively evaluate strain-specific differences in detection sensitivity using the new primer/probe set. In addition, it should be noted that clinical and environmental specimens could contain PCR inhibitors and contaminating DNA; therefore, the new set should be validated using a panel of clinical and environmental samples.

Since we observed sequence variability among IS*2404* copies of the same *M. ulcerans* strain, we hypothesized that the difference may also exist among the strains. As described in results, PCR sensitivity was more significantly improved by new primers for three Japanese isolates. The result suggests that the Japanese strains could have more sequence diversity in the binding regions of conventional primers than other strains. In this study, we used *M. ulcerans* isolates from geographically distant areas. Interestingly, for the strains from Africa (Agy99), Southeast Asia (TMC1615), and Australia (ATCC19423), which belong to the classical lineage [24], the new primer/probe set provided only moderate or no improvement of detection sensitivity, while for most Japanese strains, a significant increase in sensitivity was observed. Weihong et al. [25] demonstrated higher frequency of large chromosomal rearrangements in a Japanese strain compared to the classical lineage strains [25]. Since the IS*2404*-encoded transposase may be closely involved in genomic rearrangements, Japanese strains might harbor the IS*2404* elements carrying different types of polymorphisms compared to the classical lineage strains. To clarify why the new primers improved the sensitivity of *M. ulcerans* PCR detection, further analysis, including whole genome sequencing of each strain may be required. On the other hand, there is possibility that particular *M. ulcerans* isolates may escape IS*2404*-targeting PCR detection. Therefore, to provide sensitive and robust detection of *M. ulcerans*, it might be useful to perform multiplex PCR, which would target, along with IS*2404*, several other *M. ulcerans* sequences such as IS*2606* or ketoreductase domain in the genes encoding mycolactone polyketide synthase, as reported by Fyfe et al. [18].

Further sequence analysis of the IS*2404* elements is necessary to develop more sensitive methods for *M. ulcerans* diagnosis.

## Conclusions

The results of our study suggest that the new primer/probe set is more sensitive for PCR-based detection of *M. ulcerans* than the conventionally used set, suggesting that its application can improve the diagnosis of Buruli ulcer.

## Abbreviations

BHQ1, black hole quencher 1; FAM, fluorescein amidite; MGB, minor groove binder

## Additional file

Additional file 1: Figure S1. Sequence alignment of the IS*2404* elements from the genome of *M. ulcerans* strain Agy99. (PDF 1040 kb)

## References

[1] Einarsdottir T, Huygen K (2011). Buruli ulcer. Hum Vaccin.

[2] Sizaire V, Nackers F, Comte E, Portaels F (2006). *Mycobacterium ulcerans* infection: control, diagnosis, and treatment. Lancet Infect Dis.

[3] Weir E (2002). Buruli ulcer: the third most common mycobacterial infection. Can Med Assoc J.

[4] WHO (World Health Organization) (2014). Buruli ulcer.

[5] Aiga H, Amano T, Cairncross S, Adomako J, Nanas OK, Coleman S (2004). Assessing water-related risk factors for Buruli ulcer: a case-control study in Ghana. Am J Trop Med Hyg.

[6] Huygen K, Adjei O, Affolabi D, Bretzel G, Demangel C, Fleischer B (2009). Buruli ulcer disease: prospects for a vaccine. Med Microbiol Immunol.

[7] Johnson PD, Azuolas J, Lavender CJ, Wishart E, Stinear TP, Hayman JA (2007). *Mycobacterium ulcerans* in mosquitoes captured during outbreak of Buruli ulcer, southeastern Australia. Emerg Infect Dis.

[8] Marsollier L, Robert R, Aubry J, Saint Andre JP, Kouakou H, Legras P (2002). Aquatic insects as a vector for *Mycobacterium ulcerans*. Appl Environ Microbiol.

[9] Marsollier L, Stinear T, Aubry J, Saint Andre JP, Robert R, Legras P (2004). Aquatic plants stimulate the growth of and biofilm formation by *Mycobacterium ulcerans* in axenic culture and harbor these bacteria in the environment. Appl Environ Microbiol.

[10] Bamberger D, Jantzer N, Leidner K, Arend J, Efferth T (2011). Fighting mycobacterial infections by antibiotics, phytochemicals and vaccines. Microbes Infect.

[11] Silva MT, Portaels F, Pedrosa J (2009). Pathogenetic mechanisms of the intracellular parasite *Mycobacterium ulcerans* leading to Buruli ulcer. Lancet Infect Dis.

[12] Yeboah-Manu D, Asante-Poku A, Asan-Ampah K, Ampadu ED, Pluschke G (2011). Combining PCR with microscopy to reduce costs of laboratory diagnosis of Buruli ulcer. Am J Trop Med Hyg.

[13] Phillips R, Horsfield C, Kuijper S, Lartey A, Tetteh I, Etuaful S (2005). Sensitivity of PCR targeting the IS*2404* insertion sequence of *Mycobacterium ulcerans* in an Assay using punch biopsy specimens for diagnosis of Buruli ulcer. J Clin Microbiol.

[14] Stinear T, Ross BC, Davies JK, Marino L, Robins-Browne RM, Oppedisano F (1999). Identification and characterization of IS*2404* and IS*2606*: two distinct repeated sequences for detection of *Mycobacterium ulcerans* by PCR. J Clin Microbiol.

[15] Durnez L, Stragier P, Roebben K, Ablordey A, Leirs H, Portaels F (2009). A comparison of DNA extraction procedures for the detection of *Mycobacterium ulcerans*, the causative agent of Buruli ulcer, in clinical and environmental specimens. J Microbiol Methods.

[16] Portaels F, Agular J, Fissette K, Fonteyne PA, De Beenhouwer H, de Rijk P (1997). Direct detection and identification of *Mycobacterium ulcerans* in clinical specimens by PCR and oligonucleotide-specific capture plate hybridization. J Clin Microbiol.

[17] Roberts B, Hirst R (1997). Immunomagnetic separation and PCR for detection of *Mycobacterium ulcerans*. J Clin Microbiol.

[18] Fyfe JA, Lavender CJ, Johnson PD, Globan M, Sievers A, Azuolas J (2007). Development and application of two multiplex real-time PCR assays for the detection of *Mycobacterium ulcerans* in clinical and environmental samples. Appl Environ Microbiol.

[19] Ablordey A, Amissah DA, Aboagye IF, Hatano B, Yamazaki T, Sata T (2012). Detection of *Mycobacterium ulcerans* by the loop-mediated isothermal amplification method. PLoS Negl Trop Dis.

[20] Njiru ZK, Yeboah-Manu D, Stinear TP, Fyfe JA (2012). Rapid and sensitive detection of *Mycobacterium ulcerans* by use of a loop-mediated isothermal amplification test. J Clin Microbiol.

[21] Portaels F (2014). Laboratory diagnosis of Buruli ulcer.

[22] Ablordey A, Kotlowski R, Swings J, Portaels F (2005). PCR amplification with primers based on IS*2404* and GC-rich repeated sequence reveals polymorphism in *Mycobacterium ulcerans*. J Clin Microbiol.

[23] Edgar RC (2004). MUSCLE: multiple sequence alignment with high accuracy and high throughput. Nucleic Acids Res.

[24] Käser M, Rondini S, Naegeli M, Stinear T, Portaels F, Certa U (2007). Evolution of two distinct phylogenetic lineages of the emerging human pathogen *Mycobacterium ulcerans*. BMC Evol Biol.

[25] Qi W, Käser M, Roltgen K, Yeboah-Manu D, Pluschke G (2009). Genomic diversity and evolution of *Mycobacterium ulcerans* revealed by next-generation sequencing. PLoS Pathog.

