# Supplementary material for: Detection of Mycobacterium ulcerans by real-time PCR with improved primers
Source: Trop Med Health. 2016 Aug 19;44(1):28. doi: 10.1186/s41182-016-0028-3 (PMC5009631; doi:10.1186/s41182-016-0028-3)
Supplement: Additional file 1: Figure S1. — Sequence alignment of the IS2404 elements from the genome of M. ulcerans strain Agy99. (PDF 1040 kb) [file 41182_2016_28_MOESM1_ESM.pdf]

```
(query)MUL_0099.prj  1 ATGGCTTTGTTGGCGATCGCGGTTCTGGCCACTGCCGCGGGGATGCGCGGCTATGCTGGT 60
CP000325             1 ........A................................................... 60
CP000325             1 ............................................................ 60
CP000325             1 .................................A.......................... 60
CP000325             1 ............................................................ 60
CP000325             1 ..........................A................................. 60
CP000325             1 ............................................................ 60
CP000325             1 ............................................................ 60
CP000325             1 ............................................................ 60
CP000325             1 ............................................................ 60
CP000325             1 ............................................................ 60
CP000325             1 ............................................................ 60
CP000325             1 ....T......................................................C 60
CP000325             1 ............................................................ 60
CP000325             1 ............................................................ 60
CP000325             1 ............................................................ 60
CP000325             1 ............................................................ 60
CP000325             1 ............................................................ 60
CP000325             1 ............................................................ 60
CP000325             1 ............................................................ 60
CP000325             1 ............................................................ 60
CP000325             1 ............................................................ 60
CP000325             1 ...................................................C........ 60
CP000325             1 .......................................A.................... 60
CP000325             0 ------------------------------------------------------------ 0
CP000325             0 ------------------------------------------------------------ 0
CP000325             0 ------------------------------------------------------------ 0
CP000325             1 ............................................................ 60
CP000325             1 ............................................................ 60
CP000325             1 ................................C........................... 60
CP000325             1 ............................................................ 60
CP000325             1 ............................................................ 60
CP000325             1 ............................................................ 60
CP000325             1 ............................................................ 60
CP000325             1 ...................................................C........ 60
CP000325             1 ............................................................ 60
CP000325             1 ............................................................ 60
CP000325             1 ............................................................ 60
CP000325             1 ............................................................ 60
CP000325             1 ............................................................ 60
CP000325             1 ............................................................ 60
CP000325             1 ............................................................ 60
CP000325             1 ............................................................ 60
CP000325             1 ............................................................ 60
CP000325             1 ............................................................ 60
CP000325             1 ............................................................ 60
CP000325             1 ............................................................ 60
CP000325             1 ............................................................ 60
CP000325             1 ............................................................ 60
CP000325             1 ............................................................ 60
CP000325             1 ............................................................ 60
CP000325             1 ............................................................ 60
CP000325             1 ............................................................ 60
CP000325             1 ............................................................ 60
CP000325             1 ............................................................ 60
CP000325             1 ............................................................ 60
CP000325             1 ............................................................ 60
CP000325             1 ............................................................ 60
CP000325             1 ............................................................ 60
CP000325             1 ....T....................................................... 60
CP000325             1 ....T....................................................... 60
CP000325             1 ............................................................ 60
CP000325             1 .......................................A.................... 60
CP000325             1 ............................................................ 60
CP000325             1 ..........................A................................. 60
CP000325             1 .......................................A.................... 60
CP000325             1 ............................................................ 60
CP000325             1 ............................................................ 60
CP000325             1 ............................................................ 60
CP000325             1 ............................................................ 60
CP000325             1 .................................A.......................... 60
CP000325             1 ............................................................ 60
CP000325             1 ................................C........................... 60
CP000325             1 ............................................................ 60
CP000325             1 ...................................................C........ 60
CP000325             1 ............................................................ 60
CP000325             1 ............................................................ 60
```

```
CP000325 0 ------------------------------------------------------------ 0
CP000325 1 ............................................................ 60
CP000325 1 ............................................................ 60
CP000325 1 ............................................................ 60
CP000325 0 ------------------------------------------------------------ 0
CP000325 1 .......................................A.................... 60
CP000325 1 ..........................A................................. 60
CP000325 0 ------------------------------------------------------------ 0
CP000325 1 ............................................................ 60
CP000325 1 ............................................................ 60
CP000325 1 ............................................................ 60
CP000325 1 ............................................................ 60
CP000325 1 ............................................................ 60
CP000325 1 ............................................................ 60
CP000325 1 ............................................................ 60
CP000325 1 ............................................................ 60
CP000325 1 ............................................................ 60
CP000325 1 ............................................................ 60
CP000325 1 ............................................................ 60
CP000325 1 .......................................A.................... 60
CP000325 1 ............................................................ 60
CP000325 1 ............................................................ 60
CP000325 1 .......................................A.................... 60
CP000325 1 ............................................................ 60
CP000325 1 ............................................................ 60
CP000325 1 ............................................................ 60
CP000325 1 ............................................................ 60
CP000325 1 ............................................................ 60
CP000325 1 .......................................A.................... 60
CP000325 1 .......................................A.................... 60
CP000325 1 ............................................................ 60
CP000325 1 ............................................................ 60
CP000325 1 ............................................................ 60
CP000325 1 ............................................................ 60
CP000325 1 .......................................A.................... 60
CP000325 1 ............................................................ 60
CP000325 1 ............................................................ 60
CP000325 1 ..........................A................................. 60
CP000325 1 ............................................................ 60
CP000325 1 ............................................................ 60
CP000325 1 ............................................................ 60
CP000325 1 ..................................................T......... 60
CP000325 1 .................................A.......................... 60
CP000325 1 .......................................A.................... 60
CP000325 1 ............................................................ 60
CP000325 1 ............................................................ 60
CP000325 1 ............................................................ 60
CP000325 1 .......................................A.................... 60
CP000325 1 ............................................................ 60
CP000325 1 ............................................................ 60
CP000325 0 ------------------------------------------------------------ 0
CP000325 1 ............................................................ 60
CP000325 1 ............................................................ 60
CP000325 1 .................................A.......................... 60
CP000325 1 ............................................................ 60
CP000325 1 ............................................................ 60
CP000325 1 ...A........................................................ 60
CP000325 1 ..........................A................................. 60
CP000325 1 ............................................................ 60
CP000325 1 .................................A.......................... 60
CP000325 1 ............................................................ 60
CP000325 1 .......................................A.................... 60
CP000325 1 ..........................A................................. 60
CP000325 1 ..........................A................................. 60
CP000325 1 ............................................................ 60
CP000325 1 ............................................................ 60
CP000325 1 ............................................................ 60
CP000325 1 ............................................................ 60
CP000325 1 ............................................................ 60
CP000325 1 ..........................A................................. 60
CP000325 1 ............................................................ 60
CP000325 1 ............................................................ 60
CP000325 1 ............................................................ 60
CP000325 1 ............................................................ 60
CP000325 1 ............................................................ 60
CP000325 1 ................................C........................... 60
CP000325 1 ............................................................ 60
```

```
CP000325            1 ............................................................ 60
CP000325            1 ............................................................ 60
CP000325            1 ............................................................ 60
CP000325            1 .......................................A.................... 60
CP000325            1 ..........................A................................. 60
CP000325            1 ............................................................ 60
CP000325            1 .......................................A.................... 60
CP000325            1 .......................................A.................... 60
CP000325            1 ............................................................ 60
CP000325            1 ............................................................ 60
CP000325            1 ............................................................ 60
CP000325            1 ............................................................ 60
CP000325            1 ................................C........................... 60
CP000325            1 ..........................A................................. 60
CP000325            1 ............................................................ 60
CP000325            1 ............................................................ 60
CP000325            1 ............................................................ 60
CP000325            1 .......................................A.................... 60
CP000325            1 .................................A.......................... 60
CP000325            1 .......................................A.................... 60
CP000325            1 .......................................A.................... 60
CP000325            1 .......................................A.................... 60
CP000325            1 .......................................A.................... 60
CP000325            1 ............................................................ 60
CP000325            1 ............................................................ 60
CP000325            1 ............................................................ 60
CP000325            1 ............................................................ 60
CP000325            1 ............................................................ 60
CP000325            1 ............................................................ 60
CP000325            1 ............................................................ 60
CP000325            1 ............................................................ 60
CP000325            1 ...................................................C........ 60
CP000325            1 ............................................................ 60
CP000325            1 ............................................................ 60
CP000325            0 ------------------------------------------------------------ 0
CP000325            1 ............................................................ 60
CP000325            1 ............................................................ 60
CP000325            1 ............................................................ 60
CP000325            1 ............................................................ 60
CP000325            1 ............................................................ 60
CP000325            1 ............................................................ 60
CP000325            1 ............................................................ 60
CP000325            1 ............................................................ 60
CP000325            1 ............................................................ 60
CP000325            1 .......................................A.................... 60
CP000325            1 ............................................................ 60
CP000325            1 .......................................A.................... 60
CP000325            1 ............................................................ 60
CP000325            1 ............................................................ 60
CP000325            1 ............................................................ 60
CP000325            1 ............................................................ 60
CP000325            1 ............................................................ 60
CP000325            1 ............................................................ 60
CP000325            1 ............................................................ 60
CP000325            1 ............................................................ 60
CP000325            1 ............................................................ 60
CP000325            1 ............................................................ 60
CP000325            1 ............................................................ 60
CP000325            1 ............................................................ 60

(query)MUL_0099.prj  61 TTTGCCACATGGGCGGCCACCGCTTCCGATGATGTGTTGGCCCAATTAGGGGTCCGGTTC 120
CP000325            61 ............................................................ 120
CP000325            61 ........................................................A... 120
CP000325            61 ............................................................ 120
CP000325            61 ............................................................ 120
CP000325            61 .............T.............................................. 120
CP000325            61 .............................................G.............. 120
CP000325            61 ........................C....................G.............. 120
CP000325            61 .............................................G.............. 120
CP000325            61 .............................................G.............. 120
CP000325            61 .............................................G.............. 120
CP000325            61 .............................................G.............. 120
CP000325            61 ................T............................G.............. 120
CP000325            61 ............................................................ 120
CP000325            61 .............................................G.............. 120
CP000325            61 .............................................G.............. 120
CP000325            61 .............T...............................G.............. 120
```

```
CP000325    61 .............................................G.............. 120
CP000325    61 ............................................................ 120
CP000325    61 .............................................G.............. 120
CP000325    61 .............................................G.............. 120
CP000325    61 ................................................-........... 119
CP000325    61 ...........................................C....A...GT...... 120
CP000325    61 ........................C................................... 120
CP000325     0 ------------------------------------------------------------ 0
CP000325     1 --------------------------------------------------..A....... 10
CP000325     0 ------------------------------------------------------------ 0
CP000325    61 ...................................................C........ 120
CP000325    61 ............................................................ 120
CP000325    61 ............................................................ 120
CP000325    61 .............................................G.............. 120
CP000325    61 ............................................................ 120
CP000325    61 ............................................................ 120
CP000325    61 ............................................................ 120
CP000325    61 ................................................-........... 119
CP000325    61 .............................................G.............. 120
CP000325    61 ............................................................ 120
CP000325    61 ............................................................ 120
CP000325    61 ............................................................ 120
CP000325    61 ............................................................ 120
CP000325    61 ........................C................................... 120
CP000325    61 ............................................................ 120
CP000325    61 ............................................................ 120
CP000325    61 .............................................G.............. 120
CP000325    61 ............................................................ 120
CP000325    61 .............................................G.............. 120
CP000325    61 ............................................................ 120
CP000325    61 ............................................................ 120
CP000325    61 ..................................................T......... 120
CP000325    61 ..................................................T......... 120
CP000325    61 ............................................................ 120
CP000325    61 ............................................................ 120
CP000325    61 ............................................................ 120
CP000325    61 ............................................................ 120
CP000325    61 ............................................................ 120
CP000325    61 ............................................................ 120
CP000325    61 ............................................................ 120
CP000325    61 ............................................................ 120
CP000325    61 ............................................................ 120
CP000325    61 ................................................A........... 120
CP000325    61 ............................................................ 120
CP000325    61 .............................................G.............. 120
CP000325    61 .............................................G.............. 120
CP000325    61 ............................................................ 120
CP000325    61 ............................................................ 120
CP000325    61 ................................................-........... 119
CP000325    61 ............................................................ 120
CP000325    61 ............................................................ 120
CP000325    61 ............................................................ 120
CP000325    61 ............................................................ 120
CP000325    61 ............................................................ 120
CP000325    61 ............................................................ 120
CP000325    61 ............................................................ 120
CP000325    61 .............T.............................................. 120
CP000325    61 ................................................A........... 120
CP000325    61 ................................................-........... 119
CP000325    61 .............................................G.............. 120
CP000325     0 ------------------------------------------------------------ 0
CP000325    61 ............................................................ 120
CP000325    61 .............................................G.............. 120
CP000325    61 .............................................G.............. 120
CP000325     0 ------------------------------------------------------------ 0
CP000325    61 ............................................................ 120
CP000325    61 ............................................................ 120
CP000325     0 ------------------------------------------------------------ 0
CP000325    61 ............................................................ 120
CP000325    61 ............................................................ 120
CP000325    61 ............................................................ 120
CP000325    61 ............................................................ 120
CP000325    61 ........................C................................... 120
CP000325    61 ........................C................................... 120
CP000325    61 ............................................................ 120
CP000325    61 ............................................................ 120
CP000325    61 .............................................G.............. 120
```

```
CP000325                    61 .............................................G.............. 120
CP000325                    61 ............................................................ 120
CP000325                    61 ............................................................ 120
CP000325                    61 ............................................................ 120
CP000325                    61 ............................................................ 120
CP000325                    61 ............................................................ 120
CP000325                    61 .............................................G.............. 120
CP000325                    61 ............................................................ 120
CP000325                    61 ............................................................ 120
CP000325                    61 .............................................G.............. 120
CP000325                    61 .............................................G.............. 120
CP000325                    61 ................................................A........... 120
CP000325                    61 ................................................A........... 120
CP000325                    61 .............................................G.............. 120
CP000325                    61 .............................................G.............. 120
CP000325                    61 .............................................G.............. 120
CP000325                    61 ............................................................ 120
CP000325                    61 ................................................A........... 120
CP000325                    61 ............................................................ 120
CP000325                    61 ............................................................ 120
CP000325                    61 .............................................G.............. 120
CP000325                    61 ............................................................ 120
CP000325                    61 ............................................................ 120
CP000325                    61 ............................................................ 120
CP000325                    61 ............................................................ 120
CP000325                    61 ............................................................ 120
CP000325                    61 ........................C.......................A........... 120
CP000325                    61 ................................................-........... 119
CP000325                    61 ............................................................ 120
CP000325                    61 ............................................................ 120
CP000325                    61 ...........................................................T 120
CP000325                    61 ................................................-........... 119
CP000325                    61 ............................................................ 120
CP000325                    61 ............................................................ 120
CP000325                     0 ------------------------------------------------------------ 0
CP000325                    61 ................................................A........... 120
CP000325                    61 ................................................A........... 120
CP000325                    61 ................................................A........... 120
CP000325                    61 ...........................................................T 120
CP000325                    61 .............................................G.............. 120
CP000325                    61 ........................C................................... 120
CP000325                    61 ............................................................ 120
CP000325                    61 ............................................................ 120
CP000325                    61 ............................................................ 120
CP000325                    61 .............................................G.............. 120
CP000325                    61 .............................................G.............. 120
CP000325                    61 ............................................................ 120
CP000325                    61 .............T...............................G.............. 120
CP000325                    61 ............................................................ 120
CP000325                    61 .............................................G.............. 120
CP000325                    61 .............................................G.............. 120
CP000325                    61 ............................................................ 120
CP000325                    61 ............................................................ 120
CP000325                    61 ............................................................ 120
CP000325                    61 ............................................................ 120
CP000325                    61 ............................................................ 120
CP000325                    61 ............................................................ 120
CP000325                    61 ............................................................ 120
CP000325                    61 ............................................................ 120
CP000325                    61 ............................................................ 120
CP000325                    61 .............................................G.............. 120
CP000325                    61 ................................................A........... 120
CP000325                    61 ............................................................ 120
CP000325                    61 .............................................G.............. 120
CP000325                    61 .............................................G.............. 120
CP000325                    61 ............................................................ 120
CP000325                    61 ............................................................ 120
CP000325                    61 .............................................G.............. 120
CP000325                    61 .............................................G.............. 120
CP000325                    61 ............................................................ 120
CP000325                    61 ............................................................ 120
CP000325                    61 ............................................................ 120
CP000325                    61 ............................................................ 120
CP000325                    61 ............................................................ 120
CP000325                    61 ............................................................ 120
CP000325                    61 .............................................G.............. 120
CP000325                    61 .............T.............................................. 120
CP000325                    61 .............................................G.............. 120
```

```
CP000325             61 ............................................................ 120
CP000325             61 ................................................A........... 120
CP000325             61 ............................................................ 120
CP000325             61 ............................................................ 120
CP000325             61 ............................................................ 120
CP000325             61 ............................................................ 120
CP000325             61 .............................................C.............. 120
CP000325             61 ............................................................ 120
CP000325             61 ............................................................ 120
CP000325             61 ............................................................ 120
CP000325             61 ............................................................ 120
CP000325             61 ............................................................ 120
CP000325             61 ............................................................ 120
CP000325             61 ............................................................ 120
CP000325             61 ................................................A........... 120
CP000325             61 ........................C................................... 120
CP000325             61 ............................................................ 120
CP000325              0 ------------------------------------------------------------ 0
CP000325             61 ............................................................ 120
CP000325             61 .............T.............................................. 120
CP000325             61 .............................................G.............. 120
CP000325             61 .............T.............................................. 120
CP000325             61 ............................................................ 120
CP000325             61 ............................................................ 120
CP000325             61 ............................................................ 120
CP000325             61 .............................................G.............. 120
CP000325             61 .............................................G.............. 120
CP000325             61 ............................................................ 120
CP000325             61 .............................................G.............. 120
CP000325             61 ............................................................ 120
CP000325             61 ............................................................ 120
CP000325             61 ............................................................ 120
CP000325             61 ............................................................ 120
CP000325             61 ............................................................ 120
CP000325             61 ............................................................ 120
CP000325             61 ............................................................ 120
CP000325             61 ............................................................ 120
CP000325             61 ............................................................ 120
CP000325             61 ............................................................ 120
CP000325             61 ............................................................ 120
CP000325             61 ............................................................ 120
CP000325             61 ............................................................ 120

(query)MUL_0099.prj 121 CGGCGGCCCAGTGAGAAGACCTTCCGCGCTGTTTTGTCTCGGCTAGACCCCGCCGACCTC 180
CP000325            121 ..................................................---------- 170
CP000325            121 ............................................................ 180
CP000325            121 ............................................................ 180
CP000325            121 ...T........................................................ 180
CP000325            121 .....................................T...................... 180
CP000325            121 ............................................................ 180
CP000325            121 ............................................................ 180
CP000325            121 ............................................................ 180
CP000325            121 ............................................................ 180
CP000325            121 ............................................................ 180
CP000325            121 ...................T........................................ 180
CP000325            121 ............................................................ 180
CP000325            121 .....................................T...................... 180
CP000325            121 .....................................T...................... 180
CP000325            121 .....................................T...................... 180
CP000325            121 ............................................................ 180
CP000325            121 ............................................................ 180
CP000325            121 ............................................................ 180
CP000325            121 ............................................................ 180
CP000325            121 ............................................................ 180
CP000325            120 ............................................................ 179
CP000325            121 .C.......................................................... 180
CP000325            121 ............................................................ 180
CP000325              1 -----------------------------------------................... 19
CP000325             11 ........G..........................................--------- 61
CP000325              1 -----------------------------------------------............. 13
CP000325            121 ............................................................ 180
CP000325            121 ............................................................ 180
CP000325            121 ............................................................ 180
CP000325            121 ............................................................ 180
CP000325            121 ............................................................ 180
CP000325            121 ............................................................ 180
CP000325            121 ...................T........................................ 180
```

```
CP000325           120 ............................................................ 179
CP000325           121 .....................................T...................... 180
CP000325           121 ............................................................ 180
CP000325           121 ............................................................ 180
CP000325           121 ............................................................ 180
CP000325           121 ...................T........................................ 180
CP000325           121 ............................................................ 180
CP000325           121 .....................................T...................... 180
CP000325           121 .....................................T...................... 180
CP000325           121 ............................................................ 180
CP000325           121 ............................................................ 180
CP000325           121 ............................................................ 180
CP000325           121 ............................................................ 180
CP000325           121 ............................................................ 180
CP000325           121 ............................................................ 180
CP000325           121 ............................................................ 180
CP000325           121 ............................................................ 180
CP000325           121 ............................................................ 180
CP000325           121 ............................................................ 180
CP000325           121 ............................................................ 180
CP000325           121 ............................................................ 180
CP000325           121 ............................................................ 180
CP000325           121 ............................................................ 180
CP000325           121 ............................................................ 180
CP000325           121 ............................................................ 180
CP000325           121 ............................................................ 180
CP000325           121 ............................................................ 180
CP000325           121 ............................................................ 180
CP000325           121 ............................................................ 180
CP000325           121 ............................................................ 180
CP000325           121 ............................................................ 180
CP000325           121 ..........................................................CT 180
CP000325           120 ............................................................ 179
CP000325           121 ............................................................ 180
CP000325           121 ............................................................ 180
CP000325           121 ............................................................ 180
CP000325           121 ............................................................ 180
CP000325           121 ............................................................ 180
CP000325           121 ...................T........................................ 180
CP000325           121 ............................................................ 180
CP000325           121 .....................................T...................... 180
CP000325           121 ............................................................ 180
CP000325           120 ............................................................ 179
CP000325           121 ...................T........................................ 180
CP000325             0 ------------------------------------------------------------ 0
CP000325           121 .....................................T...................... 180
CP000325           121 ............................................................ 180
CP000325           121 ............................................................ 180
CP000325             0 ------------------------------------------------------------ 0
CP000325           121 ............................................................ 180
CP000325           121 .....................................T...................... 180
CP000325             0 ------------------------------------------------------------ 0
CP000325           121 ............................................................ 180
CP000325           121 ............................................................ 180
CP000325           121 ............................................................ 180
CP000325           121 ............................................................ 180
CP000325           121 ............................................................ 180
CP000325           121 ............................................................ 180
CP000325           121 ............................................................ 180
CP000325           121 ............................................................ 180
CP000325           121 ............................................................ 180
CP000325           121 ............................................................ 180
CP000325           121 ............................................................ 180
CP000325           121 ............................................................ 180
CP000325           121 ............................................................ 180
CP000325           121 ............................................................ 180
CP000325           121 ............................................................ 180
CP000325           121 ............................................................ 180
CP000325           121 ............................................................ 180
CP000325           121 ............................................................ 180
CP000325           121 ............................................................ 180
CP000325           121 ............................................................ 180
CP000325           121 ............................................................ 180
CP000325           121 ............................................................ 180
CP000325           121 ............................................................ 180
CP000325           121 ............................................................ 180
CP000325           121 .......................................................G.... 180
```

```
CP000325            121 ............................................................ 180
CP000325            121 ............................................................ 180
CP000325            121 ............................................................ 180
CP000325            121 ............................................................ 180
CP000325            121 ...................T........................................ 180
CP000325            121 ...........................................................T 180
CP000325            121 ............................................................ 180
CP000325            121 ............................................................ 180
CP000325            121 ............................................................ 180
CP000325            120 ............................................................ 179
CP000325            121 ............................................................ 180
CP000325            121 ............................................................ 180
CP000325            121 ............................................................ 180
CP000325            120 ............................................................ 179
CP000325            121 ............................................................ 180
CP000325            121 ............................................................ 180
CP000325              0 ------------------------------------------------------------ 0
CP000325            121 ............................................................ 180
CP000325            121 ............................................................ 180
CP000325            121 ............................................................ 180
CP000325            121 ............................................................ 180
CP000325            121 ............................................................ 180
CP000325            121 ............................................................ 180
CP000325            121 ...................T........................................ 180
CP000325            121 ............................................................ 180
CP000325            121 ............................................................ 180
CP000325            121 ............................................................ 180
CP000325            121 .....................................T...................... 180
CP000325            121 ...................T........................................ 180
CP000325            121 .....................................T...................... 180
CP000325            121 ............................................................ 180
CP000325            121 ............................................................ 180
CP000325            121 ............................................................ 180
CP000325            121 ............................................................ 180
CP000325            121 ............................................................ 180
CP000325            121 ............................................................ 180
CP000325            121 ............................................................ 180
CP000325            121 ............................................................ 180
CP000325            121 ............................................................ 180
CP000325            121 ............................................................ 180
CP000325            121 ............................................................ 180
CP000325            121 ............................................................ 180
CP000325            121 ............................................................ 180
CP000325            121 ............................................................ 180
CP000325            121 .....................................T...................... 180
CP000325            121 ............................................................ 180
CP000325            121 ............................................................ 180
CP000325            121 ............................................................ 180
CP000325            121 ............................................................ 180
CP000325            121 ............................................................ 180
CP000325            121 ............................................................ 180
CP000325            121 ............................................................ 180
CP000325            121 ............................................................ 180
CP000325            121 ............................................................ 180
CP000325            121 ............................................................ 180
CP000325            121 ............................................................ 180
CP000325            121 ............................................................ 180
CP000325            121 .....................................T...................... 180
CP000325            121 .....................................T...................... 180
CP000325            121 ............................................................ 180
CP000325            121 ............................................................ 180
CP000325            121 ............................................................ 180
CP000325            121 ............................................................ 180
CP000325            121 ............................................................ 180
CP000325            121 ............................................................ 180
CP000325            121 ............................................................ 180
CP000325            121 .....................................T...................... 180
CP000325            121 ............................................................ 180
CP000325            121 ............................................................ 180
CP000325            121 ............................................................ 180
CP000325            121 ............................................................ 180
CP000325            121 ............................................................ 180
CP000325            121 ............................................................ 180
CP000325            121 .....................................T...................... 180
CP000325            121 ............................................................ 180
CP000325            121 ............................................................ 180
CP000325            121 ............................................................ 180
```

```
CP000325               0 ------------------------------------------------------------ 0
CP000325             121 ............................................................ 180
CP000325             121 ............................................................ 180
CP000325             121 ............................................................ 180
CP000325             121 ............................................................ 180
CP000325             121 ............................................................ 180
CP000325             121 ............................................................ 180
CP000325             121 ............................................................ 180
CP000325             121 ............................................................ 180
CP000325             121 ............................................................ 180
CP000325             121 ............................................................ 180
CP000325             121 ............................................................ 180
CP000325             121 ............................................................ 180
CP000325             121 ............................................................ 180
CP000325             121 ............................................................ 180
CP000325             121 ............................................................ 180
CP000325             121 ............................................................ 180
CP000325             121 ............................................................ 180
CP000325             121 ............................................................ 180
CP000325             121 ............................................................ 180
CP000325             121 ............................................................ 180
CP000325             121 ............................................................ 180
CP000325             121 ............................................................ 180
CP000325             121 ............................................................ 180
CP000325             121 ............................................................ 180

(query)MUL_0099.prj  181 AACGCCAGGATGGGCAGTTACTTCACTGCACACGTGGCCAGCAGCGACCCCAGTGGATTG 240
CP000325             170 ------------------------------------------------------------ 170
CP000325             181 ...................................................G........ 240
CP000325             181 ...................................................G........ 240
CP000325             181 ............................................................ 240
CP000325             181 ............................................................ 240
CP000325             181 ............................................................ 240
CP000325             181 ............................................................ 240
CP000325             181 ............................................................ 240
CP000325             181 ............................................................ 240
CP000325             181 ...................................................G........ 240
CP000325             181 ............................................................ 240
CP000325             181 ............................................................ 240
CP000325             181 ............................................................ 240
CP000325             181 ............................................................ 240
CP000325             181 ............................................................ 240
CP000325             181 ............................................................ 240
CP000325             181 ............................................................ 240
CP000325             181 ...................................................G........ 240
CP000325             181 ....................T..............................G........ 240
CP000325             181 ....................T..............................G........ 240
CP000325             180 ............................................................ 239
CP000325             181 ............................................................ 240
CP000325             181 .............----------------------------------------------- 193
CP000325              20 ............................................................ 79
CP000325              61 ------------------------------------------------------------ 61
CP000325              14 ....................T....................................... 73
CP000325             181 .............A.............................................. 240
CP000325             181 ............................................................ 240
CP000325             181 ............................................................ 240
CP000325             181 ............................................................ 240
CP000325             181 ............................................................ 240
CP000325             181 ............................................................ 240
CP000325             181 ............................................................ 240
CP000325             180 ............................................................ 239
CP000325             181 ...................................................G........ 240
CP000325             181 ............................................................ 240
CP000325             181 ............................................................ 240
CP000325             181 ...................................................G........ 240
CP000325             181 ............................................................ 240
CP000325             181 ............................................................ 240
CP000325             181 ............................................................ 240
CP000325             181 ............................................................ 240
CP000325             181 ....................T..............................G........ 240
CP000325             181 ............................................................ 240
CP000325             181 ............................................................ 240
CP000325             181 ............................................................ 240
CP000325             181 ............................................................ 240
CP000325             181 ............................................................ 240
CP000325             181 ............................................................ 240
CP000325             181 ............................................................ 240
```

```
CP000325      181 ...............................................T............ 240
CP000325      181 ............................................................ 240
CP000325      181 ............................................................ 240
CP000325      181 ............................................................ 240
CP000325      181 ............................................................ 240
CP000325      181 ............................................................ 240
CP000325      181 ............................................................ 240
CP000325      181 ............................................................ 240
CP000325      181 ............................................................ 240
CP000325      181 ............................................................ 240
CP000325      181 ............................................................ 240
CP000325      181 ............................................................ 240
CP000325      181 ............................................................ 240
CP000325      181 ............................................................ 240
CP000325      180 ............................................................ 239
CP000325      181 ...............G............................................ 240
CP000325      181 ...................................................G........ 240
CP000325      181 ............................................................ 240
CP000325      181 ............................................................ 240
CP000325      181 ............................................................ 240
CP000325      181 ............................................................ 240
CP000325      181 ............................................................ 240
CP000325      181 ............................................................ 240
CP000325      181 ............................................................ 240
CP000325      180 ............................................................ 239
CP000325      181 ...................................................G........ 240
CP000325        0 ------------------------------------------------------------ 0
CP000325      181 ............................................................ 240
CP000325      181 ............................................................ 240
CP000325      181 ............................................................ 240
CP000325        0 ------------------------------------------------------------ 0
CP000325      181 ............................................................ 240
CP000325      181 .............A.............................................. 240
CP000325        0 ------------------------------------------------------------ 0
CP000325      181 ............................................................ 240
CP000325      181 ............................................................ 240
CP000325      181 ............................................................ 240
CP000325      181 ............................................................ 240
CP000325      181 ............................................................ 240
CP000325      181 ............................................................ 240
CP000325      181 ............................................................ 240
CP000325      181 ............................................................ 240
CP000325      181 ............................................................ 240
CP000325      181 ............................................................ 240
CP000325      181 ............................................................ 240
CP000325      181 ....................T..............................G........ 240
CP000325      181 ............................................................ 240
CP000325      181 ............................................................ 240
CP000325      181 ............................................................ 240
CP000325      181 ...................................................G........ 240
CP000325      181 ............................................................ 240
CP000325      181 ...................................................G........ 240
CP000325      181 ............................................................ 240
CP000325      181 ............................................................ 240
CP000325      181 ...................................................G........ 240
CP000325      181 ............................................................ 240
CP000325      181 ............................................................ 240
CP000325      181 ............................................................ 240
CP000325      181 ............................................................ 240
CP000325      181 ............................................................ 240
CP000325      181 ............................................................ 240
CP000325      181 ...................................................G........ 240
CP000325      181 ............................................................ 240
CP000325      181 ............................................................ 240
CP000325      181 ............................................................ 240
CP000325      181 C........................................................... 240
CP000325      181 ............................................................ 240
CP000325      181 ............................................................ 240
CP000325      181 ............................................................ 240
CP000325      180 ............................................................ 239
CP000325      181 ............................................................ 240
CP000325      181 ............................................................ 240
CP000325      181 ............................................................ 240
CP000325      180 .........................................T.................. 239
CP000325      181 ...................................................G........ 240
CP000325      181 ............................................................ 240
CP000325        0 ------------------------------------------------------------ 0
```

```
CP000325        181 .........G.................................................. 240
CP000325        181 ............................................................ 240
CP000325        181 ............................................................ 240
CP000325        181 ............................................................ 240
CP000325        181 ............................................................ 240
CP000325        181 ............................................................ 240
CP000325        181 ............................................................ 240
CP000325        181 ............A............................................... 240
CP000325        181 ............................................................ 240
CP000325        181 ............................................................ 240
CP000325        181 ............................................................ 240
CP000325        181 ............................................................ 240
CP000325        181 ............................................................ 240
CP000325        181 ..........................C................................. 240
CP000325        181 ..........................C................................. 240
CP000325        181 ..........................C................................. 240
CP000325        181 ............................................................ 240
CP000325        181 ............................................................ 240
CP000325        181 ...............................................T............ 240
CP000325        181 ...............................................T............ 240
CP000325        181 ............................................................ 240
CP000325        181 ............................................................ 240
CP000325        181 .............A.............................................. 240
CP000325        181 ............................................................ 240
CP000325        181 ............................................................ 240
CP000325        181 ............................................................ 240
CP000325        181 ............................................................ 240
CP000325        181 ............................................................ 240
CP000325        181 ............................................................ 240
CP000325        181 .............A.............................................. 240
CP000325        181 ............................................................ 240
CP000325        181 ............................................................ 240
CP000325        181 ............................................................ 240
CP000325        181 ............................................................ 240
CP000325        181 ............................................................ 240
CP000325        181 ............................................................ 240
CP000325        181 ............................................................ 240
CP000325        181 ............................................................ 240
CP000325        181 ............................................................ 240
CP000325        181 ............................................................ 240
CP000325        181 ............................................................ 240
CP000325        181 ............................................................ 240
CP000325        181 ............................................................ 240
CP000325        181 ............................................................ 240
CP000325        181 ............................................................ 240
CP000325        181 ............................................................ 240
CP000325        181 .............A.............................................. 240
CP000325        181 ............................................................ 240
CP000325        181 ............................................................ 240
CP000325        181 ...................................................G........ 240
CP000325        181 .............A.............................................. 240
CP000325        181 .............A.............................................. 240
CP000325        181 ............................................................ 240
CP000325        181 ...................................................G........ 240
CP000325        181 ...................................................G........ 240
CP000325        181 .........................................................C.. 240
CP000325        181 ............................................................ 240
CP000325        181 ............................................................ 240
CP000325        181 ............................................................ 240
CP000325        181 ............................................................ 240
CP000325          0 ------------------------------------------------------------ 0
CP000325        181 ............................................................ 240
CP000325        181 ............................................................ 240
CP000325        181 ............................................................ 240
CP000325        181 ............................................................ 240
CP000325        181 ............................................................ 240
CP000325        181 ............................................................ 240
CP000325        181 ............................................................ 240
CP000325        181 ............................................................ 240
CP000325        181 ............................................................ 240
CP000325        181 ............................................................ 240
CP000325        181 ............................................................ 240
CP000325        181 ............................................................ 240
CP000325        181 ............................................................ 240
CP000325        181 ............................................................ 240
CP000325        181 ............................................................ 240
CP000325        181 ............................................................ 240
```

```
CP000325             181 ............................................................ 240
CP000325             181 ...................................................G........ 240
CP000325             181 ....................T..............................G........ 240
CP000325             181 ............................................................ 240
CP000325             181 ............................................................ 240
CP000325             181 ............................................................ 240
CP000325             181 ............................................................ 240
CP000325             181 .............C...------------------------------------------- 197

(query)MUL_0099.prj  241 --GTGCCGATCGCGTTGGACGGCAAGATGCTGCGTGGTGCTTTACGCGCCAAAGCGACAG 298
CP000325             170 ------------------------------------------------------------ 170
CP000325             241 --.......................................................... 298
CP000325             241 --.......................................................... 298
CP000325             241 --.......................................................... 298
CP000325             241 --.......................................................... 298
CP000325             241 GT.................................A........................ 300
CP000325             241 --.......................................................... 298
CP000325             241 --.......................................................... 298
CP000325             241 --.......................................................... 298
CP000325             241 --.......................................................... 298
CP000325             241 --.......................................................... 298
CP000325             241 --.......................................................... 298
CP000325             241 --.............................................A............ 298
CP000325             241 --.......................................................... 298
CP000325             241 --.......................................................... 298
CP000325             241 --.......................................................... 298
CP000325             241 --.......................................................... 298
CP000325             241 --.......................................................... 298
CP000325             241 --.......................................................... 298
CP000325             241 --.......................................................... 298
CP000325             240 --.......................................................... 297
CP000325             241 --.......................................................... 298
CP000325             193 ------------------------------------------------------------ 193
CP000325              80 --.......................................................... 137
CP000325              61 ------------------------------------------------------------ 61
CP000325              74 --.......................................................... 131
CP000325             241 --.......................................................... 298
CP000325             241 --.......................................................... 298
CP000325             241 --.......................................................... 298
CP000325             241 --.......................................................... 298
CP000325             241 --.......................................................... 298
CP000325             241 --...............................................T.......... 298
CP000325             241 --.......................................................... 298
CP000325             240 --.......................................................... 297
CP000325             241 --.......................................................... 298
CP000325             241 --.......................................................... 298
CP000325             241 --...............................................T.......... 298
CP000325             241 --.......................................................... 298
CP000325             241 --.......................................................... 298
CP000325             241 --.......................................................... 298
CP000325             241 --.......................................................... 298
CP000325             241 --.......................................................... 298
CP000325             241 --.......................................................... 298
CP000325             241 --.......................................................... 298
CP000325             241 --.......................................................... 298
CP000325             241 --.......................................................... 298
CP000325             241 --.......................................................... 298
CP000325             241 --.......................................................... 298
CP000325             241 --.......................................................... 298
CP000325             241 --.......................................................... 298
CP000325             241 --.......................................................... 298
CP000325             241 --.......................................................... 298
CP000325             241 --.......................................................... 298
CP000325             241 --.......................................................... 298
CP000325             241 --.......................................................... 298
CP000325             241 --.......................................................... 298
CP000325             241 --.......................................................... 298
CP000325             241 --............................................T............. 298
CP000325             241 --.......................................................... 298
CP000325             241 --.......................................................... 298
CP000325             241 --.......................................................... 298
CP000325             241 --.......................................................... 298
CP000325             241 --.......................................................... 298
CP000325             241 --.......................................................... 298
CP000325             240 --.......................................................... 297
CP000325             241 --.......................................................... 298
CP000325             241 --.......................................................... 298
```

```
CP000325        241 --........................................................ 298
CP000325        241 --........................................................ 298
CP000325        241 --........................................................ 298
CP000325        241 --........................................................ 298
CP000325        241 --........................................................ 298
CP000325        241 --........................................................ 298
CP000325        241 --........................................................ 298
CP000325        240 --........................................................ 297
CP000325        241 --........................................................ 298
CP000325          0 ---------------------------------------------------------- 0
CP000325        241 --........................................................ 298
CP000325        241 --........................................................ 298
CP000325        241 --...................................A.................... 298
CP000325          0 ---------------------------------------------------------- 0
CP000325        241 --........................................................ 298
CP000325        241 --........................................................ 298
CP000325          0 ---------------------------------------------------------- 0
CP000325        241 --.......................G................................ 298
CP000325        241 --........................................................ 298
CP000325        241 --........................................................ 298
CP000325        241 --........................................................ 298
CP000325        241 --........................................................ 298
CP000325        241 --........................................................ 298
CP000325        241 --........................................................ 298
CP000325        241 --........................................................ 298
CP000325        241 --...............................C........................ 298
CP000325        241 --........................................................ 298
CP000325        241 --........................................................ 298
CP000325        241 --........................................................ 298
CP000325        241 --........................................................ 298
CP000325        241 --........................................................ 298
CP000325        241 --........................................................ 298
CP000325        241 --........................................................ 298
CP000325        241 --........................................................ 298
CP000325        241 --........................................................ 298
CP000325        241 --........................................................ 298
CP000325        241 --........................................................ 298
CP000325        241 --........................................................ 298
CP000325        241 --........................................................ 298
CP000325        241 --........................................................ 298
CP000325        241 --........................................................ 298
CP000325        241 --........................................................ 298
CP000325        241 --........................................................ 298
CP000325        241 --........................................................ 298
CP000325        241 --........................................................ 298
CP000325        241 --........................................................ 298
CP000325        241 --........................................................ 298
CP000325        241 --........................................................ 298
CP000325        241 --........................................................ 298
CP000325        241 --........................................................ 298
CP000325        241 --........................................................ 298
CP000325        241 --........................................................ 298
CP000325        240 --........................................................ 297
CP000325        241 --........................................................ 298
CP000325        241 --........................................................ 298
CP000325        241 --........................................................ 298
CP000325        240 --........................................................ 297
CP000325        241 --........................................................ 298
CP000325        241 --........................................................ 298
CP000325          0 ---------------------------------------------------------- 0
CP000325        241 --........................................................ 298
CP000325        241 --........................................................ 298
CP000325        241 --........................................................ 298
CP000325        241 --........................................................ 298
CP000325        241 --........................................................ 298
CP000325        241 --........................................................ 298
CP000325        241 --........................................................ 298
CP000325        241 --........................................................ 298
CP000325        241 --........................................................ 298
CP000325        241 --........................................................ 298
CP000325        241 --........................................................ 298
CP000325        241 --........................................................ 298
CP000325        241 --...................................A.................... 298
CP000325        241 --........................................................ 298
CP000325        241 --........................................................ 298
CP000325        241 --........................................................ 298
CP000325        241 --........................................................ 298
```

```
CP000325             241 --.......................................................... 298
CP000325             241 --.......................................................... 298
CP000325             241 --.......................................................... 298
CP000325             241 --.......................................................... 298
CP000325             241 --.......................................................... 298
CP000325             241 --.......................................................... 298
CP000325             241 --.......................................................... 298
CP000325             241 --.......................................................... 298
CP000325             241 --.......................................................... 298
CP000325             241 --.......................................................... 298
CP000325             241 --.......................................................... 298
CP000325             241 GT.................................A........................ 300
CP000325             241 --.......................................................... 298
CP000325             241 --.......................................................... 298
CP000325             241 --.......................................................... 298
CP000325             241 --.......................................................... 298
CP000325             241 --.......................................................... 298
CP000325             241 --.......................................................... 298
CP000325             241 --.......................................................... 298
CP000325             241 --.......................................................... 298
CP000325             241 --.......................................................... 298
CP000325             241 --.......................................................... 298
CP000325             241 --.......................................................... 298
CP000325             241 --.......................................................... 298
CP000325             241 --.......................................................... 298
CP000325             241 --.......................................................... 298
CP000325             241 --......................G................................... 298
CP000325             241 --.......................................................... 298
CP000325             241 --.......................................................... 298
CP000325             241 --.......................................................... 298
CP000325             241 --.......................................................... 298
CP000325             241 --.......................................................... 298
CP000325             241 --.......................................................... 298
CP000325             241 --.......................................................... 298
CP000325             241 --.......................................................... 298
CP000325             241 --.......................................................... 298
CP000325             241 --.........................G................................ 298
CP000325             241 --.........................G................................ 298
CP000325             241 --.......................................................... 298
CP000325             241 --.......................................................... 298
CP000325             241 --.......................................................... 298
CP000325             241 --.......................................................... 298
CP000325             241 --.......................................................... 298
CP000325               0 ------------------------------------------------------------ 0
CP000325             241 --.......................................................... 298
CP000325             241 --.......................................................... 298
CP000325             241 --.......................................................... 298
CP000325             241 --.......................................................... 298
CP000325             241 --.......................................................... 298
CP000325             241 --.......................................................... 298
CP000325             241 --.......................................................... 298
CP000325             241 --.......................................................... 298
CP000325             241 --.......................................................... 298
CP000325             241 --.......................................................... 298
CP000325             241 --.......................................................... 298
CP000325             241 --.......................................................... 298
CP000325             241 --.......................................................... 298
CP000325             241 --.......................................................... 298
CP000325             241 --.......................................................... 298
CP000325             241 --.......................................................... 298
CP000325             241 --.......................................................... 298
CP000325             241 --.......................................................... 298
CP000325             241 --.......................................................... 298
CP000325             241 --.......................................................... 298
CP000325             241 --.......................................................... 298
CP000325             241 --.......................................................... 298
CP000325             241 --.......................................................... 298
CP000325             197 ------------------------------------------------------------ 197

(query)MUL_0099.prj  299 CCACGCATCTCGTGTCGGTGTTCGCCTACCGTGCCCGATTGGTGCTCGGTCAACTCGCTG 358
CP000325             170 ------------------------------------------------------------ 170
CP000325             299 ..........................C................................. 358
CP000325             299 ..........................C................................. 358
CP000325             299 ..........................C................................. 358
CP000325             299 ..........................C................................. 358
CP000325             301 ..........................C................................. 360
CP000325             299 ..........................C..........................G...... 358
```

```
CP000325                299 ..........................C................................. 358
CP000325                299 ..........................C................................. 358
CP000325                299 ..........................C.......................T......... 358
CP000325                299 ..........................C................................. 358
CP000325                299 ..........................C................................. 358
CP000325                299 ..........................C................................. 358
CP000325                299 ..........................C................................. 358
CP000325                299 ..........................C................................. 358
CP000325                299 ..........................C................................. 358
CP000325                299 ..........................C................................. 358
CP000325                299 ..........................C................................. 358
CP000325                299 ..........................C................................. 358
CP000325                299 ..........................C................................. 358
CP000325                298 ..........................C................................. 357
CP000325                299 ..........................C................................. 358
CP000325                193 ------------------------------------------------------------ 193
CP000325                138 ..........................C................................. 197
CP000325                 61 ------------------------------------------------------------ 61
CP000325                132 ..........................C................................. 191
CP000325                299 ..........................C................................. 358
CP000325                299 ..........................C................................. 358
CP000325                299 ..........................C................................. 358
CP000325                299 ..........................C................................. 358
CP000325                299 ..........................C................................. 358
CP000325                299 ..........................C................................. 358
CP000325                299 ..........................C................................. 358
CP000325                298 ..........................C..................C.............. 357
CP000325                299 ..........................C................................. 358
CP000325                299 ..........................C................................. 358
CP000325                299 ..........................C................................. 358
CP000325                299 ..........................C................................. 358
CP000325                299 ..........................C................................. 358
CP000325                299 ..........................C................................. 358
CP000325                299 ..........................C................................. 358
CP000325                299 ..G.......................C................................. 358
CP000325                299 ..........................C................................. 358
CP000325                299 ..........................C................................. 358
CP000325                299 ..........................C........................G........ 358
CP000325                299 ..........................C................................. 358
CP000325                299 ..........................C................................. 358
CP000325                299 ..........................C................................. 358
CP000325                299 ..........................C................................. 358
CP000325                299 ..........................C................................. 358
CP000325                299 ..........................C................................. 358
CP000325                299 ..........................C................................. 358
CP000325                299 ..........................C................................. 358
CP000325                299 ..........................C................................. 358
CP000325                299 ..........................C................................. 358
CP000325                299 ..........................C................................. 358
CP000325                299 ..........................C................................. 358
CP000325                299 ..........................C.............A................... 358
CP000325                299 ..........................C................................. 358
CP000325                299 ..........................C................................. 358
CP000325                299 ..........................C................................. 358
CP000325                299 ..........................C................................. 358
CP000325                299 ..........................C................................. 358
CP000325                299 ..........................C................................. 358
CP000325                298 ..........................C................................. 357
CP000325                299 ..........................C................................. 358
CP000325                299 ..........................C................................. 358
CP000325                299 ..........................C................................. 358
CP000325                299 ..........................C................................. 358
CP000325                299 ..........................C................................. 358
CP000325                299 ..........................C................................. 358
CP000325                299 ..........................C............C.................... 358
CP000325                299 ..........................C................................. 358
CP000325                299 ..........................C................................. 358
CP000325                298 ..........................C................................. 357
CP000325                299 ..........................C................................. 358
CP000325                  0 ------------------------------------------------------------ 0
CP000325                299 ..........................C................................. 358
CP000325                299 ..........................C................................. 358
CP000325                299 ..........................C................................. 358
CP000325                  0 ------------------------------------------------------------ 0
CP000325                299 ..........................C..................C.............. 358
CP000325                299 ..........................C................................. 358
CP000325                  0 ------------------------------------------------------------ 0
```

```
CP000325            299 ............................................................ 358
CP000325            299 ..........................C................................. 358
CP000325            299 ..........................C................................. 358
CP000325            299 ..........................C................................. 358
CP000325            299 ..........................C................................. 358
CP000325            299 ..........................C................................. 358
CP000325            299 ..........................C................................. 358
CP000325            299 ..........................C................................. 358
CP000325            299 ..........................C................................. 358
CP000325            299 ..........................C................................. 358
CP000325            299 ............................................................ 358
CP000325            299 ..........................C................................. 358
CP000325            299 ..........................C................................. 358
CP000325            299 ..........................C................................. 358
CP000325            299 ..........................C................................. 358
CP000325            299 ..........................C................................. 358
CP000325            299 ..........................C................................. 358
CP000325            299 ..........................C................................. 358
CP000325            299 ..........................C................................. 358
CP000325            299 ..........................C................................. 358
CP000325            299 ..........................C................................. 358
CP000325            299 ..........................C................................. 358
CP000325            299 ..........................C................................. 358
CP000325            299 ..........................C................................. 358
CP000325            299 ..........................C................................. 358
CP000325            299 ..........................C..................C.............. 358
CP000325            299 ..........................C................................. 358
CP000325            299 ..........................C................................. 358
CP000325            299 ..........................C................................. 358
CP000325            299 ..........................C............................T.... 358
CP000325            299 ..........................C................................. 358
CP000325            299 ..........................C................................. 358
CP000325            299 ..........................C................................. 358
CP000325            299 ..........................C............C.................... 358
CP000325            299 ..........................C......T.......................... 358
CP000325            298 ..........................C..................C.............. 357
CP000325            299 ..........................C................................. 358
CP000325            299 ..........................C..................C.............. 358
CP000325            299 .......................A..C................................. 358
CP000325            298 .......................A..C..................C.............. 357
CP000325            299 ..........................C................................. 358
CP000325            299 ..........................C................................. 358
CP000325              0 ------------------------------------------------------------ 0
CP000325            299 ..........................C................................. 358
CP000325            299 ..........................C................................. 358
CP000325            299 ..........................C................................. 358
CP000325            299 ..........................C................................. 358
CP000325            299 ..........................C................................. 358
CP000325            299 ..........................C................................. 358
CP000325            299 ..........................C................................. 358
CP000325            299 ..........................C....C............................ 358
CP000325            299 ..........................C................................. 358
CP000325            299 ..........................C................................. 358
CP000325            299 ..........................C................................. 358
CP000325            299 ..........................C................................. 358
CP000325            299 ..........................C................................. 358
CP000325            299 ..........................C..................C.............. 358
CP000325            299 ..........................C................................. 358
CP000325            299 ..........................C..................C.............. 358
CP000325            299 ..........................C................................. 358
CP000325            299 ..........................C................................. 358
CP000325            299 ..........................C................................. 358
CP000325            299 ..........................C................................. 358
CP000325            299 .......................A..C................................. 358
CP000325            299 .......................A..C................................. 358
CP000325            299 ..........................C................................. 358
CP000325            299 ..........................C................................. 358
CP000325            299 ..........................C................................. 358
CP000325            299 ..........................C................................. 358
CP000325            299 ..........................C................................. 358
CP000325            299 ..........................C................................. 358
CP000325            301 ..........................C................................. 360
CP000325            299 ..........................C................................. 358
CP000325            299 ..........................C................................. 358
CP000325            299 ..........................C................................. 358
CP000325            299 ..........................C................................. 358
CP000325            299 ..........................C................................. 358
```

```
CP000325            299 ..........................C................................. 358
CP000325            299 ..........................C................................. 358
CP000325            299 .......C..................C................................. 358
CP000325            299 .......C..................C................................. 358
CP000325            299 ..........................C........................G........ 358
CP000325            299 ..........................C................................. 358
CP000325            299 ..........................C................................. 358
CP000325            299 ..........................C................................. 358
CP000325            299 ..........................C................................. 358
CP000325            299 ..........................C................................. 358
CP000325            299 ..........................C................................. 358
CP000325            299 ..........................C................................. 358
CP000325            299 ..........................C................................. 358
CP000325            299 ..........................C................................. 358
CP000325            299 ..........................C................................. 358
CP000325            299 ..........................C................................. 358
CP000325            299 ..........................C.......................T......... 358
CP000325            299 ..........................C................................. 358
CP000325            299 .......................A..C................................. 358
CP000325            299 ..........................C................................. 358
CP000325            299 ..........................C................................. 358
CP000325            299 ..........................C................................. 358
CP000325            299 ..........................C................................. 358
CP000325            299 ..........................C................................. 358
CP000325            299 ..........................C................................. 358
CP000325            299 .......................A..C................................. 358
CP000325              1 --------------------------------............................ 28
CP000325            299 ..........................C................................. 358
CP000325            299 ..........................C................................. 358
CP000325            299 ..........................C................................. 358
CP000325            299 ..........................C................................. 358
CP000325            299 ..........................C........................G........ 358
CP000325            299 ..........................C................................. 358
CP000325            299 ..........................C................................. 358
CP000325            299 ..........................C................................. 358
CP000325            299 ..........................C................................. 358
CP000325            299 ..........................C................................. 358
CP000325            299 ..........................C................................. 358
CP000325            299 ..........................C................................. 358
CP000325            299 ..........................C................................. 358
CP000325            299 ..........................C................................. 358
CP000325            299 ..........................C..................C.............. 358
CP000325            299 ..........................C................................. 358
CP000325            299 ..........................C......T.......................... 358
CP000325            299 ..........................C.................T............... 358
CP000325            299 ..........................C................................. 358
CP000325            299 ..........................C..................C.............. 358
CP000325            299 ..........................C................................. 358
CP000325            299 ..........................C................................. 358
CP000325            299 ..........................C........................G........ 358
CP000325            197 ------------------------------------------------------------ 197

(query)MUL_0099.prj 359 TCGCCGAGAAAAGCAATGAAATTCCCTGCGTACGTGCCCTGCTCACGCTGCTGCCGGGTA 418
CP000325            170 ------------------------------------------------------------ 170
CP000325            359 ................................T........................A.. 418
CP000325            359 ................................T........................A.. 418
CP000325            359 ............................................................ 418
CP000325            359 ............................................................ 418
CP000325            361 .........................................T...............A.. 420
CP000325            359 .........................................................A.. 418
CP000325            359 .......................................................A.A.. 418
CP000325            359 ............................................................ 418
CP000325            359 ................................T........................... 418
CP000325            359 .........................................................A.. 418
CP000325            359 ............................................................ 418
CP000325            359 ............................................................ 418
CP000325            359 ................................T........................... 418
CP000325            359 .........................................................A.. 418
CP000325            359 ................................T........................... 418
CP000325            359 ................................T........................... 418
CP000325            359 ................................T........................A.. 418
CP000325            359 ................................T........................A.. 418
CP000325            359 ................................T........................A.. 418
CP000325            358 .........................................................A.. 417
CP000325            359 .........................................................A.. 418
CP000325            193 ------------------------------------------------------------ 193
CP000325            198 .........................................................A.. 257
```

```
CP000325        61 ------------------------------------------------------------ 61
CP000325       192 ................................T........................A.. 251
CP000325       359 .........................................................A.. 418
CP000325       359 .........................................................A.. 418
CP000325       359 .........................................................A.. 418
CP000325       359 ................................T........................A.. 418
CP000325       359 .........................................................A.. 418
CP000325       359 .........................................................A.. 418
CP000325       359 ................................T........................A.. 418
CP000325       358 ............A............................................... 417
CP000325       359 .........................................................A.. 418
CP000325       359 .........................................................A.. 418
CP000325       359 .....................................................T...... 418
CP000325       359 .........................................................A.. 418
CP000325       359 ................................T........................A.. 418
CP000325       359 .........................................................A.. 418
CP000325       359 ................................T........................A.. 418
CP000325       359 ................................T........................... 418
CP000325       359 ................................T........................A.. 418
CP000325       359 ................................T........................A.. 418
CP000325       359 ............................................................ 418
CP000325       359 .........................................................A.. 418
CP000325       359 ............A............................................A.. 418
CP000325       359 ............................................................ 418
CP000325       359 ............................................................ 418
CP000325       359 ............................................................ 418
CP000325       359 ............................................................ 418
CP000325       359 .........................................................A.. 418
CP000325       359 ................................T........................A.. 418
CP000325       359 ................................T........................... 418
CP000325       359 ............................................................ 418
CP000325       359 ............................................................ 418
CP000325       359 ................................T........................A.. 418
CP000325       359 ................................T........................A.. 418
CP000325       359 .........................................................A.. 418
CP000325       359 ................................T........................A.. 418
CP000325       359 .........................................................A.. 418
CP000325       359 ................................T........................A.. 418
CP000325       359 ................................T........G...............A.. 418
CP000325       359 ................................T........................... 418
CP000325       358 ................................T........................A.. 417
CP000325       359 .........................................................A.. 418
CP000325       359 ............................................................ 418
CP000325       359 ................................T........G...............A.. 418
CP000325       359 .........................................................A.. 418
CP000325       359 .........................................................A.. 418
CP000325       359 ................................T........................A.. 418
CP000325       359 ................................T........................A.. 418
CP000325       359 ................................T........................... 418
CP000325       359 .........................................................A.. 418
CP000325       358 ................................T........................A.. 417
CP000325       359 ............................................................ 418
CP000325         0 ------------------------------------------------------------ 0
CP000325       359 ............................................................ 418
CP000325       359 ............................................................ 418
CP000325       359 .........................................................A.. 418
CP000325         0 ------------------------------------------------------------ 0
CP000325       359 ............A............................................A.. 418
CP000325       359 .........................................................A.. 418
CP000325         0 ------------------------------------------------------------ 0
CP000325       359 .........................................................A.. 418
CP000325       359 ................................T........................... 418
CP000325       359 ................................T........................... 418
CP000325       359 ................................T........................A.. 418
CP000325       359 ................................T........................... 418
CP000325       359 ............................................................ 418
CP000325       359 ............................................................ 418
CP000325       359 ...................................................C.....A.. 418
CP000325       359 .........................................................A.. 418
CP000325       359 ............................................................ 418
CP000325       359 .........................................................A.. 418
CP000325       359 ................................T........................... 418
CP000325       359 .........................................................A.. 418
CP000325       359 .........................................................A.. 418
CP000325       359 ................................T........................... 418
CP000325       359 .........................................................A.. 418
CP000325       359 .........................................................A.. 418
```

```
CP000325    359 ............................................................ 418
CP000325    359 ............................................................ 418
CP000325    359 .........................................................A.. 418
CP000325    359 ................................T........................... 418
CP000325    359 ................................T........................... 418
CP000325    359 ................................T........................... 418
CP000325    359 .........................................................A.. 418
CP000325    359 .........................................................A.. 418
CP000325    359 .........................................................A.. 418
CP000325    359 ................................T........................... 418
CP000325    359 ................................T........................A.. 418
CP000325    359 ................................T........................A.. 418
CP000325    359 ............................................................ 418
CP000325    359 .........................................................A.. 418
CP000325    359 ................................T........................... 418
CP000325    359 .........................................................A.. 418
CP000325    359 ................................T........................A.. 418
CP000325    359 ............................................................ 418
CP000325    358 .........................................................A.. 417
CP000325    359 ................................T........................... 418
CP000325    359 ....................................................A....A.. 418
CP000325    359 ................................T........................A.. 418
CP000325    358 ................................T........................A.. 417
CP000325    359 ................................T........................... 418
CP000325    359 .........................................................A.. 418
CP000325      0 ------------------------------------------------------------ 0
CP000325    359 ....................................................A....... 418
CP000325    359 .........................................................A.. 418
CP000325    359 .........................................................A.. 418
CP000325    359 ................................T........................A.. 418
CP000325    359 ................................T........................... 418
CP000325    359 .........................................................A.. 418
CP000325    359 .........................................................A.. 418
CP000325    359 .........................................................A.. 418
CP000325    359 ................................T.......T................A.. 418
CP000325    359 ............................................................ 418
CP000325    359 .........................................................A.. 418
CP000325    359 .........................................................A.. 418
CP000325    359 .........................................................A.. 418
CP000325    359 .........................................................A.. 418
CP000325    359 .........................................................A.. 418
CP000325    359 .........................................................A.. 418
CP000325    359 ................................T........................... 418
CP000325    359 ................................T........................... 418
CP000325    359 ............................................................ 418
CP000325    359 ............................................................ 418
CP000325    359 ................................T........................A.. 418
CP000325    359 ................................T........................A.. 418
CP000325    359 ....................................................A....A.. 418
CP000325    359 ................................T...................A....A.. 418
CP000325    359 ................................T........................... 418
CP000325    359 ................................T........................A.. 418
CP000325    359 .........................................................A.. 418
CP000325    359 .........................................................A.. 418
CP000325    361 .........................................................A.. 420
CP000325    359 ................................T........................A.. 418
CP000325    359 ................................T........................A.. 418
CP000325    359 ................................T........................A.. 418
CP000325    359 ................................T........................A.. 418
CP000325    359 ................................T........................A.. 418
CP000325    359 ...................................................C........ 418
CP000325    359 .........................................................A.. 418
CP000325    359 ................................T........................A.. 418
CP000325    359 ................................T........................A.. 418
CP000325    359 .........................................................A.. 418
CP000325    359 .........................................................A.. 418
CP000325    359 ................................T........................A.. 418
CP000325    359 .........................................................A.. 418
CP000325    359 .........................................................A.. 418
CP000325    359 .........................................................A.. 418
CP000325    359 .........................................................A.. 418
CP000325    359 .........................................................A.. 418
CP000325    359 .........................................................A.. 418
CP000325    359 .........................................................A.. 418
CP000325    359 .........................................................A.. 418
CP000325    359 .........................................................A.. 418
CP000325    359 .........................................................A.. 418
```

```
CP000325             359 .........................................................A.. 418
CP000325             359 ............................................................ 418
CP000325             359 ................................T........................A.. 418
CP000325             359 .........................................................A.. 418
CP000325             359 ............A............................................A.. 418
CP000325             359 .........................................................A.. 418
CP000325             359 .........................................................A.. 418
CP000325             359 .........................................................A.. 418
CP000325             359 ............................................................ 418
CP000325              29 ............................................................ 88
CP000325             359 .........................................................A.. 418
CP000325             359 .........................................................A.. 418
CP000325             359 .........................................................A.. 418
CP000325             359 .........................................................A.. 418
CP000325             359 .........................................................A.. 418
CP000325             359 .........................................................A.. 418
CP000325             359 .........................................................A.. 418
CP000325             359 ............................................................ 418
CP000325             359 ................................T........................... 418
CP000325             359 ................................T........................... 418
CP000325             359 ................................T........................... 418
CP000325             359 ................................T........................... 418
CP000325             359 ................................T........................... 418
CP000325             359 ................................T........................... 418
CP000325             359 ................................T........................... 418
CP000325             359 ................................T........T.................. 418
CP000325             359 ................................T........................... 418
CP000325             359 ....................................................A....... 418
CP000325             359 ................................T........................A.. 418
CP000325             359 ............A...................T........................A.. 418
CP000325             359 ................................T.......T................A.. 418
CP000325             359 ................................T...................A....A.. 418
CP000325             359 ................................T........................... 418
CP000325             197 ------------------------------------------------------------ 197

(query)MUL_0099.prj  419 GCTTG-CGGTGGCTGGTCACTGTGGATGCGATGCATACCCAGGTCGTCACCGCGAAGTTG 477
CP000325             170 ------------------------------------------------------------ 170
CP000325             419 A....-..............C.C..................................... 477
CP000325             419 A....-..............C...........A........................... 477
CP000325             419 .....-..............C....................................... 477
CP000325             419 .....C..............C....................................... 478
CP000325             421 A....-..............C....................................... 479
CP000325             419 A....-..............C....................................... 477
CP000325             419 .....-..............C...............................T....... 477
CP000325             419 .....-..............C....................................... 477
CP000325             419 .....-...........T..C....................................... 477
CP000325             419 A....-...........T..C............................T.......... 477
CP000325             419 .....-.............TC....................................... 477
CP000325             419 .....-...........T...............................T.......... 477
CP000325             419 .....-..............C....................................... 477
CP000325             419 A....-...................................................... 477
CP000325             419 .....-...................................................... 477
CP000325             419 .....-..............C....................................... 477
CP000325             419 .....-..............C....................................... 477
CP000325             419 .....-..............C....................................... 477
CP000325             419 .....-..............C....................................... 477
CP000325             418 A....-..............C....................................... 476
CP000325             419 A....-..............C....................................... 477
CP000325             193 ------------------------------------------------------------ 193
CP000325             258 A....-..............C....................................... 316
CP000325              61 ------------------------------------------------------------ 61
CP000325             252 A....-..............C....................................... 310
CP000325             419 A....-..............C....................................... 477
CP000325             419 A....-..............C....................................... 477
CP000325             419 A....-..............C....................................... 477
CP000325             419 A....-..............C....................................... 477
CP000325             419 A....-..............C....................................... 477
CP000325             419 A....-..............C....................................... 477
CP000325             419 A....-...........T.......................................... 477
CP000325             418 .....-..............C....................................... 476
CP000325             419 A....-...........T..C....................................... 477
CP000325             419 A....-...........T.......................................... 477
CP000325             419 .....-..............C....................................... 477
CP000325             419 A....-..............C....................................... 477
CP000325             419 .....-..............C....................................... 477
CP000325             419 A....-..............C...........A........................... 477
CP000325             419 A....-...........T.......................................... 477
```

```
CP000325                  419 .....-..............C...................................... 477
CP000325                  419 A....-..............C...................................... 477
CP000325                  419 A....-..............C...................................... 477
CP000325                  419 .....-..............C...................................... 477
CP000325                  419 A....-..............C...................................... 477
CP000325                  419 A....-..............C...................................... 477
CP000325                  419 .....-..............C...................................... 477
CP000325                  419 .....-..............C...................................... 477
CP000325                  419 .....-..............C...................................... 477
CP000325                  419 .....-..............C...................................... 477
CP000325                  419 .....-..............C...................................... 477
CP000325                  419 A....-..................................................... 477
CP000325                  419 .....-..............C...................................... 477
CP000325                  419 .....-..............C...................................... 477
CP000325                  419 .....-..............C...................................... 477
CP000325                  419 A....-..............C...................................... 477
CP000325                  419 A....-..............C...................................... 477
CP000325                  419 A....-..............C...................................... 477
CP000325                  419 A....-....A.........C...................................... 477
CP000325                  419 A....-..............C...........A.........................A 477
CP000325                  419 A....-..............C...................................... 477
CP000325                  419 A....-...........T......................................... 477
CP000325                  419 .....-..............C...................................... 477
CP000325                  418 A....-..............C...................................... 476
CP000325                  419 A....-..............C...................................... 477
CP000325                  419 .....-..................................................... 477
CP000325                  419 A....-..............C...................................... 477
CP000325                  419 A....-..............C...................................... 477
CP000325                  419 A....-..............C...................................... 477
CP000325                  419 .....-..............C...................................... 477
CP000325                  419 A....-..............C...........A.......................... 477
CP000325                  419 .....-...........T......................................... 477
CP000325                  419 A....-..............C...................................... 477
CP000325                  418 A....-..............C...................................... 476
CP000325                  419 .....-...........T..C...................................... 477
CP000325                    1 -----------------------------------------.................. 18
CP000325                  419 .....-..............C...................................... 477
CP000325                  419 .....-...........T..C...................................... 477
CP000325                  419 A....-..............C...................................... 477
CP000325                    0 ----------------------------------------------------------- 0
CP000325                  419 A....-..............C...................................... 477
CP000325                  419 A....-..............C...........A.......................... 477
CP000325                    0 ----------------------------------------------------------- 0
CP000325                  419 .....-..................................................... 477
CP000325                  419 .....-..................................................... 477
CP000325                  419 .....-..................................................... 477
CP000325                  419 A....-..............C...................................... 477
CP000325                  419 .....-..............C...................................... 477
CP000325                  419 .....-..............C...................................... 477
CP000325                  419 .....-..............C...................................... 477
CP000325                  419 A....-..............C...................................... 477
CP000325                  419 A....-..............C...................................... 477
CP000325                  419 .....-..............C...................................... 477
CP000325                  419 A....-..............C...................................... 477
CP000325                  419 .....-..............C...................................... 477
CP000325                  419 A....-..............C...................................... 477
CP000325                  419 A....-..............C...................................... 477
CP000325                  419 .....-..............C...................................... 477
CP000325                  419 A....-..............C...................................... 477
CP000325                  419 A....-..............C...................................... 477
CP000325                  419 .....-..............C...................................... 477
CP000325                  419 .....-..............C...................................... 477
CP000325                  419 .....-...........T..C...................................... 477
CP000325                  419 .....-..............C...................................... 477
CP000325                  419 .....-...........T......................................... 477
CP000325                  419 A....-..............C...................................... 477
CP000325                  419 A....-...........T..C...................................... 477
CP000325                  419 A....-..............C...................................... 477
CP000325                  419 A....-..............C...................................... 477
CP000325                  419 .....-...........T......................................... 477
CP000325                  419 A....-..........................A.......................... 477
CP000325                  419 A....-..............C...................................... 477
CP000325                  419 .....-...........T......................................... 477
CP000325                  419 A....-..............C...................................... 477
CP000325                  419 .....-..................................................... 477
CP000325                  419 A....-..............C...................................... 477
CP000325                  419 A....-..............C...........A.......................... 477
```

```
CP000325            419 .....-........A.....C....................................... 477
CP000325            418 A....-..............C....................................... 476
CP000325            419 .....-..............C....................................... 477
CP000325            419 A....-..............C...........A........................... 477
CP000325            419 A....-..............C....................................... 477
CP000325            418 A....-..............C....................................... 476
CP000325            419 .....-..............C....................................... 477
CP000325            419 A....-..............C....................................... 477
CP000325              0 ------------------------------------------------------------ 0
CP000325            419 .....-........A.....C....................................... 477
CP000325            419 A....-..............C....................................... 477
CP000325            419 A....-..............C....................................... 477
CP000325            419 A....-...........T.......................................... 477
CP000325            419 .....-..............C....................................... 477
CP000325            419 A....-..............C....................................... 477
CP000325            419 .....-..............C....................................... 477
CP000325            419 A....-..............C....................................... 477
CP000325            419 A....-T.............C....................................... 477
CP000325            419 .....-..............C....................................... 477
CP000325            419 A....-...........T.......................................... 477
CP000325            419 A....-..............C....................................... 477
CP000325            419 A....-..............C...........A........................... 477
CP000325            419 A....-..............C....................................... 477
CP000325            419 A....-..............C....................................... 477
CP000325            419 A....-..............C....................................... 477
CP000325            419 .....-..............C....................................... 477
CP000325            419 .....-..............C....................................... 477
CP000325            419 .....-..............C....................................... 477
CP000325            419 .....-..............C....................................... 477
CP000325            419 A....-..........................A........................... 477
CP000325            419 A....-..............C...........A........................... 477
CP000325            419 A....-...A..........C....................................... 477
CP000325            419 A....-....A.........C....................................... 477
CP000325            419 .....-..............C....................................... 477
CP000325            419 A....-..............C............................T.......... 477
CP000325            419 A....-..............C...........A........................... 477
CP000325            419 A....-...................................................... 477
CP000325            421 .....-..............C....................................... 479
CP000325            419 A....-..............C...........A........................... 477
CP000325            419 A....-..............C....................................... 477
CP000325            419 A....-..............C....................................... 477
CP000325            419 A....-..............C....................................... 477
CP000325            419 A....-..............C....................................... 477
CP000325            419 .....-..............C....................................... 477
CP000325            419 A....-..............C....................................... 477
CP000325            419 A....-..............C....................................... 477
CP000325            419 A....-..............C....................................... 477
CP000325            419 A....-..............C....................................... 477
CP000325            419 A....-..............C....................................... 477
CP000325            419 A....-..............C....................................... 477
CP000325            419 A....-...........T.......................................... 477
CP000325            419 A....-..............C....................................... 477
CP000325            419 A....-..............C....................................... 477
CP000325            419 A....-........A.....C....................................... 477
CP000325            419 A....-..............C....................................... 477
CP000325            419 A....-..............C....................................... 477
CP000325            419 A....-..............C....................................... 477
CP000325            419 A....-..............C....................................... 477
CP000325            419 A....-..............C....................................... 477
CP000325            419 A....-..............C...........A........................... 477
CP000325            419 A....-..............C...........A........................... 477
CP000325            419 .....-..............C...........A........................... 477
CP000325            419 A....-..............C....................................... 477
CP000325            419 A....-..............C....................................... 477
CP000325            419 A....-..............C....................................... 477
CP000325            419 A....-..............C....................................... 477
CP000325            419 A....-..............C....................................... 477
CP000325            419 A....-...........T..C......................................A 477
CP000325            419 .....-..............C...........A........................... 477
CP000325             89 .....-..............C....................................... 147
CP000325            419 A....-..............C....................................... 477
CP000325            419 A....-..............C....................................... 477
CP000325            419 A....-..............C....................................... 477
CP000325            419 A....-..............C....................................... 477
CP000325            419 A....-..............C....................................... 477
CP000325            419 A....-..............C....................................... 477
CP000325            419 A....-..............C....................................... 477
```

```
CP000325              419 .....-..............C....................................... 477
CP000325              419 .....-..............C....................................... 477
CP000325              419 .....-..............C....................................... 477
CP000325              419 .....-..............C....................................... 477
CP000325              419 .....-..............C....................................... 477
CP000325              419 .....-..............C....................................... 477
CP000325              419 .....-..............C....................................... 477
CP000325              419 .....-..............C....................................... 477
CP000325              419 .....-T.............C....................................... 477
CP000325              419 .....-...........T.......................................... 477
CP000325              419 .....-..............C....................................... 477
CP000325              419 A....-..............C....................................... 477
CP000325              419 A....-..............C....................................... 477
CP000325              419 A....-..............C....................................... 477
CP000325              419 A....-..............C...........A........................... 477
CP000325              419 .....-..............C....................................... 477
CP000325              197 ------------------------------------------------------------ 197

(query)MUL_0099.prj   478 ATCTGCGCCACCTTGAAGTCGCACTAC--------------------CTGATGATCGTCA 517
CP000325              170 ------------------------------------------------------------ 170
CP000325              478 ...........................--------------------............. 517
CP000325              478 ...........................--------------------............. 517
CP000325              478 ...........................--------------------T............ 517
CP000325              479 ...........................--------------------............. 518
CP000325              480 ...........................--------------------............. 519
CP000325              478 ...........................--------------------.......C..... 517
CP000325              478 ...........................--------------------............. 517
CP000325              478 ...........................--------------------............. 517
CP000325              478 ...........................--------------------............. 517
CP000325              478 ...........................--------------------T............ 517
CP000325              478 ...........................--------------------............. 517
CP000325              478 ...........................--------------------............. 517
CP000325              478 ...........................--------------------............. 517
CP000325              478 ...........................--------------------............. 517
CP000325              478 ...........................--------------------............. 517
CP000325              478 ...........................--------------------............. 517
CP000325              478 ...........................--------------------............. 517
CP000325              478 ...........................--------------------............. 517
CP000325              478 ...........................--------------------............. 517
CP000325              477 ...........................--------------------............. 516
CP000325              478 ...........................--------------------............. 517
CP000325              193 ------------------------------------------------------------ 193
CP000325              317 ...........................--------------------............. 356
CP000325               61 ------------------------------------------------------------ 61
CP000325              311 ...........................--------------------............. 350
CP000325              478 ...........................--------------------............. 517
CP000325              478 ...........................--------------------............. 517
CP000325              478 ...........................--------------------............. 517
CP000325              478 ...........................--------------------............. 517
CP000325              478 ...........................--------------------............. 517
CP000325              478 ...........................--------------------............. 517
CP000325              478 ...........................--------------------............. 517
CP000325              477 ...........................--------------------............. 516
CP000325              478 ...........................--------------------............. 517
CP000325              478 ...........................--------------------............. 517
CP000325              478 ...........................--------------------............. 517
CP000325              478 ...........................--------------------............. 517
CP000325              478 ...........................--------------------............. 517
CP000325              478 ..........A................--------------------............. 517
CP000325              478 ...........................--------------------............. 517
CP000325              478 ...........................--------------------............. 517
CP000325              478 ...........................--------------------............. 517
CP000325              478 ...........................--------------------............. 517
CP000325              478 ...........................--------------------............. 517
CP000325              478 ...........................--------------------............. 517
CP000325              478 ...........................--------------------............. 517
CP000325              478 ...........................--------------------............. 517
CP000325              478 ...........................--------------------............. 517
CP000325              478 ...........................--------------------............. 517
CP000325              478 ...........................--------------------............. 517
CP000325              478 ...........................--------------------............. 517
CP000325              478 ...........................--------------------............. 517
CP000325              478 ...........................--------------------............. 517
CP000325              478 ...........................--------------------............. 517
CP000325              478 ...........................--------------------............. 517
CP000325              478 ...........................--------------------............. 517
```

```
CP000325    478 ...........................--------------------............. 517
CP000325    478 ...........................--------------------............. 517
CP000325    478 ...........................--------------------............. 517
CP000325    478 ...........................--------------------............. 517
CP000325    478 ...........................--------------------............. 517
CP000325    478 ...........................--------------------............. 517
CP000325    477 ...........................--------------------............. 516
CP000325    478 ...........................--------------------............. 517
CP000325    478 ...........................--------------------............. 517
CP000325    478 ...........................--------------------............. 517
CP000325    478 ...........................--------------------............. 517
CP000325    478 ...........................--------------------............. 517
CP000325    478 ...........................--------------------............. 517
CP000325    478 ...........................--------------------............. 517
CP000325    478 ...........................--------------------............. 517
CP000325    478 ...........................--------------------............. 517
CP000325    477 ...........................--------------------............. 516
CP000325    478 ...........................--------------------............. 517
CP000325     19 ...........................--------------------............. 58
CP000325    478 ...........................--------------------............. 517
CP000325    478 ...........................--------------------T............ 517
CP000325    478 ...........................--------------------............. 517
CP000325      0 ------------------------------------------------------------ 0
CP000325    478 ...........................--------------------............. 517
CP000325    478 ...........................--------------------............. 517
CP000325      0 ------------------------------------------------------------ 0
CP000325    478 ...........................--------------------............. 517
CP000325    478 ...........................--------------------............. 517
CP000325    478 ...........................--------------------............. 517
CP000325    478 ...........................--------------------............. 517
CP000325    478 ...........................--------------------............. 517
CP000325    478 ...........................--------------------............. 517
CP000325    478 ...........................--------------------............. 517
CP000325    478 ...........................--------------------............. 517
CP000325    478 ...........................--------------------............. 517
CP000325    478 ...........................--------------------............. 517
CP000325    478 ...........................--------------------............. 517
CP000325    478 ...........................--------------------............. 517
CP000325    478 ...........................--------------------............. 517
CP000325    478 ...........................--------------------............. 517
CP000325    478 ...........................--------------------............. 517
CP000325    478 ...........................--------------------............. 517
CP000325    478 ..........T................--------------------............. 517
CP000325    478 ...........................--------------------............. 517
CP000325    478 ...........................--------------------............. 517
CP000325    478 ...........................--------------------............. 517
CP000325    478 ...........................--------------------............. 517
CP000325    478 ...........................--------------------............. 517
CP000325    478 ...........................--------------------............. 517
CP000325    478 ...........................--------------------............. 517
CP000325    478 ...........................--------------------............. 517
CP000325    478 ...........................--------------------............. 517
CP000325    478 ...........................--------------------............. 517
CP000325    478 ...........................--------------------............. 517
CP000325    478 ...........................--------------------............. 517
CP000325    478 ...........................--------------------............. 517
CP000325    478 ...........................--------------------............. 517
CP000325    478 ...........................--------------------............. 517
CP000325    478 ...........................--------------------............. 517
CP000325    478 ...........................--------------------............. 517
CP000325    478 ...........................--------------------............. 517
CP000325    477 ...........................--------------------............. 516
CP000325    478 ...........................--------------------............. 517
CP000325    478 ...........................--------------------............. 517
CP000325    478 ...........................--------------------............. 517
CP000325    477 ...........................--------------------............. 516
CP000325    478 ...........................--------------------............. 517
CP000325    478 ...........................--------------------............. 517
CP000325      0 ------------------------------------------------------------ 0
CP000325    478 ...........................--------------------............. 517
CP000325    478 ...........................--------------------............. 517
CP000325    478 ...........................--------------------............. 517
CP000325    478 ..........T................--------------------T............ 517
CP000325    478 ..........T................--------------------............. 517
CP000325    478 ...........................--------------------............. 517
CP000325    478 ...........................--------------------............. 517
CP000325    478 ...........................--------------------............. 517
```

```
CP000325          478 ...........................--------------------............. 517
CP000325          478 ..........T................--------------------............. 517
CP000325          478 ...........................--------------------............. 517
CP000325          478 ...........................--------------------............. 517
CP000325          478 ...........................--------------------............. 517
CP000325          478 ...........................--------------------............. 517
CP000325          478 ...........................--------------------............. 517
CP000325          478 ...........................--------------------............. 517
CP000325          478 ...........................--------------------T............ 517
CP000325          478 ...........................--------------------T............ 517
CP000325          478 ...........................--------------------T............ 517
CP000325          478 ...........................--------------------T............ 517
CP000325          478 ...........................--------------------............. 517
CP000325          478 ...........................--------------------............. 517
CP000325          478 ...........................--------------------............. 517
CP000325          478 ...........................--------------------............. 517
CP000325          478 ...........................--------------------............. 517
CP000325          478 ...........................--------------------............. 517
CP000325          478 ..........T................--------------------............. 517
CP000325          478 ...........................--------------------............. 517
CP000325          480 ...........................--------------------............. 519
CP000325          478 ...........................--------------------............. 517
CP000325          478 ...........................--------------------............. 517
CP000325          478 ...........................--------------------............. 517
CP000325          478 ...........................--------------------............. 517
CP000325          478 ...........................--------------------............. 517
CP000325          478 ...........................--------------------............. 517
CP000325          478 ...........................--------------------............. 517
CP000325          478 ...........................--------------------............. 517
CP000325          478 ...........................CTGATGATCGTCAAGTCCAA............. 537
CP000325          478 ...........................--------------------............. 517
CP000325          478 ...........................--------------------............. 517
CP000325          478 ..........T................--------------------............. 517
CP000325          478 ...........................--------------------............. 517
CP000325          478 ...........................--------------------............. 517
CP000325          478 ...........................--------------------............. 517
CP000325          478 ...........................--------------------............. 517
CP000325          478 ...........................--------------------............. 517
CP000325          478 ...........................--------------------............. 517
CP000325          478 ...........................--------------------............. 517
CP000325          478 ...........................--------------------............. 517
CP000325          478 ...........................--------------------............. 517
CP000325          478 ...........................--------------------............. 517
CP000325          478 ...........................--------------------............. 517
CP000325          478 ...........................--------------------............. 517
CP000325          478 ...........................--------------------............. 517
CP000325          478 ...........................--------------------............. 517
CP000325          478 ...........................--------------------............. 517
CP000325          478 ...........................--------------------............. 517
CP000325          478 ...........................--------------------............. 517
CP000325          478 ...........................--------------------............. 517
CP000325          478 ...........................--------------------............. 517
CP000325          148 ...........................--------------------............. 187
CP000325          478 ..........T................--------------------............. 517
CP000325          478 ...........................--------------------............. 517
CP000325          478 ...........................--------------------............. 517
CP000325          478 ...........................--------------------............. 517
CP000325          478 ...........................--------------------............. 517
CP000325          478 ...........................--------------------............. 517
CP000325          478 ...........................--------------------............. 517
CP000325          478 ...........................--------------------............. 517
CP000325          478 ...........................--------------------............. 517
CP000325          478 ...........................--------------------............. 517
CP000325          478 ...........................--------------------............. 517
CP000325          478 ...........................--------------------............. 517
CP000325          478 ...........................--------------------............. 517
CP000325          478 ...........................--------------------............. 517
CP000325          478 ...........................--------------------............. 517
CP000325          478 ...........................--------------------T............ 517
CP000325          478 ...........................--------------------............. 517
CP000325          478 ...........................--------------------............. 517
CP000325          478 ...........................--------------------............. 517
CP000325          478 ...........................--------------------............. 517
CP000325          478 ...........................--------------------............. 517
CP000325          478 ...........................--------------------............. 517
CP000325          478 ...........................--------------------............. 517
CP000325          197 ------------------------------------------------------------ 197
```

```
(query)MUL_0099.prj  518 AGTCCAACCAAGCCAAAATACTTGCCCGTATCACCGCGCTGCCCTGGGCCGAGGTGCCCG 577
CP000325             170 ------------------------------------------------------------ 170
CP000325             518 ..............G............................................. 577
CP000325             518 ..............G............................................. 577
CP000325             518 .........................G.................................. 577
CP000325             519 ............................................................ 578
CP000325             520 ............................................................ 579
CP000325             518 ............................................................ 577
CP000325             518 ..........G................................................. 577
CP000325             518 ............................................................ 577
CP000325             518 ............................................................ 577
CP000325             518 ..........G................................................. 577
CP000325             518 ............................................................ 577
CP000325             518 ............................................................ 577
CP000325             518 ............................................................ 577
CP000325             518 ............................................................ 577
CP000325             518 ............................................................ 577
CP000325             518 ............................................................ 577
CP000325             518 ............................................................ 577
CP000325             518 ............................................................ 577
CP000325             518 ............................................................ 577
CP000325             517 ............................................................ 576
CP000325             518 ............................................................ 577
CP000325             193 ------------------------------------------------------------ 193
CP000325             357 .........................G.................................. 416
CP000325              61 ------------------------------------------------------------ 61
CP000325             351 ............................................................ 410
CP000325             518 ............................................................ 577
CP000325             518 ............................................................ 577
CP000325             518 ............................................................ 577
CP000325             518 ............................................................ 577
CP000325             518 ............................................................ 577
CP000325             518 ............................................................ 577
CP000325             518 ............................................................ 577
CP000325             517 ............................................................ 576
CP000325             518 ............................................................ 577
CP000325             518 .........................G.................................. 577
CP000325             518 .........................G.................................. 577
CP000325             518 ..............G..........G.................................. 577
CP000325             518 ............................................................ 577
CP000325             518 ............................................................ 577
CP000325             518 ............................................................ 577
CP000325             518 .........................G.................................. 577
CP000325             518 ............................................................ 577
CP000325             518 ............................................................ 577
CP000325             518 ............................................................ 577
CP000325             518 ............................................................ 577
CP000325             518 ............................................................ 577
CP000325             518 ............................................................ 577
CP000325             518 ............................................................ 577
CP000325             518 ............................................................ 577
CP000325             518 .........................G.................................. 577
CP000325             518 ..........G................................................. 577
CP000325             518 .......................................................A.... 577
CP000325             518 ............................................................ 577
CP000325             518 .........................G.................................. 577
CP000325             518 ............................................................ 577
CP000325             518 ............................................................ 577
CP000325             518 ............................................................ 577
CP000325             518 ............................................................ 577
CP000325             518 ............................................................ 577
CP000325             518 ............................................................ 577
CP000325             518 ............................................................ 577
CP000325             518 ............................................................ 577
CP000325             518 ............................................................ 577
CP000325             517 ............................................................ 576
CP000325             518 ............................................................ 577
CP000325             518 ............................................................ 577
CP000325             518 ............................................................ 577
CP000325             518 ............................................................ 577
CP000325             518 ............................................................ 577
CP000325             518 ............................................................ 577
CP000325             518 ............................................................ 577
CP000325             518 .........................G.................................. 577
CP000325             518 ............................................................ 577
CP000325             517 ............................................................ 576
CP000325             518 ......------------------------------------------------------ 523
```

```
CP000325   59 ............................................................ 118
CP000325  518 .........................G.................................. 577
CP000325  518 ..........G................................................. 577
CP000325  518 ............................................................ 577
CP000325    0 ------------------------------------------------------------ 0
CP000325  518 ............................................................ 577
CP000325  518 ............................................................ 577
CP000325    1 ------------------------------------------------------...... 6
CP000325  518 ............................................................ 577
CP000325  518 ............................................................ 577
CP000325  518 .........................G.................................. 577
CP000325  518 ............................................................ 577
CP000325  518 ............................................................ 577
CP000325  518 ............................................................ 577
CP000325  518 ............................................................ 577
CP000325  518 ............................................................ 577
CP000325  518 ............................................................ 577
CP000325  518 ...............................A............................ 577
CP000325  518 .....................................C...................... 577
CP000325  518 ............................................................ 577
CP000325  518 .........................G.................................. 577
CP000325  518 ............................................................ 577
CP000325  518 ............................................................ 577
CP000325  518 .........................G.................................. 577
CP000325  518 .........................G.................................. 577
CP000325  518 ............................................................ 577
CP000325  518 ............................................................ 577
CP000325  518 .........................G.................................. 577
CP000325  518 ............................................................ 577
CP000325  518 ............................................................ 577
CP000325  518 ............................................................ 577
CP000325  518 ............................................................ 577
CP000325  518 ............................................................ 577
CP000325  518 ............................................................ 577
CP000325  518 .........................G.................................. 577
CP000325  518 ............................................................ 577
CP000325  518 ............................................................ 577
CP000325  518 .........................G.................................. 577
CP000325  518 .........................G.................................. 577
CP000325  518 ............................................................ 577
CP000325  518 ............................................................ 577
CP000325  518 ............................................................ 577
CP000325  518 ............................C............................... 577
CP000325  517 ..............G............................................. 576
CP000325  518 ............................................................ 577
CP000325  518 ............................................................ 577
CP000325  518 ............................................................ 577
CP000325  517 ............................................................ 576
CP000325  518 ............................................................ 577
CP000325  518 ............................................................ 577
CP000325    0 ------------------------------------------------------------ 0
CP000325  518 ............................................................ 577
CP000325  518 ............................................................ 577
CP000325  518 ............................................................ 577
CP000325  518 .........................G.................................. 577
CP000325  518 ..............G..........G.................................. 577
CP000325  518 ............................................................ 577
CP000325  518 ............................................................ 577
CP000325  518 ............................................................ 577
CP000325  518 ............................................................ 577
CP000325  518 .........................G.................................. 577
CP000325  518 ..........G................................................. 577
CP000325  518 ............................................................ 577
CP000325  518 ............................................................ 577
CP000325  518 ............................................................ 577
CP000325  518 ............................................................ 577
CP000325  518 ............................................................ 577
CP000325  518 ..............G..........G.................................. 577
CP000325  518 ............................................................ 577
CP000325  518 ............................................................ 577
CP000325  518 ............................................................ 577
CP000325  518 ............................................................ 577
CP000325  518 ............................................................ 577
CP000325  518 ............................................................ 577
CP000325  518 ............................................................ 577
CP000325  518 ............................................................ 577
CP000325  518 ............................................................ 577
```

```
CP000325             518 ............................................................ 577
CP000325             518 ............................................................ 577
CP000325             520 ............................................................ 579
CP000325             518 ............................................................ 577
CP000325             518 ............................................................ 577
CP000325             518 ............................................................ 577
CP000325             518 ............................................................ 577
CP000325             518 ............................................................ 577
CP000325             518 ............................................................ 577
CP000325             518 ............................................................ 577
CP000325             518 ............................................................ 577
CP000325             538 ............................................................ 597
CP000325             518 ............................................................ 577
CP000325             518 ............................................................ 577
CP000325             518 ............................................................ 577
CP000325             518 ............................................................ 577
CP000325             518 ..C......................................................... 577
CP000325             518 ..C......................G.................................. 577
CP000325             518 ............................................................ 577
CP000325             518 ............................................................ 577
CP000325             518 ............................................................ 577
CP000325             518 .........G.................................................. 577
CP000325             518 ............................................................ 577
CP000325             518 ............................................................ 577
CP000325             518 ............................................................ 577
CP000325             518 ............................................................ 577
CP000325             518 ............................................................ 577
CP000325             518 .........................G.................................. 577
CP000325             518 ............................................................ 577
CP000325             518 ............................................................ 577
CP000325             518 .........................G.................................. 577
CP000325             518 ............................................................ 577
CP000325             518 ............................................................ 577
CP000325             518 ............................................................ 577
CP000325             188 ............................................................ 247
CP000325             518 ............................................................ 577
CP000325             518 ............................................................ 577
CP000325             518 ............................................................ 577
CP000325             518 ............................................................ 577
CP000325             518 ............................................................ 577
CP000325             518 ............................................................ 577
CP000325             518 ............................................................ 577
CP000325             518 ..............G............................................. 577
CP000325             518 ............................................................ 577
CP000325             518 ............................................................ 577
CP000325             518 ............................................................ 577
CP000325             518 ............................................................ 577
CP000325             518 ............................................................ 577
CP000325             518 ............................................................ 577
CP000325             518 ............................................................ 577
CP000325             518 ............................................................ 577
CP000325             518 ............................................................ 577
CP000325             518 ..............G..........G.................................. 577
CP000325             518 .........................G.................................. 577
CP000325             518 .........................G.................................. 577
CP000325             518 ............................................................ 577
CP000325             518 ............................................................ 577
CP000325             518 ............................................................ 577
CP000325             197 ------------------------------------------------------------ 197

(query)MUL_0099.prj  578 CAGCCGCTACCGACGACTCCCGCGGCCACGGCCGTGTCGAGACCCGCACCCTGCAAATCA 637
CP000325             170 ------------------------------------------------------------ 170
CP000325             578 ......................................A..................... 637
CP000325             578 ......................................A..................... 637
CP000325             578 ............................................................ 637
CP000325             579 ......................................A..................... 638
CP000325             580 ......................................A..................... 639
CP000325             578 ......................................A..................... 637
CP000325             578 ......................................A..................... 637
CP000325             578 ............................................................ 637
CP000325             578 ............................................................ 637
CP000325             578 ............................................................ 637
CP000325             578 ......................................A..................... 637
CP000325             578 ............................................................ 637
CP000325             578 ............................................................ 637
CP000325             578 ............................................................ 637
CP000325             578 ......................................A..................... 637
```

```
CP000325        578 ......................................A..................... 637
CP000325        578 ......................................A..................... 637
CP000325        578 ............................................................ 637
CP000325        578 ......................................A..................... 637
CP000325        577 ............................................................ 636
CP000325        578 ............................................................ 637
CP000325        193 ------------------------------------------------------------ 193
CP000325        417 ......................................A..................... 476
CP000325         61 ------------------------------------------------------------ 61
CP000325        411 ......................................A..................... 470
CP000325        578 ............................................................ 637
CP000325        578 ......................................A..................... 637
CP000325        578 ......................................A..................... 637
CP000325        578 ......................................A..................... 637
CP000325        578 ......................................A..................... 637
CP000325        578 ......................................A..................... 637
CP000325        578 ............................................................ 637
CP000325        577 ............................................................ 636
CP000325        578 ............................................................ 637
CP000325        578 ............................................................ 637
CP000325        578 ............................................................ 637
CP000325        578 ............................................................ 637
CP000325        578 ......................................A..................... 637
CP000325        578 ............................................................ 637
CP000325        578 ............................................................ 637
CP000325        578 ............................................................ 637
CP000325        578 ............................................................ 637
CP000325        578 ............................................................ 637
CP000325        578 ......................................A..................... 637
CP000325        578 ............................................................ 637
CP000325        578 ............................................................ 637
CP000325        578 ......................................A..................... 637
CP000325        578 ............................................................ 637
CP000325        578 ............................................................ 637
CP000325        578 ............................................................ 637
CP000325        578 ............................................................ 637
CP000325        578 ............................................................ 637
CP000325        578 ............................................................ 637
CP000325        578 ......................................A..................... 637
CP000325        578 ......................................A..................... 637
CP000325        578 ............................................................ 637
CP000325        578 ......................................A..................... 637
CP000325        578 ............................................................ 637
CP000325        578 ............................................................ 637
CP000325        578 ......................................A..................... 637
CP000325        578 ......................................A..................... 637
CP000325        578 ............................................................ 637
CP000325        578 ............................................................ 637
CP000325        577 ......................................A..................... 636
CP000325        578 ............................................................ 637
CP000325        578 ............................................................ 637
CP000325        578 ............................................................ 637
CP000325        578 ............................................................ 637
CP000325        578 ............................................................ 637
CP000325        578 ......................................A..................... 637
CP000325        578 ............................................................ 637
CP000325        578 ............................................................ 637
CP000325        578 ............................................................ 637
CP000325        577 ............................................................ 636
CP000325        523 ------------------------------------------------------------ 523
CP000325        119 ............................................................ 178
CP000325        578 ............................................................ 637
CP000325        578 ............................................................ 637
CP000325        578 ............................................................ 637
CP000325          0 ------------------------------------------------------------ 0
CP000325        578 ............................................................ 637
CP000325        578 .........................................------------------- 618
CP000325          7 ............................................................ 66
CP000325        578 ............................................................ 637
CP000325        578 ............................................................ 637
CP000325        578 ............................................................ 637
CP000325        578 ............................................................ 637
CP000325        578 ............................................................ 637
CP000325        578 ............................................................ 637
CP000325        578 ............................................................ 637
CP000325        578 ............................................................ 637
CP000325        578 ............................................................ 637
```

```
CP000325          578 ............................................................ 637
CP000325          578 ............................................................ 637
CP000325          578 ....G....................................................... 637
CP000325          578 ......................................A..................... 637
CP000325          578 ......................................A..................... 637
CP000325          578 ............................................................ 637
CP000325          578 ............................................................ 637
CP000325          578 ............................................................ 637
CP000325          578 ......................................A..................... 637
CP000325          578 .................................................T.......... 637
CP000325          578 ............................................................ 637
CP000325          578 ............................................................ 637
CP000325          578 ............................................................ 637
CP000325          578 ......................................A..................... 637
CP000325          578 ......................................A..................... 637
CP000325          578 ......................................A..................... 637
CP000325          578 ......................................A..................... 637
CP000325          578 ............................................................ 637
CP000325          578 ......................................A..................... 637
CP000325          578 ......................................A..................... 637
CP000325          578 ......................................A..................... 637
CP000325          578 ......................................A..................... 637
CP000325          578 ...A...........................G......T..................... 637
CP000325          578 ......................................A..................... 637
CP000325          578 ............................................................ 637
CP000325          578 ......................................A..................... 637
CP000325          577 ............................................................ 636
CP000325          578 ....G....................................................... 637
CP000325          578 ......................................A..................... 637
CP000325          578 ......................................A..................... 637
CP000325          577 ......................................A..................... 636
CP000325          578 ............................................................ 637
CP000325          578 ......................................A..................... 637
CP000325            0 ------------------------------------------------------------ 0
CP000325          578 ......................................A..................... 637
CP000325          578 ......................................A..................... 637
CP000325          578 ......................................A..................... 637
CP000325          578 ......................................A..................... 637
CP000325          578 ....................T............................T.......... 637
CP000325          578 ......................................A..................... 637
CP000325          578 ......................................A..................... 637
CP000325          578 ..............................A.......A..................... 637
CP000325          578 ......................................A..................... 637
CP000325          578 ......................................A..................... 637
CP000325          578 ............................................................ 637
CP000325          578 ......................................A..................... 637
CP000325          578 ......................................A..................... 637
CP000325          578 ......................................A..................... 637
CP000325          578 ......................................A..................... 637
CP000325          578 ......................................A..................... 637
CP000325          578 ............................................................ 637
CP000325          578 ............................................................ 637
CP000325          578 ......................................A..................... 637
CP000325          578 ............................................................ 637
CP000325          578 ......................................A..................... 637
CP000325          578 ......................................A..................... 637
CP000325          578 ............................................................ 637
CP000325          578 ......................................A..................... 637
CP000325          578 ............................................................ 637
CP000325          578 ............................................................ 637
CP000325          578 ......................................A..................... 637
CP000325          578 ............................................................ 637
CP000325          580 ......................................A..................... 639
CP000325          578 ......................................A..................... 637
CP000325          578 .................................................T.......... 637
CP000325          578 ......................................A..................... 637
CP000325          578 ............................................................ 637
CP000325          578 ............................................................ 637
CP000325          578 ......................................A..................... 637
CP000325          578 ............................................................ 637
CP000325          578 ......................................A..................... 637
CP000325          598 ......................................A..................... 657
CP000325          578 ............................................................ 637
CP000325          578 ......................................A..................... 637
CP000325          578 ......................................A..................... 637
CP000325          578 ............................................................ 637
CP000325          578 ............................................................ 637
```

```
CP000325                578 ............................................................ 637
CP000325                578 ......................................A..................... 637
CP000325                578 ......................................A..................... 637
CP000325                578 ............................................................ 637
CP000325                578 ............................................................ 637
CP000325                578 .................................................T.......... 637
CP000325                578 ......................................A..................... 637
CP000325                578 ......................................A..................... 637
CP000325                578 ......................................A..................... 637
CP000325                578 ............................................................ 637
CP000325                578 ............................................................ 637
CP000325                578 ......................................A..................... 637
CP000325                578 ............................................................ 637
CP000325                578 ............................................................ 637
CP000325                578 ......................................A..................... 637
CP000325                578 ......................................A..................... 637
CP000325                578 ............................................................ 637
CP000325                248 ......................................A..................... 307
CP000325                578 ............................................................ 637
CP000325                578 ......................................A..................... 637
CP000325                578 ......................................A..................... 637
CP000325                578 ......................................A..................... 637
CP000325                578 ......................................A..................... 637
CP000325                578 ......................................A..................... 637
CP000325                578 ......................................A..................... 637
CP000325                578 ............................................................ 637
CP000325                578 ............................................................ 637
CP000325                578 ............................................................ 637
CP000325                578 ............................................................ 637
CP000325                578 ............................................................ 637
CP000325                578 ............................................................ 637
CP000325                578 ......................................A..................... 637
CP000325                578 ......................................A..................... 637
CP000325                578 ............................................................ 637
CP000325                578 ............................................................ 637
CP000325                578 ......................................A..................... 637
CP000325                578 ............................................................ 637
CP000325                578 ......................................A..................... 637
CP000325                578 ............................................................ 637
CP000325                578 ......................................A..................... 637
CP000325                578 ......................................A..................... 637
CP000325                197 ------------------------------------------------------------ 197

(query)MUL_0099.prj     638 TCACCGCTGCACGAGGAATC---------------------GGCTTCCCCTACGCAAAAC 676
CP000325                171 -----------------------------------------...---------------- 173
CP000325                638 .....A..............---------------------................... 676
CP000325                638 ....................---------------------................... 676
CP000325                638 ....................---------------------................... 676
CP000325                639 ....................---------------------................... 677
CP000325                640 ....................---------------------................... 678
CP000325                638 ....................---------------------.A................. 676
CP000325                638 ....................---------------------................... 676
CP000325                638 ....................---------------------................... 676
CP000325                638 ....................---------------------................... 676
CP000325                638 ....................---------------------................... 676
CP000325                638 ....................---------------------................... 676
CP000325                638 ....................---------------------.....T............. 676
CP000325                638 ....................---------------------................... 676
CP000325                638 .....A..............---------------------................... 676
CP000325                638 ....................---------------------................... 676
CP000325                638 ....................---------------------................... 676
CP000325                638 ....................---------------------................... 676
CP000325                638 ....................---------------------................... 676
CP000325                638 ....................---------------------................... 676
CP000325                637 ....................---------------------.....T............. 675
CP000325                638 ....................---------------------................... 676
CP000325                193 ------------------------------------------------------------ 193
CP000325                477 ....................---------------------................... 515
CP000325                 61 ------------------------------------------------------------ 61
CP000325                471 ..................C.---------------------................... 509
CP000325                638 ....................---------------------................... 676
CP000325                638 ....................---------------------................... 676
CP000325                638 ....................---------------------................... 676
CP000325                638 ....................---------------------................... 676
CP000325                638 ....................---------------------................... 676
CP000325                638 ....................---------------------................... 676
CP000325                638 ....................---------------------................... 676
```

```
CP000325            637 ....................---------------------................... 675
CP000325            638 ....................---------------------................... 676
CP000325            638 ....................---------------------....T.............. 676
CP000325            638 ....................---------------------................... 676
CP000325            638 ....................---------------------..T................ 676
CP000325            638 ....................---------------------................... 676
CP000325            638 ....................---------------------................... 676
CP000325            638 ....................---------------------................... 676
CP000325            638 ....................---------------------................... 676
CP000325            638 ....................---------------------................... 676
CP000325            638 ....................---------------------................... 676
CP000325            638 ..................C.---------------------................... 676
CP000325            638 ..................C.---------------------................... 676
CP000325            638 ..................C.---------------------................... 676
CP000325            638 ....................---------------------................... 676
CP000325            638 ....................---------------------................... 676
CP000325            638 ....................---------------------................... 676
CP000325            638 ....................---------------------................... 676
CP000325            638 ....................---------------------................... 676
CP000325            638 ....................---------------------................... 676
CP000325            638 ....................---------------------................... 676
CP000325            638 ....................---------------------................... 676
CP000325            638 ....................---------------------................... 676
CP000325            638 .....A..............---------------------................... 676
CP000325            638 ....................---------------------................... 676
CP000325            638 ....................---------------------................... 676
CP000325            638 ....................---------------------................... 676
CP000325            638 ....................---------------------................... 676
CP000325            638 ....................---------------------................... 676
CP000325            638 ....................---------------------................... 676
CP000325            638 ....................---------------------................... 676
CP000325            637 ....................---------------------................... 675
CP000325            638 ....................---------------------................... 676
CP000325            638 ....................---------------------................... 676
CP000325            638 ....................---------------------................... 676
CP000325            638 ....................---------------------................... 676
CP000325            638 ....................---------------------................... 676
CP000325            638 ....................---------------------................... 676
CP000325            638 ....................---------------------................... 676
CP000325            638 ....................---------------------................... 676
CP000325            638 ....................---------------------................... 676
CP000325            637 ....................---------------------................... 675
CP000325            523 ------------------------------------------------------------ 523
CP000325            179 ....................---------------------................... 217
CP000325            638 ....................---------------------................... 676
CP000325            638 ....................---------------------................... 676
CP000325            638 .....A..............---------------------................... 676
CP000325              0 ------------------------------------------------------------ 0
CP000325            638 ....................---------------------................... 676
CP000325            618 ------------------------------------------------------------ 618
CP000325             67 ....................---------------------................... 105
CP000325            638 ....................---------------------................... 676
CP000325            638 ....................---------------------................... 676
CP000325            638 ....................---------------------................... 676
CP000325            638 ....................---------------------................... 676
CP000325            638 ....................---------------------................... 676
CP000325            638 ....................---------------------................... 676
CP000325            638 ....................---------------------................... 676
CP000325            638 ....................---------------------................... 676
CP000325            638 ....................---------------------................... 676
CP000325            638 ....................---------------------................... 676
CP000325            638 ....................---------------------................... 676
CP000325            638 ....................---------------------................... 676
CP000325            638 ....................---------------------................... 676
CP000325            638 ....................---------------------................... 676
CP000325            638 ....................---------------------................... 676
CP000325            638 ....................---------------------................... 676
CP000325            638 ....................---------------------................... 676
CP000325            638 ....................---------------------................... 676
CP000325            638 ....................---------------------................... 676
CP000325            638 ....................---------------------................... 676
CP000325            638 ....................---------------------................... 676
CP000325            638 ....................---------------------................... 676
CP000325            638 ....................---------------------................... 676
CP000325            638 ....................---------------------................... 676
CP000325            638 ....................---------------------................... 676
CP000325            638 ....................---------------------................... 676
```

```
CP000325    638 ....................---------------------................... 676
CP000325    638 ....................---------------------................... 676
CP000325    638 ....................---------------------................... 676
CP000325    638 ....................---------------------................... 676
CP000325    638 ....................---------------------................... 676
CP000325    638 ....................---------------------................... 676
CP000325    638 ....................---------------------................... 676
CP000325    638 ....................---------------------................... 676
CP000325    638 ....................---------------------................... 676
CP000325    637 ....................---------------------................... 675
CP000325    638 ....................---------------------................... 676
CP000325    638 .....A..............---------------------................... 676
CP000325    638 ....................---------------------.....T............. 676
CP000325    637 ....................---------------------................... 675
CP000325    638 ....................GGCTTCCCCTACGCAAAACAA................... 697
CP000325    638 ....................---------------------................... 676
CP000325      0 ------------------------------------------------------------ 0
CP000325    638 ....................---------------------................... 676
CP000325    638 ....................---------------------................... 676
CP000325    638 .....A..............---------------------................... 676
CP000325    638 ....................---------------------................... 676
CP000325    638 ....................---------------------................... 676
CP000325    638 ....................---------------------................... 676
CP000325    638 ..................C.---------------------................... 676
CP000325    638 ....................---------------------................... 676
CP000325    638 ....................---------------------................... 676
CP000325    638 .....A..............---------------------................... 676
CP000325    638 ....................---------------------................... 676
CP000325    638 .....A..............---------------------................... 676
CP000325    638 ....................---------------------................... 676
CP000325    638 .....A..............---------------------................... 676
CP000325    638 ....................---------------------................... 676
CP000325    638 .....A..............---------------------................... 676
CP000325    638 ....................---------------------................... 676
CP000325    638 ....................---------------------................... 676
CP000325    638 ....................---------------------................... 676
CP000325    638 ....................---------------------................... 676
CP000325    638 ....................---------------------................... 676
CP000325    638 .....A..............---------------------................... 676
CP000325    638 ....................---------------------................... 676
CP000325    638 ....................---------------------................... 676
CP000325    638 ....................---------------------................... 676
CP000325    638 ....................---------------------................... 676
CP000325    638 ....................---------------------................... 676
CP000325    638 .....A..............---------------------................... 676
CP000325    640 ..................C.---------------------................... 678
CP000325    638 .....A..............---------------------................... 676
CP000325    638 ....................---------------------................... 676
CP000325    638 ....................---------------------................... 676
CP000325    638 ....................---------------------................... 676
CP000325    638 ....................---------------------................... 676
CP000325    638 ....................---------------------................... 676
CP000325    638 ....................---------------------................... 676
CP000325    638 ....................---------------------................... 676
CP000325    658 ....................---------------------................... 696
CP000325    638 ....................---------------------................... 676
CP000325    638 ....................---------------------................... 676
CP000325    638 .....A..............---------------------................... 676
CP000325    638 ....................---------------------................... 676
CP000325    638 ....................---------------------..T................ 676
CP000325    638 ....................---------------------................... 676
CP000325    638 .....A..............---------------------................... 676
CP000325    638 ....................---------------------................... 676
CP000325    638 ....................---------------------................... 676
CP000325    638 ....................---------------------................... 676
CP000325    638 ....................---------------------................... 676
CP000325    638 ....................---------------------................... 676
CP000325    638 .....A..............---------------------................... 676
CP000325    638 .....A..............---------------------................... 676
CP000325    638 .....A..............---------------------................... 676
CP000325    638 ....................---------------------................... 676
CP000325    638 ....................---------------------................... 676
CP000325    638 ....................---------------------................... 676
CP000325    638 ....................---------------------................... 676
CP000325    638 ....................---------------------................... 676
CP000325    638 ....................---------------------................... 676
CP000325    638 .....A..............---------------------................... 676
```

```
CP000325                308 .....A..............---------------------................... 346
CP000325                638 ....................---------------------................... 676
CP000325                638 ....................---------------------................... 676
CP000325                638 .....A..............---------------------................... 676
CP000325                638 ....................---------------------................... 676
CP000325                638 ....................---------------------................... 676
CP000325                638 ....................---------------------................... 676
CP000325                638 ....................---------------------................... 676
CP000325                638 ....................---------------------................... 676
CP000325                638 ....................---------------------................... 676
CP000325                638 ....................---------------------....C.............. 676
CP000325                638 ....................---------------------..T................ 676
CP000325                638 ....................---------------------................... 676
CP000325                638 ....................---------------------................... 676
CP000325                638 ....................---------------------................... 676
CP000325                638 .....A..............---------------------................... 676
CP000325                638 ....................---------------------................... 676
CP000325                638 ....................---------------------................... 676
CP000325                638 ....................---------------------..T................ 676
CP000325                638 ....................---------------------................... 676
CP000325                638 ....................---------------------................... 676
CP000325                638 ....................---------------------................... 676
CP000325                638 ....................---------------------................... 676
CP000325                638 ....................---------------------................... 676
CP000325                197 ------------------------------------------------------------ 197

(query)MUL_0099.prj     677 AAATCATCCGGATCACTCGTGAACGCTTGAT------------CACCGCCACCGACCAGC 724
CP000325                173 ------------------------------------------------------------ 173
CP000325                677 ...........................A...------------................. 724
CP000325                677 ...........................A...------------................. 724
CP000325                677 ...........................A...------------................. 724
CP000325                678 ...............................------------................. 725
CP000325                679 ............C..................------------................. 726
CP000325                677 ...............................------------................. 724
CP000325                677 ...............................------------................. 724
CP000325                677 ...............................------------................. 724
CP000325                677 ...............................------------................. 724
CP000325                677 ...............................------------................. 724
CP000325                677 ...............................------------................. 724
CP000325                677 ...............................------------................. 724
CP000325                677 ...............................------------................. 724
CP000325                677 ...............................------------................. 724
CP000325                677 ...............................------------................. 724
CP000325                677 ...............................------------................. 724
CP000325                677 ...............................------------................. 724
CP000325                677 ...............................------------................. 724
CP000325                677 ...............................------------................. 724
CP000325                676 ...............................------------................. 723
CP000325                677 ...............................------------................. 724
CP000325                193 ------------------------------------------------------------ 193
CP000325                516 ...............................------------................. 563
CP000325                 61 ------------------------------------------------------------ 61
CP000325                510 ...............................------------................. 557
CP000325                677 ...............................------------................. 724
CP000325                677 ...............................------------................. 724
CP000325                677 ...............................------------................. 724
CP000325                677 ...............................------------................. 724
CP000325                677 ...............................------------................. 724
CP000325                677 ...............................------------................. 724
CP000325                677 ...............................------------................. 724
CP000325                676 ...............................------------................. 723
CP000325                677 ...............................------------................. 724
CP000325                677 ...............................------------................. 724
CP000325                677 ...............................------------................. 724
CP000325                677 ...............................------------................. 724
CP000325                677 ...............................------------................. 724
CP000325                677 ...............................------------................. 724
CP000325                677 ...............................------------................. 724
CP000325                677 ...............................------------................. 724
CP000325                677 ............C..............A...------------................. 724
CP000325                677 ............C..............A...------------................. 724
CP000325                677 ...............................------------................. 724
CP000325                677 ...............................------------................. 724
CP000325                677 ...............................------------................. 724
CP000325                677 ...............................------------................. 724
CP000325                677 ...............................------------................. 724
CP000325                677 ...............................------------................. 724
```

```
CP000325        677 ...............................------------................. 724
CP000325        677 ...............................------------................. 724
CP000325        677 ...............................------------................. 724
CP000325        677 ...........................A...------------................. 724
CP000325        677 ...............................------------................. 724
CP000325        677 ...........................A...------------................. 724
CP000325        677 ...............................------------................. 724
CP000325        677 ...............................------------................. 724
CP000325        677 ...............................------------................. 724
CP000325        677 ...........................A...------------................. 724
CP000325        677 ...............................------------................. 724
CP000325        677 ...............................------------................. 724
CP000325        677 ...............................------------................. 724
CP000325        677 ...............................CACCGCCACCGC................. 736
CP000325        676 ...............................------------................. 723
CP000325        677 ...............................------------................. 724
CP000325        677 ...............................------------................. 724
CP000325        677 ...............................------------................. 724
CP000325        677 ............C..................------------................. 724
CP000325        677 ...............................------------................. 724
CP000325        677 ...............................------------................. 724
CP000325        677 ...............................------------................. 724
CP000325        677 ...............................------------................. 724
CP000325        677 ...............................------------................. 724
CP000325        676 ...............................------------................. 723
CP000325        523 ------------------------------------------------------------ 523
CP000325        218 ...............................------------................. 265
CP000325        677 ...............................------------................. 724
CP000325        677 ...............................------------................. 724
CP000325        677 ...........................A...------------................. 724
CP000325          0 ------------------------------------------------------------ 0
CP000325        677 ............C..................------------................. 724
CP000325        618 ------------------------------------------------------------ 618
CP000325        106 ...............................------------................. 153
CP000325        677 ...............................------------................. 724
CP000325        677 ...............................------------................. 724
CP000325        677 ...............................------------................. 724
CP000325        677 ...............................------------................. 724
CP000325        677 ...............................------------................. 724
CP000325        677 ...............................------------................. 724
CP000325        677 ...............................------------................. 724
CP000325        677 ...............................------------................. 724
CP000325        677 ...............................------------................. 724
CP000325        677 ...........................A...------------................. 724
CP000325        677 ...............................------------................. 724
CP000325        677 ...............................------------................. 724
CP000325        677 ...............................------------................. 724
CP000325        677 ...............................------------....A............ 724
CP000325        677 ...............................------------................. 724
CP000325        677 ...............................------------................. 724
CP000325        677 ...............................------------................. 724
CP000325        677 ...........................A...------------................. 724
CP000325        677 ...............................------------................. 724
CP000325        677 ...............................------------................. 724
CP000325        677 ...............................------------................. 724
CP000325        677 ...............................------------................. 724
CP000325        677 ...............................------------................. 724
CP000325        677 ...............................------------................. 724
CP000325        677 ...............................------------................. 724
CP000325        677 ...............................------------................. 724
CP000325        677 ...............................------------................. 724
CP000325        677 ...............................------------................. 724
CP000325        677 ...............................------------................. 724
CP000325        677 ...............................------------................. 724
CP000325        677 ...............................------------................. 724
CP000325        677 ...............................------------................. 724
CP000325        677 ............C..................------------................. 724
CP000325        677 ...............................------------................. 724
CP000325        677 ...............................------------................. 724
CP000325        676 ...............................------------................. 723
CP000325        677 ...............................------------................. 724
CP000325        677 ...........................A...------------................. 724
CP000325        677 ...............................------------................. 724
CP000325        676 ...............................------------................. 723
CP000325        698 ...............................------------................. 745
CP000325        677 ...............................------------................. 724
CP000325          0 ------------------------------------------------------------ 0
```

```
CP000325          677 ...........................A...------------................. 724
CP000325          677 ...........................A...------------................. 724
CP000325          677 ............C..............A...------------................. 724
CP000325          677 ...............................------------................. 724
CP000325          677 ...............................------------................. 724
CP000325          677 ............C..............A...------------................. 724
CP000325          677 ...........................A...------------................. 724
CP000325          677 ...........................A...------------................. 724
CP000325          677 ...............................------------................. 724
CP000325          677 ...............................------------................. 724
CP000325          677 ...............................------------................. 724
CP000325          677 ...........................A...------------................. 724
CP000325          677 ...............................------------................. 724
CP000325          677 .................G.............------------................. 724
CP000325          677 ...............................------------................. 724
CP000325          677 ...............................------------................. 724
CP000325          677 ...............................------------................. 724
CP000325          677 ...............................------------................. 724
CP000325          677 ...............................------------................. 724
CP000325          677 ...............................------------................. 724
CP000325          677 ...............................------------................. 724
CP000325          677 ............................A..------------................. 724
CP000325          677 ...........................A...------------................. 724
CP000325          677 ...........................A...------------................. 724
CP000325          677 ...............................------CACCGC................. 730
CP000325          677 ...............................------------................. 724
CP000325          677 ...C.......................A...------------................. 724
CP000325          677 ...........................A...------------................. 724
CP000325          679 ...............................------------................. 726
CP000325          677 ...............................------------................. 724
CP000325          677 ...........................A...------------................. 724
CP000325          677 ...........................A...------------................. 724
CP000325          677 ...............................------------................. 724
CP000325          677 ...............................------------................. 724
CP000325          677 ...........................A...------------................. 724
CP000325          677 ...............................------------................. 724
CP000325          677 ...............................------------................. 724
CP000325          697 ...............................------------................. 744
CP000325          677 ...........................A...------------................. 724
CP000325          677 ...........................A...------------................. 724
CP000325          677 ...............................------------................. 724
CP000325          677 ...............................------------................. 724
CP000325          677 ...............................------------................. 724
CP000325          677 ...............................------------................. 724
CP000325          677 ...............................------------................. 724
CP000325          677 ...........................A...------------................. 724
CP000325          677 ...............................------------................. 724
CP000325          677 ...............................------------................. 724
CP000325          677 ...............................------------................. 724
CP000325          677 ...............................------------................. 724
CP000325          677 ...............................------------................. 724
CP000325          677 ...............................------------................. 724
CP000325          677 ...........................A...------------................. 724
CP000325          677 ...............................------------................. 724
CP000325          677 ...............................------------................. 724
CP000325          677 ...............................------------................. 724
CP000325          677 ...............................------------................. 724
CP000325          677 ...............................------------................. 724
CP000325          677 ...............................------------................. 724
CP000325          677 ...............................------------................. 724
CP000325          677 ...............................------------................. 724
CP000325          347 ...............................------------................. 394
CP000325          677 ...............................------------................. 724
CP000325          677 ...............................------------................. 724
CP000325          677 ...............................------------................. 724
CP000325          677 ...............................------------................. 724
CP000325          677 ...............................------------................. 724
CP000325          677 ...............................------------................. 724
CP000325          677 ...............................------------................. 724
CP000325          677 ...............................------------................. 724
CP000325          677 ...........................A...------------................. 724
CP000325          677 ...............................------------................. 724
CP000325          677 ...............................------------................. 724
CP000325          677 ...............................------------................. 724
CP000325          677 ............................A..------------................. 724
CP000325          677 ...............................------------................. 724
CP000325          677 ...............................------------................. 724
CP000325          677 ...............................------------................. 724
```

```
CP000325              677 ...............................------------................. 724
CP000325              677 ...............................------------................. 724
CP000325              677 ...............................------------................. 724
CP000325              677 ...C...........................------------................. 724
CP000325              677 ............C..............A...------------................. 724
CP000325              677 ...........................A...------------................. 724
CP000325              677 ............C..................------------................. 724
CP000325              197 ------------------------------------------------------------ 197

(query)MUL_0099.prj   725 GCAGCGTGGAGGTGGTCTATGCCATCTGCAGCCTGCCGTTCGAGCACGCCCGCCCTACCG 784
CP000325              173 ------------------------------------------------------------ 173
CP000325              725 ......C................................................G.... 784
CP000325              725 .......................................................G.... 784
CP000325              725 ............................................................ 784
CP000325              726 ............................................................ 785
CP000325              727 ............................................................ 786
CP000325              725 A........................................................... 784
CP000325              725 ...............................T............................ 784
CP000325              725 ............................................................ 784
CP000325              725 ............................................................ 784
CP000325              725 ............................................................ 784
CP000325              725 ............................................................ 784
CP000325              725 ............................................................ 784
CP000325              725 ............................................................ 784
CP000325              725 ............................................................ 784
CP000325              725 ............................................................ 784
CP000325              725 ............................................................ 784
CP000325              725 ............................................................ 784
CP000325              725 ............................................................ 784
CP000325              725 ............................................................ 784
CP000325              724 .................................C.......................... 783
CP000325              725 ............................................................ 784
CP000325              193 ------------------------------------------------------------ 193
CP000325              564 ............................................................ 623
CP000325               61 ------------------------------------------------------------ 61
CP000325              558 ............................................................ 617
CP000325              725 ............................................................ 784
CP000325              725 ............................................................ 784
CP000325              725 ............................................................ 784
CP000325              725 ............................................................ 784
CP000325              725 ............................................................ 784
CP000325              725 ............................................................ 784
CP000325              725 ............................................................ 784
CP000325              724 ............................................................ 783
CP000325              725 ............................................................ 784
CP000325              725 ............................................................ 784
CP000325              725 ............................................................ 784
CP000325              725 ............................................................ 784
CP000325              725 A........................................................... 784
CP000325              725 ............................................................ 784
CP000325              725 ............................................................ 784
CP000325              725 ............................................................ 784
CP000325              725 ............................................................ 784
CP000325              725 ............................................................ 784
CP000325              725 ............................................................ 784
CP000325              725 ............................................................ 784
CP000325              725 ............................................................ 784
CP000325              725 ............................................................ 784
CP000325              725 .........................T.................................. 784
CP000325              725 ............................................................ 784
CP000325              725 ............................................................ 784
CP000325              725 ............................................................ 784
CP000325              725 ............................................................ 784
CP000325              725 .........................T.................................. 784
CP000325              725 ............................................................ 784
CP000325              725 ............................................................ 784
CP000325              725 ............................................................ 784
CP000325              725 ............................................................ 784
CP000325              725 ............................................................ 784
CP000325              725 ............................................................ 784
CP000325              725 ............................................................ 784
CP000325              725 A........................................................... 784
CP000325              725 ............................................................ 784
CP000325              737 ............................................................ 796
CP000325              724 ............................................................ 783
CP000325              725 ............................................................ 784
CP000325              725 ............................................................ 784
```

```
CP000325    725 ............................................................ 784
CP000325    725 ............................................................ 784
CP000325    725 ............................................................ 784
CP000325    725 .........................T.................................. 784
CP000325    725 ............................................................ 784
CP000325    725 ............................................................ 784
CP000325    725 ............................................................ 784
CP000325    724 ............................................................ 783
CP000325    523 ------------------------------------------------------------ 523
CP000325    266 ............................................................ 325
CP000325    725 ............................................................ 784
CP000325    725 ............................................................ 784
CP000325    725 ............................................................ 784
CP000325      0 ------------------------------------------------------------ 0
CP000325    725 ............................................................ 784
CP000325    618 ------------------------------------------------------------ 618
CP000325    154 ............................................................ 213
CP000325    725 ............................................................ 784
CP000325    725 ............................................................ 784
CP000325    725 ............................................................ 784
CP000325    725 ............................................................ 784
CP000325    725 ............................................................ 784
CP000325    725 ............................................................ 784
CP000325    725 ............................................................ 784
CP000325    725 ............................................................ 784
CP000325    725 ............................................................ 784
CP000325    725 ............................................................ 784
CP000325    725 A........................................................... 784
CP000325    725 ............................................................ 784
CP000325    725 ............................................................ 784
CP000325    725 ............................................................ 784
CP000325    725 .........................T.................................. 784
CP000325    725 ............................................................ 784
CP000325    725 ............................................................ 784
CP000325    725 ............................................................ 784
CP000325    725 ............................................................ 784
CP000325    725 ............................................................ 784
CP000325    725 ............................................................ 784
CP000325    725 ............................................................ 784
CP000325    725 ............................................................ 784
CP000325    725 ............................................................ 784
CP000325    725 A........................................................... 784
CP000325    725 ............................................................ 784
CP000325    725 ............................................................ 784
CP000325    725 ............................................................ 784
CP000325    725 .........................T.................................. 784
CP000325    725 ............................................................ 784
CP000325    725 ............................................................ 784
CP000325    725 ............................................................ 784
CP000325    725 .................................C.......................... 784
CP000325    725 ............................................................ 784
CP000325    725 ............................................................ 784
CP000325    724 ...C........................................................ 783
CP000325    725 ............................................................ 784
CP000325    725 A........................................................... 784
CP000325    725 ............................................................ 784
CP000325    724 ............................................................ 783
CP000325    746 A........................................................... 805
CP000325    725 ............................................................ 784
CP000325      0 ------------------------------------------------------------ 0
CP000325    725 ............................................................ 784
CP000325    725 ............................................................ 784
CP000325    725 ............................................................ 784
CP000325    725 ............................................................ 784
CP000325    725 ............................................................ 784
CP000325    725 ............................................................ 784
CP000325    725 ............................................................ 784
CP000325    725 ............................................................ 784
CP000325    725 ............................................................ 784
CP000325    725 ............................................................ 784
CP000325    725 ............................................................ 784
CP000325    725 A........................................................... 784
CP000325    725 ............................................................ 784
CP000325    725 ............................................................ 784
CP000325    725 ............................................................ 784
CP000325    725 ............................................................ 784
CP000325    725 ............................................................ 784
```

```
CP000325            725 ............................................................ 784
CP000325            725 ............................................................ 784
CP000325            725 ............................................................ 784
CP000325            725 ............................................................ 784
CP000325            725 ............................................................ 784
CP000325            725 ............................................................ 784
CP000325            725 ............................................................ 784
CP000325            731 ............................................................ 790
CP000325            725 ............................................................ 784
CP000325            725 ............................................................ 784
CP000325            725 A........................................................... 784
CP000325            727 ............................................................ 786
CP000325            725 ...................................................A........ 784
CP000325            725 ............................................................ 784
CP000325            725 ........................................T................... 784
CP000325            725 ............................................................ 784
CP000325            725 ............................................................ 784
CP000325            725 ............................................................ 784
CP000325            725 ............................................................ 784
CP000325            725 ............................................................ 784
CP000325            745 ............................................................ 804
CP000325            725 ............................................................ 784
CP000325            725 ............................................................ 784
CP000325            725 ............................................................ 784
CP000325            725 ............................................................ 784
CP000325            725 ............................................................ 784
CP000325            725 ............................................................ 784
CP000325            725 ............................................................ 784
CP000325            725 ............................................................ 784
CP000325            725 ............................................................ 784
CP000325            725 ............................................................ 784
CP000325            725 ............................................................ 784
CP000325            725 ............................................................ 784
CP000325            725 ...................................................A........ 784
CP000325            725 ............................................................ 784
CP000325            725 ............................................................ 784
CP000325            725 ............................................................ 784
CP000325            725 ............................................................ 784
CP000325            725 ............................................................ 784
CP000325            725 ............................................................ 784
CP000325            725 ............................................................ 784
CP000325            725 ............................................................ 784
CP000325            725 ............................................................ 784
CP000325            395 ............................................................ 454
CP000325            725 ............................................................ 784
CP000325            725 ............................................................ 784
CP000325            725 ............................................................ 784
CP000325            725 ............................................................ 784
CP000325            725 ............................................................ 784
CP000325            725 ............................................................ 784
CP000325            725 ............................................................ 784
CP000325            725 ............................................................ 784
CP000325            725 .......................................................G.... 784
CP000325            725 ............................................................ 784
CP000325            725 ...C........................................................ 784
CP000325            725 ............................................................ 784
CP000325            725 ............................................................ 784
CP000325            725 ............................................................ 784
CP000325            725 ............................................................ 784
CP000325            725 ............................................................ 784
CP000325            725 ............................................................ 784
CP000325            725 ............................................................ 784
CP000325            725 ........................................T................... 784
CP000325            725 A........................................................... 784
CP000325            725 ............................................................ 784
CP000325            725 ............................................................ 784
CP000325            725 ............................................................ 784
CP000325            197 ------------------------------------------------------------ 197

(query)MUL_0099.prj 785 CGATCATGACCTGGATGC------------------GTCAACACTGGGGAATCGAGAACA 826
CP000325            173 ------------------------------------------------------------ 173
CP000325            785 ..................------------------..........C............. 826
CP000325            785 ..................------------------..........C............. 826
CP000325            785 ..................------------------..........C............. 826
CP000325            786 ..................------------------........................ 827
CP000325            787 ..................------------------..........C............. 828
CP000325            785 ..................------------------..........C............. 826
```

```
CP000325             785 ..................------------------..........C............. 826
CP000325             785 ..................------------------........................ 826
CP000325             785 ..................------------------........................ 826
CP000325             785 ..................------------------........................ 826
CP000325             785 ..................------------------..........C............. 826
CP000325             785 ..................------------------..........C............. 826
CP000325             785 ..................------------------........G............... 826
CP000325             785 ..................------------------........................ 826
CP000325             785 ..................------------------..........C............. 826
CP000325             785 ..................------------------..........C............. 826
CP000325             785 ..................------------------..........C............. 826
CP000325             785 ..................------------------..........C............. 826
CP000325             785 ..................------------------..........C............. 826
CP000325             784 ..................------------------..........C............. 825
CP000325             785 ..................------------------..........C............. 826
CP000325             193 ------------------------------------------------------------ 193
CP000325             624 ..................------------------..........C............. 665
CP000325              61 ------------------------------------------------------------ 61
CP000325             618 ..................------------------..........C............. 659
CP000325             785 ..................------------------..........C............. 826
CP000325             785 ..................------------------..........C............. 826
CP000325             785 ..................------------------..........C............. 826
CP000325             785 ..................------------------..........C............. 826
CP000325             785 ..................------------------..........C............. 826
CP000325             785 ..................------------------..........C............. 826
CP000325             785 ..................------------------..........C............. 826
CP000325             784 ..................------------------..........C............. 825
CP000325             785 ..................------------------........................ 826
CP000325             785 ..................------------------..........C............. 826
CP000325             785 ..................------------------........................ 826
CP000325             785 ..................------------------..........C............. 826
CP000325             785 ..................------------------........................ 826
CP000325             785 ..................------------------........................ 826
CP000325             785 ..................------------------........................ 826
CP000325             785 ..................------------------..........C............. 826
CP000325             785 ..................------------------........G............... 826
CP000325             785 ..................------------------........G............... 826
CP000325             785 ........G.........------------------........................ 826
CP000325             785 ..................------------------..........C............. 826
CP000325             785 ..................------------------..........C............. 826
CP000325             785 ..................------------------..........C............. 826
CP000325             785 ..................------------------..........C............. 826
CP000325             785 ..................------------------..........C............. 826
CP000325             785 ..................------------------..........C............. 826
CP000325             785 ..................------------------..........C............. 826
CP000325             785 ..................------------------..........C............. 826
CP000325             785 ..................------------------..........C............. 826
CP000325             785 ..................------------------..........C............. 826
CP000325             785 ..................------------------..........C............. 826
CP000325             785 ..................------------------..........C............. 826
CP000325             785 ..................------------------..........C............. 826
CP000325             785 ..................------------------..........C............. 826
CP000325             785 ..................------------------........................ 826
CP000325             785 ..................------------------..........C............. 826
CP000325             785 ..................------------------..........C............. 826
CP000325             785 ..................------------------........................ 826
CP000325             797 ..................------------------..........C............. 838
CP000325             784 ..................------------------..........C............. 825
CP000325             785 ..................------------------..........C............. 826
CP000325             785 ..................------------------..........C............. 826
CP000325             785 ..................------------------........................ 826
CP000325             785 ..................------------------..........C............. 826
CP000325             785 ..................------------------..........C............. 826
CP000325             785 ..................------------------..........C............. 826
CP000325             785 ..................------------------........G............... 826
CP000325             785 ..................------------------........................ 826
CP000325             785 ..................------------------..........C............. 826
CP000325             784 ..................------------------........................ 825
CP000325             523 ------------------------------------------------------------ 523
CP000325             326 ..................------------------........................ 367
CP000325             785 ..................------------------........................ 826
CP000325             785 ..................------------------........................ 826
CP000325             785 ..................------------------........................ 826
CP000325               1 -------------------------------------....................... 23
CP000325             785 ..................------------------..........C............. 826
CP000325             618 ------------------------------------------------------------ 618
CP000325             214 ..................------------------........................ 255
```

```
CP000325            785 ..................------------------........G............... 826
CP000325            785 ..................------------------..........C............. 826
CP000325            785 ..................------------------..........C............. 826
CP000325            785 ..................------------------........................ 826
CP000325            785 ..................------------------..........C............. 826
CP000325            785 T.................------------------..........C............. 826
CP000325            785 ..................------------------..........C........A.... 826
CP000325            785 ..................------------------..........C............. 826
CP000325            785 ..................------------------........................ 826
CP000325            785 ..................------------------..........C............. 826
CP000325            785 ..................------------------..........C............. 826
CP000325            785 ..................------------------........................ 826
CP000325            785 ..................------------------..........C............. 826
CP000325            785 ..................------------------..........C............. 826
CP000325            785 ..................------------------..........C............. 826
CP000325            785 ..................------------------........................ 826
CP000325            785 ..................------------------..........C............. 826
CP000325            785 ..................------------------..........C............. 826
CP000325            785 ..................------------------..........C............G 826
CP000325            785 ..................------------------..........C............. 826
CP000325            785 ..................------------------..........C............. 826
CP000325            785 ..................------------------..........C............. 826
CP000325            785 ..................------------------..........C............. 826
CP000325            785 ..................------------------..........C............. 826
CP000325            785 ..................------------------..........C............. 826
CP000325            785 ..................------------------..........C............. 826
CP000325            785 ..................------------------..........C............. 826
CP000325            785 ..................------------------..........C............. 826
CP000325            785 ..................------------------..........C............. 826
CP000325            785 ..................------------------..........C............. 826
CP000325            785 ..................------------------..........C............. 826
CP000325            785 ..................------------------..........C............. 826
CP000325            785 ..................------------------..........C............. 826
CP000325            785 ..................------------------........G............... 826
CP000325            785 ..................------------------..........C............. 826
CP000325            784 ..................------------------........G............... 825
CP000325            785 ..................------------------..........CA............ 826
CP000325            785 ..................------------------..........C............. 826
CP000325            785 ..G...............------------------..........C............. 826
CP000325            784 ..................------------------..........C............. 825
CP000325            806 ..................------------------........................ 847
CP000325            785 ..................------------------..........C............. 826
CP000325              0 ------------------------------------------------------------ 0
CP000325            785 ..................------------------...............C........ 826
CP000325            785 ..................------------------...............C........ 826
CP000325            785 ..................------------------...............C........ 826
CP000325            785 ..................------------------..........C............. 826
CP000325            785 ..................------------------........................ 826
CP000325            785 ..................------------------..........C............. 826
CP000325            785 ..................------------------..........C............. 826
CP000325            785 ..................------------------..........C............. 826
CP000325            785 ..................------------------..........C............. 826
CP000325            785 ..................------------------..........C............. 826
CP000325            785 ..................------------------..........C............. 826
CP000325            785 ..................------------------..........C............. 826
CP000325            785 ..................------------------..........C............. 826
CP000325            785 ..................------------------..........C............. 826
CP000325            785 ..................------------------..........C............. 826
CP000325            785 ..................------------------..........C............. 826
CP000325            785 ........G.........------------------........................ 826
CP000325            785 ..................------------------........................ 826
CP000325            785 ..................------------------........................ 826
CP000325            785 ..................------------------........................ 826
CP000325            785 ..................------------------........................ 826
CP000325            785 ..................------------------..........C............. 826
CP000325            785 ..................------------------..........C............. 826
CP000325            785 ............T.....------------------..........C............. 826
CP000325            791 ..................------------------........G............... 832
CP000325            785 ..................------------------..........C............. 826
CP000325            785 ..................------------------..........C............. 826
CP000325            785 ..................------------------..........C............. 826
CP000325            787 ..................------------------..........C............. 828
CP000325            785 ..................------------------..........C............. 826
CP000325            785 ..................------------------........................ 826
CP000325            785 ..................------------------........................ 826
CP000325            785 ..................------------------..........C............. 826
CP000325            785 ..................------------------..........C............. 826
```

```
CP000325             785 ..................------------------..........C............. 826
CP000325             785 ..................------------------..........C............. 826
CP000325             785 T.................------------------..........C............. 826
CP000325             805 ..................------------------........................ 846
CP000325             785 ..................------------------..........C............. 826
CP000325             785 ............T.....------------------..........C............. 826
CP000325             785 ..................------------------..........C............. 826
CP000325             785 ..................------------------..........C............. 826
CP000325             785 ..................------------------..........C............. 826
CP000325             785 ..................------------------..........C............. 826
CP000325             785 ..................------------------..........C............. 826
CP000325             785 ..................------------------..........C............. 826
CP000325             785 ..................------------------..........C............. 826
CP000325             785 ..................------------------..........C............. 826
CP000325             785 ..................------------------..........C............. 826
CP000325             785 ..................------------------..........C............. 826
CP000325             785 ..................------------------..........C............. 826
CP000325             785 ..................GTCAACACTGCGGAATCG........................ 844
CP000325             785 ..................------------------..........C............. 826
CP000325             785 ..................------------------..........C............. 826
CP000325             785 ..................------------------..........C............. 826
CP000325             785 ..................------------------..........C............. 826
CP000325             785 ..................------------------..........C............. 826
CP000325             785 ..................------------------........................ 826
CP000325             785 ..................------------------..........C............. 826
CP000325             785 ..................------------------..........C............. 826
CP000325             455 ..................------------------..........C............. 496
CP000325             785 ..................------------------..........C............. 826
CP000325             785 ..................------------------..........C............. 826
CP000325             785 ..................------------------..........C............. 826
CP000325             785 ..................------------------..........C............. 826
CP000325             785 ..................------------------..........C............. 826
CP000325             785 ..................------------------..........C............. 826
CP000325             785 ..................------------------..........C............. 826
CP000325             785 ..................------------------..........C............. 826
CP000325             785 ..................------------------..........C............. 826
CP000325             785 ..................------------------..........C............. 826
CP000325             785 ..................------------------..........C............. 826
CP000325             785 ..................------------------..........C............. 826
CP000325             785 ..................------------------..........C............. 826
CP000325             785 ..................------------------..........C............. 826
CP000325             785 ..................------------------..........C............. 826
CP000325             785 ..................------------------..........C............. 826
CP000325             785 ..................------------------........................ 826
CP000325             785 ..................------------------........G.C............. 826
CP000325             785 ..................------------------.......................G 826
CP000325             785 ..................------------------.......................G 826
CP000325             785 ..................------------------..........C............. 826
CP000325             785 ..................------------------..........C............. 826
CP000325             785 ..................------------------..........C............. 826
CP000325             785 ..................GTCAACACTGGGGAATCG........................ 844
CP000325             197 ------------------------------------------------------------ 197

(query)MUL_0099.prj  827 GCCTGCACTGGATACGCGACGTCACCTTCGACGAAGACCGTCACAGGGCACATACCGGAA 886
CP000325             173 ------------------------------------------------------------ 173
CP000325             827 A..............A...........................................C 886
CP000325             827 A..............A...........................A...C...........C 886
CP000325             827 ...........................................................C 886
CP000325             828 ............................................................ 887
CP000325             829 A.......................................................A... 888
CP000325             827 ...........................................A...C............ 886
CP000325             827 ............................................................ 886
CP000325             827 ..............................................A............. 886
CP000325             827 ..............................................A............. 886
CP000325             827 ...........................................A...C...........C 886
CP000325             827 ............................................................ 886
CP000325             827 ............................................................ 886
CP000325             827 ............................................................ 886
CP000325             827 ...........................................................C 886
CP000325             827 ...........................................................C 886
CP000325             827 ...........................................................C 886
CP000325             827 ...........................................................C 886
CP000325             827 ...........................................................C 886
CP000325             827 ...........................................................C 886
CP000325             826 C..........................................................C 885
CP000325             827 ...........................................................C 886
CP000325             193 ------------------------------------------------------------ 193
CP000325             666 ...............A...........................................C 725
```

```
CP000325                 61 ------------------------------------------------------------ 61
CP000325                660 ..........C................................A...C...........C 719
CP000325                827 ...........................................................C 886
CP000325                827 ............................................................ 886
CP000325                827 ............................................................ 886
CP000325                827 ...........................................................C 886
CP000325                827 ...........................................................C 886
CP000325                827 ...........................................................C 886
CP000325                827 ...........................................A...C...........C 886
CP000325                826 ...........................................A...A............ 885
CP000325                827 ...............A...........................A................ 886
CP000325                827 ............................................................ 886
CP000325                827 ...................................T.......A...C............ 886
CP000325                827 ............................................................ 886
CP000325                827 ...........................................................C 886
CP000325                827 ............................................................ 886
CP000325                827 ...............A............................................ 886
CP000325                827 ...........................................................C 886
CP000325                827 ...........................................A...C...........C 886
CP000325                827 ...........................................A...C...........C 886
CP000325                827 ...........................................A...C............ 886
CP000325                827 .............................T.................C............ 886
CP000325                827 .............................T.............A...C............ 886
CP000325                827 ...........................................................C 886
CP000325                827 ............................................................ 886
CP000325                827 ............................................................ 886
CP000325                827 ...............................................C............ 886
CP000325                827 ...............................................A............ 886
CP000325                827 ............................................................ 886
CP000325                827 ............................................................ 886
CP000325                827 ...........................................A................ 886
CP000325                827 A..............A..............................A............. 886
CP000325                827 ...............................................C............ 886
CP000325                827 ...............................................C............ 886
CP000325                827 ............................................................ 886
CP000325                827 ............................................................ 886
CP000325                827 ............................................................ 886
CP000325                827 ..............................................A............. 886
CP000325                827 ...........................................A................ 886
CP000325                839 ...........................................A...C...........C 898
CP000325                826 ............................................................ 885
CP000325                827 ............................................................ 886
CP000325                827 ...........................................A...C............ 886
CP000325                827 ...........................................A................ 886
CP000325                827 ...............................................C............ 886
CP000325                827 ............................................................ 886
CP000325                827 ............................................................ 886
CP000325                827 ...........................................................C 886
CP000325                827 ...........................................A...............C 886
CP000325                827 ...........................................A...A...........C 886
CP000325                826 ...........................................A...C...........C 885
CP000325                523 ------------------------------------------------------------ 523
CP000325                368 ...........................................A...............C 427
CP000325                827 ...........................................A................ 886
CP000325                827 ...........................................A................ 886
CP000325                827 ...........................................A................ 886
CP000325                 24 ...........................................A................ 83
CP000325                827 ............................................................ 886
CP000325                618 ------------------------------------------------------------ 618
CP000325                256 ..................C........................A................ 315
CP000325                827 ............................................................ 886
CP000325                827 ...........................................A................ 886
CP000325                827 ............................................................ 886
CP000325                827 ............................................................ 886
CP000325                827 ............................................................ 886
CP000325                827 ............................................................ 886
CP000325                827 ...........................................A................ 886
CP000325                827 ............................................................ 886
CP000325                827 ............................................................ 886
CP000325                827 ............................................................ 886
CP000325                827 ............................................................ 886
CP000325                827 ............................................................ 886
CP000325                827 ............................................................ 886
CP000325                827 ............................................................ 886
CP000325                827 ................T........................................... 886
CP000325                827 ............................................................ 886
CP000325                827 ............................................................ 886
```

CP000325	827	...............A..	886
CP000325	827	....A................	886
CP000325	827	....	886
CP000325	827	....	886
CP000325	827	...............A..	886
CP000325	827	....	886
CP000325	827	....	886
CP000325	827	....	886
CP000325	827	....	886
CP000325	827	...............A..	886
CP000325	827	....	886
CP000325	827	....	886
CP000325	827	....	886
CP000325	827	...............A..	886
CP000325	827	....A................	886
CP000325	827	C...	886
CP000325	827	....	886
CP000325	827	....	886
CP000325	826	....A................	885
CP000325	827	....A................	886
CP000325	827	....	886
CP000325	827	....A................	886
CP000325	826	A...	885
CP000325	848	....	907
CP000325	827	....	886
CP000325	1	---...............	15
CP000325	827	A..............A...............................C........A...	886
CP000325	827	A..............A...........................A...C........A...	886
CP000325	827	A..............A...........................A...C........A...	886
CP000325	827	....A...C............	886
CP000325	827	.....................................T.....A...C............	886
CP000325	827	A..............A...........................A...C...........C	886
CP000325	827	A..............A.............T.............A...C........A...	886
CP000325	827	A..A...C........A...	886
CP000325	827	...............A...........................A...C............	886
CP000325	827	A..............A...........................A...C............	886
CP000325	827	C..A...C............	886
CP000325	827	....A...C...........C	886
CP000325	827	....A...C............	886
CP000325	827	A..............A...........................A...C...........C	886
CP000325	827	A..............A...........................A...C...........C	886
CP000325	827	A..............A...........................A...C...........C	886
CP000325	827	....A...C............	886
CP000325	827	....A...C............	886
CP000325	827	....A...C............	886
CP000325	827	...............A...........................A...C............	886
CP000325	827	....A...C............	886
CP000325	827	....A...C............	886
CP000325	827	....A...C...........C	886
CP000325	827	...............A............G..............A...C........A...	886
CP000325	833	....A...C........A...	892
CP000325	827	....A...C........A..C	886
CP000325	827	....A...C........A...	886
CP000325	827	....A...C............	886
CP000325	829	..........C................................A...C............	888
CP000325	827	....A...A............	886
CP000325	827	....A...C............	886
CP000325	827	....A...C............	886
CP000325	827	....A...C............	886
CP000325	827	....A...C............	886
CP000325	827	....A...C............	886
CP000325	827	....A...C........A...	886
CP000325	827	....A...C...........C	886
CP000325	847	....A...C............	906
CP000325	827	....A...C............	886
CP000325	827	...............A...........................A...C........A...	886
CP000325	827	....A...C...........C	886
CP000325	827	....A...C............	886
CP000325	827	....A...C............	886
CP000325	827	....A...C............	886
CP000325	827	....A...C............	886
CP000325	827	....A...C........A...	886
CP000325	827	....A...C............	886
CP000325	827	....A...C........A...	886
CP000325	827	....A...C........A...	886
CP000325	827	....A...C............	886
CP000325	827	....A...C............	886

```
CP000325              845 ...........................................A...C............ 904
CP000325              827 ...............A...........................A...C............ 886
CP000325              827 ...........................................A...C...........C 886
CP000325              827 ...........................................A...C...........C 886
CP000325              827 ...........................................A...C............ 886
CP000325              827 ...........................................A...C............ 886
CP000325              827 ...........................................A...C............ 886
CP000325              827 ...........................................A...C............ 886
CP000325              827 ...........................................A...C............ 886
CP000325              497 ...........................................A...C............ 556
CP000325              827 ...........................................A...C............ 886
CP000325              827 ...........................................A...C............ 886
CP000325              827 ...........................................A...C............ 886
CP000325              827 ...........................................A...C............ 886
CP000325              827 ...........................................A...C............ 886
CP000325              827 ...........................................A...C............ 886
CP000325              827 ...........................................A...C............ 886
CP000325              827 ...........................................A...C............ 886
CP000325              827 ...........................................A...C............ 886
CP000325              827 ...........................................A...C........A... 886
CP000325              827 ...........................................A...C...........C 886
CP000325              827 ...........................................A...C...........C 886
CP000325              827 ...........................................A...C............ 886
CP000325              827 ...............A...........................A...C............ 886
CP000325              827 A..............A...........................A...C............ 886
CP000325              827 ............................................................ 886
CP000325              827 ...........................................................C 886
CP000325              827 ............................................................ 886
CP000325              827 ............................................................ 886
CP000325              827 ...........................................................C 886
CP000325              827 ...............A...........................A...C............ 886
CP000325              827 ...........................................A...C...........C 886
CP000325              845 ...........................................A................ 904
CP000325              197 ------------------------------------------------------------ 197

(query)MUL_0099.prj   887 ACGGCGCACAGGTCCTAGCAACGCTACGCAACACCGCGATCAATCTGCACCGCCTCAACG 946
CP000325              173 ------------------------------------------------------------ 173
CP000325              887 ......................A..................................... 946
CP000325              887 ......................A..................................... 946
CP000325              887 ..........................................G................. 946
CP000325              888 ..........................................G................. 947
CP000325              889 ............................................................ 948
CP000325              887 ......................A..................................... 946
CP000325              887 ............................................................ 946
CP000325              887 ............................................................ 946
CP000325              887 ............................................................ 946
CP000325              887 ............................................................ 946
CP000325              887 ............................................................ 946
CP000325              887 .......................T.................................... 946
CP000325              887 ............................................................ 946
CP000325              887 ....T....................................................... 946
CP000325              887 ............................................................ 946
CP000325              887 ......................................G..................... 946
CP000325              887 ....T....................................................... 946
CP000325              887 ....T....................................................... 946
CP000325              887 ....T....................................................... 946
CP000325              886 ............................................................ 945
CP000325              887 ............................................................ 946
CP000325              193 ------------------------------------------------------------ 193
CP000325              726 ............................................................ 785
CP000325               61 ------------------------------------------------------------ 61
CP000325              720 ............................................................ 779
CP000325              887 ............................................................ 946
CP000325              887 ............................................................ 946
CP000325              887 ............................................................ 946
CP000325              887 ............................................................ 946
CP000325              887 ............................................................ 946
CP000325              887 ............................................................ 946
CP000325              887 ....T....................................................... 946
CP000325              886 ............................................................ 945
CP000325              887 ....................................................-....... 945
CP000325              887 ............................................................ 946
CP000325              887 ............................................................ 946
CP000325              887 ............................................................ 946
CP000325              887 ............................................................ 946
CP000325              887 .......................................A.................... 946
CP000325              887 ............................................................ 946
```

```
CP000325          887 ............................................................ 946
CP000325          887 ............................................................ 946
CP000325          887 ............................................................ 946
CP000325          887 ............................................................ 946
CP000325          887 ............................................................ 946
CP000325          887 ............................................................ 946
CP000325          887 ....A....................................................... 946
CP000325          887 ....A....................................................... 946
CP000325          887 ....A....................................................... 946
CP000325          887 ............................................................ 946
CP000325          887 ............................................................ 946
CP000325          887 .............................G.............................. 946
CP000325          887 ............................................................ 946
CP000325          887 ......................................G..................... 946
CP000325          887 ............................................................ 946
CP000325          887 ............................................................ 946
CP000325          887 ............................................................ 946
CP000325          887 ....A....................................................... 946
CP000325          887 ............................................................ 946
CP000325          887 ............................................................ 946
CP000325          887 ......................A..................................... 946
CP000325          887 ............................................................ 946
CP000325          899 ............................................................ 958
CP000325          886 .............................G.............................. 945
CP000325          887 ............................................................ 946
CP000325          887 ............................................................ 946
CP000325          887 ............................................................ 946
CP000325          887 ............................................................ 946
CP000325          887 ............................................................ 946
CP000325          887 ............................................................ 946
CP000325          887 ............................................................ 946
CP000325          887 ............................................................ 946
CP000325          887 ............................................................ 946
CP000325          886 ............................................................ 945
CP000325          523 ------------------------------------------------------------ 523
CP000325          428 ............................................................ 487
CP000325          887 ............................................................ 946
CP000325          887 ............................................................ 946
CP000325          887 ............................................................ 946
CP000325           84 ............................................................ 143
CP000325          887 ............................................................ 946
CP000325          618 ------------------------------------------------------------ 618
CP000325          316 ............................................................ 375
CP000325          887 ............................................................ 946
CP000325          887 ............................................................ 946
CP000325          887 ............................................................ 946
CP000325          887 ............................................................ 946
CP000325          887 ............................................................ 946
CP000325          887 ............................................................ 946
CP000325          887 ............................................................ 946
CP000325          887 ............................................................ 946
CP000325          887 ............................................................ 946
CP000325          887 ............................................................ 946
CP000325          887 ............................................................ 946
CP000325          887 ............................................................ 946
CP000325          887 ............................................................ 946
CP000325          887 ............................................................ 946
CP000325          887 ............................................................ 946
CP000325          887 ............................................................ 946
CP000325          887 ............................................................ 946
CP000325          887 ............................................................ 946
CP000325          887 ............................................................ 946
CP000325          887 ............................................................ 946
CP000325          887 ............................................................ 946
CP000325          887 ............................................................ 946
CP000325          887 ............................................................ 946
CP000325          887 ............................................................ 946
CP000325          887 ............................................................ 946
CP000325          887 ............................................................ 946
CP000325          887 ............................................................ 946
CP000325          887 ............................................................ 946
CP000325          887 ............................................................ 946
CP000325          887 ............................................................ 946
CP000325          887 ............................................................ 946
CP000325          887 ............................................................ 946
CP000325          887 ............................................................ 946
CP000325          887 ............................................................ 946
```

```
CP000325          887 ............................................................ 946
CP000325          886 ............................................................ 945
CP000325          887 ............................................................ 946
CP000325          887 ............................................................ 946
CP000325          887 ............................................................ 946
CP000325          886 ............................................................ 945
CP000325          908 ............................................................ 967
CP000325          887 ............................................................ 946
CP000325           16 ............................................................ 75
CP000325          887 ............................................................ 946
CP000325          887 ............................................................ 946
CP000325          887 ......................A..................................... 946
CP000325          887 ...A........................................................ 946
CP000325          887 ..........................................G................. 946
CP000325          887 ......................A..................................... 946
CP000325          887 ............................................................ 946
CP000325          887 ............................................................ 946
CP000325          887 ............................................................ 946
CP000325          887 ............................................................ 946
CP000325          887 ............................................................ 946
CP000325          887 ............................................................ 946
CP000325          887 ............................................................ 946
CP000325          887 ............................................................ 946
CP000325          887 ............................................................ 946
CP000325          887 ............................................................ 946
CP000325          887 ............................................................ 946
CP000325          887 ..........................................G................. 946
CP000325          887 ............................................................ 946
CP000325          887 ..........................................G................. 946
CP000325          887 ............................................................ 946
CP000325          887 ............................................................ 946
CP000325          887 ............................................................ 946
CP000325          887 ............................................................ 946
CP000325          893 ............................................................ 952
CP000325          887 ..........................................G................. 946
CP000325          887 ............................................................ 946
CP000325          887 ............................................................ 946
CP000325          889 ............................................................ 948
CP000325          887 ............................................................ 946
CP000325          887 ............................................................ 946
CP000325          887 ............................................................ 946
CP000325          887 ............................................................ 946
CP000325          887 ..........................................G................. 946
CP000325          887 ............................................................ 946
CP000325          887 ............................................................ 946
CP000325          887 ............................................................ 946
CP000325          907 ............................................................ 966
CP000325          887 ............................................................ 946
CP000325          887 ............................................................ 946
CP000325          887 ............................................................ 946
CP000325          887 ............................................................ 946
CP000325          887 ............................................................ 946
CP000325          887 ............................................................ 946
CP000325          887 ............................................................ 946
CP000325          887 ............................................................ 946
CP000325          887 ............................................................ 946
CP000325          887 ............................................................ 946
CP000325          887 ............................................................ 946
CP000325          887 ...................................................--------- 937
CP000325          887 ............................................................ 946
CP000325          905 ............................................................ 964
CP000325          887 ...................................................A........ 946
CP000325          887 ............................................................ 946
CP000325          887 ............................................................ 946
CP000325          887 ............................................................ 946
CP000325          887 ............................................................ 946
CP000325          887 ............................................................ 946
CP000325          887 ............................................................ 946
CP000325          887 ............................................................ 946
CP000325          557 ............................................................ 616
CP000325          887 ............................................................ 946
CP000325          887 ..........................................G................. 946
CP000325          887 ............................................................ 946
CP000325          887 ............................................................ 946
CP000325          887 ............................................................ 946
CP000325          887 ..........................................G................. 946
CP000325          887 ............................................................ 946
```

```
CP000325             887 ............................................................ 946
CP000325             887 ............................................................ 946
CP000325             887 ............................................................ 946
CP000325             887 ............................................................ 946
CP000325             887 ............................................................ 946
CP000325             887 ............................................................ 946
CP000325             887 ............................................................ 946
CP000325             887 ............................................................ 946
CP000325             887 ............................................................ 946
CP000325             887 ....T....................................................... 946
CP000325             887 ............................................................ 946
CP000325             887 C........................................................... 946
CP000325             887 ............................................................ 946
CP000325             887 ............................................................ 946
CP000325             887 ............................................................ 946
CP000325             905 ......................................G...G..........G...... 964
CP000325             197 ------------------------------------------------------------ 197

(query)MUL_0099.prj  947 GCGCCGACAACATCGCCGACGCCTGCCGGATCA----------CCGCTTTGACCGCCAAC 996
CP000325             173 ------------------------------------------------------------ 173
CP000325             947 ...................A.............----------................. 996
CP000325             947 ...................A.............----------................. 996
CP000325             947 ...................A.............----------................. 996
CP000325             948 .................................----------................. 997
CP000325             949 ...................A.............----------................. 998
CP000325             947 ...................A.............----------................. 996
CP000325             947 ...................A.............----------................. 996
CP000325             947 ...................A.............----------................. 996
CP000325             947 ...................A.............----------................. 996
CP000325             947 ...................A.............----------................. 996
CP000325             947 ...................A.............----------................. 996
CP000325             947 ...................A.............----------................. 996
CP000325             947 ...................A.............----------................. 996
CP000325             947 ...................A.............----------................. 996
CP000325             947 .................................----------................. 996
CP000325             947 .................................----------................. 996
CP000325             947 ...................A.............----------................. 996
CP000325             947 ...................A.............----------................. 996
CP000325             947 ...................A.............----------................. 996
CP000325             946 ...................A.............----------................. 995
CP000325             947 ...................A.............----------................. 996
CP000325             193 ------------------------------------------------------------ 193
CP000325             786 ...................A.............----------................. 835
CP000325              61 ------------------------------------------------------------ 61
CP000325             780 ...................A.............----------................. 829
CP000325             947 ...................A.............----------................. 996
CP000325             947 ...................A.............----------................. 996
CP000325             947 ...................A.............----------................. 996
CP000325             947 ...................A.............----------................. 996
CP000325             947 ...................A.............----------................. 996
CP000325             947 ...................A.............----------................. 996
CP000325             947 ...................A.............----------................. 996
CP000325             946 ...................A.............----------................. 995
CP000325             946 .................................----------................. 995
CP000325             947 ...................A.............----------................. 996
CP000325             947 ...................A.............----------................. 996
CP000325             947 ...................A.............----------................. 996
CP000325             947 ...................A.............----------................. 996
CP000325             947 ...................A.............----------................. 996
CP000325             947 .................................----------................. 996
CP000325             947 .................................----------................. 996
CP000325             947 ...................A.............----------................. 996
CP000325             947 ...................A.............----------................. 996
CP000325             947 ...................A.............----------................. 996
CP000325             947 ...................A.............----------................. 996
CP000325             947 ...................A.............----------................. 996
CP000325             947 .................................----------................. 996
CP000325             947 .................................----------................. 996
CP000325             947 .................................----------................. 996
CP000325             947 ...................A.............----------................. 996
CP000325             947 ...................A.............----------................. 996
CP000325             947 ...................A.............----------................. 996
CP000325             947 ...................A.............----------................. 996
CP000325             947 ...................A.............----------................. 996
CP000325             947 .................................----------................. 996
CP000325             947 ...................A.............----------................. 996
CP000325             947 ...................A.............----------................. 996
```

```
CP000325    947 .................................-----------................ 996
CP000325    947 .................................-----------................ 996
CP000325    947 .................................-----------................ 996
CP000325    947 ...................A.............-----------................ 996
CP000325    947 ..................GA.............-----------................ 996
CP000325    959 ...................A.............-----------................ 1008
CP000325    946 ...................A.............-----------................ 995
CP000325    947 .................................-----------................ 996
CP000325    947 ...................A.............-----------................ 996
CP000325    947 ...................A.............-----------................ 996
CP000325    947 ...................A.............-----------................ 996
CP000325    947 ...................A...C.........-----------................ 996
CP000325    947 .................................-----------................ 996
CP000325    947 ...................A.............-----------................ 996
CP000325    947 ...................A.............-----------................ 996
CP000325    947 ...................A.............-----------................ 996
CP000325    946 ...................A.............-----------................ 995
CP000325    523 ------------------------------------------------------------ 523
CP000325    488 ...................A.............-----------................ 537
CP000325    947 ...................A.............-----------................ 996
CP000325    947 ...................A.............-----------................ 996
CP000325    947 ...................A.............-----------................ 996
CP000325    144 ...................A.............-----------................ 193
CP000325    947 ...................A.............-----------................ 996
CP000325    618 ------------------------------------------------------------ 618
CP000325    376 ...................A.............-----------................ 425
CP000325    947 ...................A.............-----------................ 996
CP000325    947 ...................A.............-----------................ 996
CP000325    947 ...................A.............-----------................ 996
CP000325    947 ...................A.............-----------................ 996
CP000325    947 ...................A.............-----------................ 996
CP000325    947 ...................A.............-----------................ 996
CP000325    947 ...................A.............-----------................ 996
CP000325    947 ...................A.............-----------................ 996
CP000325    947 ...................A.............-----------................ 996
CP000325    947 ...................A.............-----------................ 996
CP000325    947 ...................A.............-----------................ 996
CP000325    947 ...................A.............-----------................ 996
CP000325    947 ...................A.............-----------................ 996
CP000325    947 ...................A.............-----------................ 996
CP000325    947 ...................A.............-----------................ 996
CP000325    947 ...................A.............-----------................ 996
CP000325    947 ...................A.............-----------................ 996
CP000325    947 ...................A.............-----------................ 996
CP000325    947 ...................A.............-----------................ 996
CP000325    947 ...................A.............-----------................ 996
CP000325    947 ...................A.............-----------................ 996
CP000325    947 ...................A.............-----------................ 996
CP000325    947 ...................A.............-----------................ 996
CP000325    947 ...................A.............-----------................ 996
CP000325    947 ...................A.............-----------................ 996
CP000325    947 ...................A.............-----------................ 996
CP000325    947 ...................A.............-----------................ 996
CP000325    947 ...................A.............-----------................ 996
CP000325    947 ...................A.............-----------................ 996
CP000325    947 ...................A.............-----------................ 996
CP000325    947 ...................A.............-----------................ 996
CP000325    947 ...................A.............-----------................ 996
CP000325    947 ...................A.............-----------................ 996
CP000325    947 ...................A.............-----------................ 996
CP000325    947 ...................A.............-----------................ 996
CP000325    946 ...................A.............-----------................ 995
CP000325    947 ...................A.............CCGCTTTGAC................. 1006
CP000325    947 ...................A.............-----------................ 996
CP000325    947 ...................A.............-----------................ 996
CP000325    946 ...................A.............-----------................ 995
CP000325    968 ...................A.............-----------................ 1017
CP000325    947 ...................A.............-----------................ 996
CP000325     76 ...................A.............-----------................ 125
CP000325    947 ...................A.............-----------................ 996
CP000325    947 ...................A.............-----------................ 996
CP000325    947 ...................A.............-----------................ 996
CP000325    947 ...................A.............-----------................ 996
CP000325    947 ...................A.............-----------................ 996
CP000325    947 ...................A.............-----------................ 996
CP000325    947 ...................A.............-----------................ 996
CP000325    947 ...................A...C.........-----------................ 996
```

```
CP000325             947 ...................A.............-----------................. 996
CP000325             947 .................................-----------................. 996
CP000325             947 ...................A.............-----------................. 996
CP000325             947 ...................A.............-----------................. 996
CP000325             947 ...................A.............-----------................. 996
CP000325             947 ...................A.............-----------................. 996
CP000325             947 ...................A.............-----------................. 996
CP000325             947 ............C......A.............-----------................. 996
CP000325             947 ...................A.............-----------................. 996
CP000325             947 ...................A.............-----------................. 996
CP000325             947 ...................A.............-----------................. 996
CP000325             947 ...................A.............-----------................. 996
CP000325             947 ...................A.............-----------................. 996
CP000325             947 ...................A.............-----------................. 996
CP000325             947 ...................A.............-----------................. 996
CP000325             947 ...................A.............-----------................. 996
CP000325             953 ...................A.............-----------................. 1002
CP000325             947 ...................A.............-----------................. 996
CP000325             947 ...................A.............-----------................. 996
CP000325             947 ...................A.............-----------................. 996
CP000325             949 ...................A.............-----------................. 998
CP000325             947 ...................A.............-----------................. 996
CP000325             947 ...................A.............-----------................. 996
CP000325             947 ...................A.............-----------................. 996
CP000325             947 .................................-----------................. 996
CP000325             947 .................................-----------................. 996
CP000325             947 ...................A.............-----------................. 996
CP000325             947 .................................-----------................. 996
CP000325             947 ...................A.............-----------................. 996
CP000325             967 .................................-----------................. 1016
CP000325             947 ...................A.............-----------................. 996
CP000325             947 ...................A.............-----------................. 996
CP000325             947 ...................A.............-----------................. 996
CP000325             947 ...................A.............-----------................. 996
CP000325             947 ...................A.............-----------................. 996
CP000325             947 ...................A.............-----------................. 996
CP000325             947 ...................A.............-----------................. 996
CP000325             947 ...................A.............-----------................. 996
CP000325             947 ...................A.............-----------................. 996
CP000325             947 ...................A.............-----------................. 996
CP000325             947 ...................A.............-----------................. 996
CP000325             937 ------------------------------------------------------------- 937
CP000325             947 ...................A.............-----------................. 996
CP000325             965 ...................A.............-----------................. 1014
CP000325             947 ...................A.............-----------................. 996
CP000325             947 ...................A.............-----------................. 996
CP000325             947 ...................A.............-----------................. 996
CP000325             947 ...................A.............-----------................. 996
CP000325             947 ...................A.............-----------................. 996
CP000325             947 ...................A.............-----------................. 996
CP000325             947 ...................A.............-----------................. 996
CP000325             947 ...................A.............-----------................. 996
CP000325             617 ...................A.............-----------................. 666
CP000325             947 ...................A.............-----------................. 996
CP000325             947 .................................-----------................. 996
CP000325             947 ...................A.............-----------................. 996
CP000325             947 ...................A.............-----------................. 996
CP000325             947 ...................A.............-----------................. 996
CP000325             947 ...................A.............-----------................. 996
CP000325             947 ...................A.............-----------................. 996
CP000325             947 ...................A.............-----------................. 996
CP000325             947 ...................A.............-----------................. 996
CP000325             947 .................................-----------................. 996
CP000325             947 ...................A.............-----------................. 996
CP000325             947 ...................A.............-----------................. 996
CP000325             947 ...................A.............-----------................. 996
CP000325             947 ...................A.............-----------................. 996
CP000325             947 ...................A.............-----------................. 996
CP000325             947 ..A................A.............-----------................. 996
CP000325             947 ...................A.............-----------................. 996
CP000325             947 ...................A.............-----------................. 996
CP000325             947 ...................A.............-----------................. 996
CP000325             947 ...................A.............-----------................. 996
CP000325             947 ...................A.............-----------................. 996
CP000325             947 ...................A.............-----------................. 996
CP000325             965 ...................A.............-----------................. 1014
CP000325             197 ------------------------------------------------------------- 197
```

```
(query)MUL_0099.prj   997 CGCCGCCTAGACCTCCTCAATCCACAATTCCCCAGCTCACAAGCCTGCTAA   1047
CP000325              173 ---------------------------------------------------   173
CP000325              997 ...................................................   1047
CP000325              997 ...................................................   1047
CP000325              997 ....................C..............................   1047
CP000325              998 ...................................................   1048
CP000325              999 .............................T.....................   1049
CP000325              997 ...................................................   1047
CP000325              997 ...A.......................................T.......   1047
CP000325              997 ...............................................T...   1047
CP000325              997 ...................................................   1047
CP000325              997 ...................................................   1047
CP000325              997 ...A...............................................   1047
CP000325              997 ...................................................   1047
CP000325              997 ...A...............................................   1047
CP000325              997 ...................................................   1047
CP000325              997 ...................................................   1047
CP000325              997 ...................................................   1047
CP000325              997 ...................................................   1047
CP000325              997 ...................................................   1047
CP000325              997 ...................................................   1047
CP000325              996 ...................................................   1046
CP000325              997 ...................................................   1047
CP000325              193 ---------------------------------------------------   193
CP000325              836 ...................................................   886
CP000325               61 ---------------------------------------------------   61
CP000325              830 ...................................................   880
CP000325              997 ...................................................   1047
CP000325              997 ...A...............................................   1047
CP000325              997 ...A...............................................   1047
CP000325              997 ...................................................   1047
CP000325              997 ...................................................   1047
CP000325              997 ...................................................   1047
CP000325              997 ...................................................   1047
CP000325              996 ...................................................   1046
CP000325              996 ...................................................   1046
CP000325              997 ...................................T...............   1047
CP000325              997 ...................................................   1047
CP000325              997 T..................................................   1047
CP000325              997 ...................................................   1047
CP000325              997 ...................................................   1047
CP000325              997 ...................................................   1047
CP000325              997 ...................................................   1047
CP000325              997 ...................................................   1047
CP000325              997 ...................................................   1047
CP000325              997 ...................................................   1047
CP000325              997 ...........T.......................................   1047
CP000325              997 ...........T..........................G............   1047
CP000325              997 ...................................................   1047
CP000325              997 ...................................................   1047
CP000325              997 ...................................................   1047
CP000325              997 ...................................................   1047
CP000325              997 ...................................................   1047
CP000325              997 ...................................................   1047
CP000325              997 ..............................T....................   1047
CP000325              997 ...................................................   1047
CP000325              997 ...................................................   1047
CP000325              997 .............C.....................................   1047
CP000325              997 ...................................................   1047
CP000325              997 ...................................................   1047
CP000325              997 ...................................................   1047
CP000325              997 ...................................................   1047
CP000325              997 ...................................................   1047
CP000325              997 ...................................................   1047
CP000325             1009 ...................................................   1059
CP000325              996 ...................................................   1046
CP000325              997 ...................................................   1047
CP000325              997 ...................................................   1047
CP000325              997 ....................C..............................   1047
CP000325              997 ...................................................   1047
CP000325              997 ...................................................   1047
CP000325              997 ...................................................   1047
CP000325              997 ...................................................   1047
CP000325              997 ...................................................   1047
CP000325              997 ...................................................   1047
CP000325              996 ...................................................   1046
CP000325              523 ---------------------------------------------------   523
```

```
CP000325         538 ................................................... 588
CP000325         997 ................................................... 1047
CP000325         997 ................................................... 1047
CP000325         997 ................................................... 1047
CP000325         194 ................................................... 244
CP000325         997 .-................................................. 1046
CP000325         618 --------------------------------------------------- 618
CP000325         426 ................................................... 476
CP000325         997 ................................................... 1047
CP000325         997 ................................................... 1047
CP000325         997 ................................................... 1047
CP000325         997 ................................................... 1047
CP000325         997 ................................................... 1047
CP000325         997 ................................................... 1047
CP000325         997 ................................................... 1047
CP000325         997 ................................................... 1047
CP000325         997 ................................................... 1047
CP000325         997 ................................................... 1047
CP000325         997 ................................................... 1047
CP000325         997 ................................................... 1047
CP000325         997 ................................................... 1047
CP000325         997 ................................................... 1047
CP000325         997 ................................................... 1047
CP000325         997 ................................................... 1047
CP000325         997 ................................................... 1047
CP000325         997 ................................................... 1047
CP000325         997 ................................................... 1047
CP000325         997 ................................................... 1047
CP000325         997 ................................................... 1047
CP000325         997 ................................................... 1047
CP000325         997 ................................................... 1047
CP000325         997 ................................................... 1047
CP000325         997 ................................................... 1047
CP000325         997 ................................................... 1047
CP000325         997 ................................................... 1047
CP000325         997 ................................................... 1047
CP000325         997 ................................................... 1047
CP000325         997 ................................................... 1047
CP000325         997 ................................................... 1047
CP000325         997 ................................................... 1047
CP000325         997 ................................................... 1047
CP000325         997 ................................................... 1047
CP000325         997 ................................................... 1047
CP000325         997 ................................................... 1047
CP000325         996 ................................................... 1046
CP000325        1007 ................................................... 1057
CP000325         997 ................................................... 1047
CP000325         997 ................................................... 1047
CP000325         996 ................................................... 1046
CP000325        1018 ................................................... 1068
CP000325         997 .-................................................. 1046
CP000325         126 ................................................... 176
CP000325         997 ................................................... 1047
CP000325         997 ................................................... 1047
CP000325         997 ................................................... 1047
CP000325         997 ................................................... 1047
CP000325         997 T.................................................. 1047
CP000325         997 ................................................... 1047
CP000325         997 ......................T............................ 1047
CP000325         997 ................................................... 1047
CP000325         997 ....................C.............................. 1047
CP000325         997 ................................................... 1047
CP000325         997 ...A............................................... 1047
CP000325         997 ................................................... 1047
CP000325         997 ................................................... 1047
CP000325         997 ................................................... 1047
CP000325         997 ................................................... 1047
CP000325         997 ................................................... 1047
CP000325         997 T.................................................. 1047
CP000325         997 T.................................................. 1047
CP000325         997 ................................................... 1047
CP000325         997 ................................................... 1047
CP000325         997 ................................................... 1047
CP000325         997 ................................................... 1047
CP000325         997 ................................................... 1047
CP000325         997 ................................................... 1047
CP000325        1003 ................................................... 1053
CP000325         997 ................................................... 1047
```

```
CP000325          997 ..................................................          1047
CP000325          997 ..................................................          1047
CP000325          999 ..................................................          1049
CP000325          997 ..................................................          1047
CP000325          997 ..................................................          1047
CP000325          997 ..................................................          1047
CP000325          997 ..................................................          1047
CP000325          997 ..................................................          1047
CP000325          997 ..................................................          1047
CP000325          997 ...........T......................................          1047
CP000325          997 ..................................................          1047
CP000325         1017 ..................................................          1067
CP000325          997 ...........................................T......          1047
CP000325          997 ..................................................          1047
CP000325          997 ..................................................          1047
CP000325          997 ..................................................          1047
CP000325          997 ..................................................          1047
CP000325          997 ..................................................          1047
CP000325          997 ..................................................          1047
CP000325          997 ..................................................          1047
CP000325          997 ..................................................          1047
CP000325          997 ..................................................          1047
CP000325          997 ..................................................          1047
CP000325          997 ..................................................          1047
CP000325          937 --------------------------------------------------          937
CP000325          997 ..................................................          1047
CP000325         1015 ..................................................          1065
CP000325          997 ..................................................          1047
CP000325          997 ..................................................          1047
CP000325          997 ..................................................          1047
CP000325          997 ..................................................          1047
CP000325          997 ..................................................          1047
CP000325          997 ..................................................          1047
CP000325          997 ..................................................          1047
CP000325          997 ..................................................          1047
CP000325          667 ..................................................          717
CP000325          997 ..................................................          1047
CP000325          997 ..................................................          1047
CP000325          997 ..................................................          1047
CP000325          997 ....................C.............................          1047
CP000325          997 ..................................................          1047
CP000325          997 ..................................................          1047
CP000325          997 ..................................................          1047
CP000325          997 ..................................................          1047
CP000325          997 ..................................................          1047
CP000325          997 ..................................................          1047
CP000325          997 ..................................................          1047
CP000325          997 ..................................................          1047
CP000325          997 ..................................................          1047
CP000325          997 ..................................................          1047
CP000325          997 ..................................................          1047
CP000325          997 ..................................................          1047
CP000325          997 ..................................................          1047
CP000325          997 T.................................................          1047
CP000325          997 ..................................T...............          1047
CP000325          997 ..................................................          1047
CP000325          997 T.................................................          1047
CP000325          997 ....T.............................................          1047
CP000325         1015 ..................................................          1065
CP000325          197 --------------------------------------------------          197
```
